# Dental cell type atlas reveals stem and differentiated cell types in mouse and human teeth

**DOI:** 10.1038/s41467-020-18512-7

**Published:** 2020-09-23

**Authors:** Jan Krivanek, Ruslan A. Soldatov, Maria Eleni Kastriti, Tatiana Chontorotzea, Anna Nele Herdina, Julian Petersen, Bara Szarowska, Marie Landova, Veronika Kovar Matejova, Lydie Izakovicova Holla, Ulrike Kuchler, Ivana Vidovic Zdrilic, Anushree Vijaykumar, Anamaria Balic, Pauline Marangoni, Ophir D. Klein, Vitor C. M. Neves, Val Yianni, Paul T. Sharpe, Tibor Harkany, Brian D. Metscher, Marc Bajénoff, Mina Mina, Kaj Fried, Peter V. Kharchenko, Igor Adameyko

**Affiliations:** 1 Department of Molecular Neuroscience, Center for Brain Research, Medical University of Vienna, Vienna, Austria; 2 Department of Histology and Embryology, Faculty of Medicine, Masaryk University, Brno, Czech Republic; 3 Department of Biomedical Informatics, Harvard Medical School, Boston, MA USA; 4 Department of Physiology and Pharmacology, Karolinska Institutet, Stockholm, Sweden; 5 Institute of Animal Physiology and Genetics, CAS, Brno, Czech Republic; 6 Clinic of Stomatology, Institution Shared with St. Anne’s Faculty Hospital, Faculty of Medicine, Masaryk University, Brno, Czech Republic; 7 Department of Oral Biology, Medical University of Vienna, Vienna, Austria; 8 Department of Oral Surgery, Medical University of Vienna, Vienna, Austria; 9 Department of Craniofacial Sciences, School of Dental Medicine, University of Connecticut Health Center, Farmington, CT USA; 10 Research Program in Developmental Biology, Institute of Biotechnology, University of Helsinki, Helsinki, Finland; 11 Program in Craniofacial Biology and Department of Orofacial Sciences, University of California, San Francisco, CA USA; 12 Department of Pediatrics and Institute for Human Genetics, University of California, San Francisco, CA USA; 13 Centre for Craniofacial and Regenerative Biology, Faculty of Dentistry, Oral & Craniofacial Sciences. King’s College London, London, UK; 14 Department of Neuroscience, Karolinska Institutet, Stockholm, Sweden; 15 Department of Evolutionary Biology, University of Vienna, Vienna, Austria; 16 Centre d’Immunologie de Marseille-Luminy, Aix Marseille Université, INSERM, CNRS UMR, Marseille, France; 17 Department of Neuroimmunology, Center for Brain Research, Medical University of Vienna, Vienna, Austria

**Keywords:** Organogenesis, Stem-cell niche, Mesenchymal stem cells

## Abstract

Understanding cell types and mechanisms of dental growth is essential for reconstruction and engineering of teeth. Therefore, we investigated cellular composition of growing and non-growing mouse and human teeth. As a result, we report an unappreciated cellular complexity of the continuously-growing mouse incisor, which suggests a coherent model of cell dynamics enabling unarrested growth. This model relies on spatially-restricted stem, progenitor and differentiated populations in the epithelial and mesenchymal compartments underlying the coordinated expansion of two major branches of pulpal cells and diverse epithelial subtypes. Further comparisons of human and mouse teeth yield both parallelisms and differences in tissue heterogeneity and highlight the specifics behind growing and non-growing modes. Despite being similar at a coarse level, mouse and human teeth reveal molecular differences and species-specific cell subtypes suggesting possible evolutionary divergence. Overall, here we provide an atlas of human and mouse teeth with a focus on growth and differentiation.

## Abstract (web summary)

Unlike human teeth, mouse incisors grow throughout life, based on stem and progenitor cell activity. Here the authors generate single cell RNA-seq comparative maps of continuously-growing mouse incisor, non-growing mouse molar and human teeth, combined with lineage tracing to reveal dental cell complexity.

## Introduction

Mammalian teeth are formed by the ectoderm of the first pharyngeal arch and neural crest-derived ectomesenchyme. Developmental interactions between these tissue types enable the construction of solid dental structures composed of epithelium-derived crown enamel and ectomesenchyme-derived dentin^1–4^. In humans, teeth primordia are formed in utero and complete their growth before adulthood, at which point the progenitor populations disappear. In contrast to this, in mice and many other species, teeth can continue to grow throughout life, providing the major model system to study progression of various tooth cell lineages from the dental stem-cell populations located in the apical end of the tooth. In mice, the incisor stem-cell population continuously self-renews and replenishes tissues that are lost due to gnawing, making this model attractive for studies of stem-cell generation, cell differentiation, homeostasis, and injury-induced regeneration. In addition, the mouse incisor represents a model of continuously self-renewing organ with cell dynamics conceptually similar to gut epithelium, hair follicles, and nails. Even though major tooth cell types have long been identified, the spectrum of rare and transient cell populations and interactions that enable tooth growth remain poorly understood. The identity of epithelial and mesenchymal stem populations and their possible spatial and functional diversity remains unresolved, especially when it comes to such populations in growing and nongrowing human teeth. Besides, whether rodent teeth represent a bioequivalent model system for studying specific aspects of human tooth development and physiology is not yet clear. The long held-view is that the human teeth contain mesenchymal stem cells analogous to mouse incisor mesenchymal stem cells^5–8^. However, at this point, no clear consensus has been reached about the molecular identity of such cells in vivo^6,9^. In addition, the role and population structure of other cell types, such as resident cells of the immune system, is unclear in relation to the maintenance of local tissue homeostasis and beyond their major protective function in teeth. There is growing evidence that macrophages are important constituents influencing the stem-cell compartments, for instance, in control of the intestinal stem-cell niche or in promoting wound-induced hair follicle regeneration^10,11^.

Towards answering these questions, we applied single-cell transcriptomics and lineage tracing techniques with a specific aim to examine the organizational complexity and self-renewal of growing mouse incisor, contrasting it with nongrowing mouse molars, and evaluating the extent to which the mouse model reflects the growth of human teeth. Our data revealed stem and differentiated cell subtypes in epithelial and mesenchymal compartments and heterogeneity of tissue-residential immune cells in mouse incisor. We provide a comparative map of cell types inhabiting mouse and human growing vs. nongrowing teeth.

## Results

### sc-RNA-seq reveals cell heterogeneity of the self-renewing mouse incisor

**Fig. 1:**
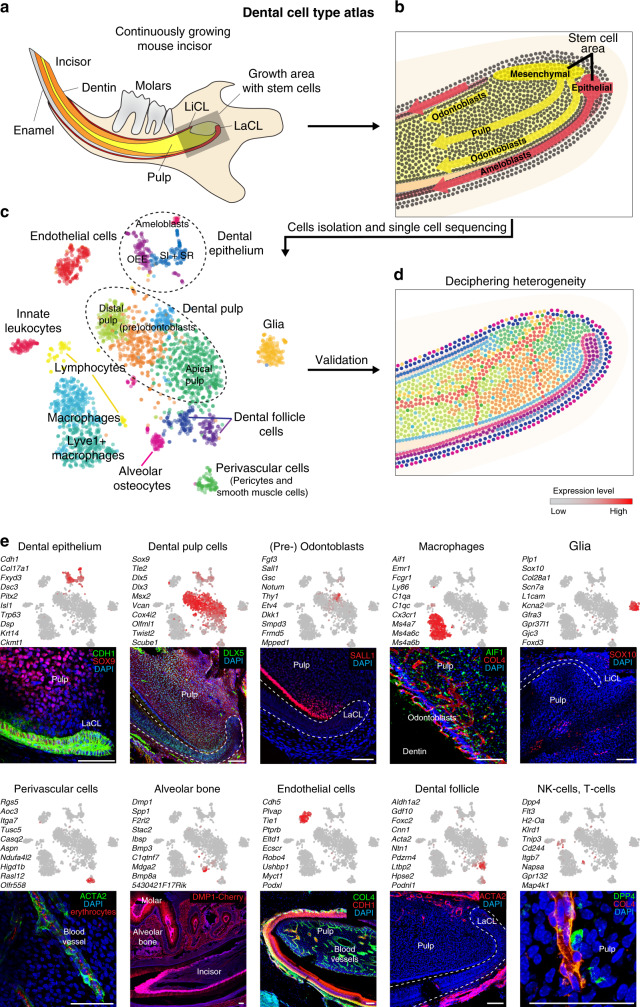
Unbiased identification, validation, and spatial mapping of major dental cell types and subpopulations. **a** Schematic drawing of continuously growing mouse incisor with highlighted stem-cell area. **b** Cell dynamics during self-renewal and growth based on the activity of the dental epithelial and mesenchymal stem cells. **c** Unbiased identification of dental cell types and subpopulations. t-SNE dimensional reduction visualizes the similarity of the expression profiles of 2889 single cells (individual points). Colors demonstrate 17 clusters as defined by PAGODA clustering. All major clusters correspond to cell types in the mouse incisor, defined by expression of known markers. **d** Schematic drawing summarizing validation and mapping of the observed cellular subpopulations back onto the incisor tissue preparations. **e** Validations and mapping of unbiasedly identified populations based on the expression of selected marker genes. All validations were performed by immunohistochemistry except of alveolar bone panel where *DSPP*^*cerulean*^*/DMP1*^*Cherry*^ mice was used (only red channel showed). Note. SOX9 is well-known marker for pulp cells, COL4 for blood vessels, CDH1 for epithelium, and ACTA2 for dental follicle (and perivascular cells). All these marker genes are highly and specifically expressed in corresponding clusters (Supplementary Table 1), but do not belong to top10 genes shown in plots above the images. (LiCL Lingual Cervical Loop, LaCL Labial Cervical Loop, SI Stratum Intermedium, SR Stellate reticulum, OEE Outer Enamel Epithelium). Scare bars: 50 µm.

**Fig. 2:**
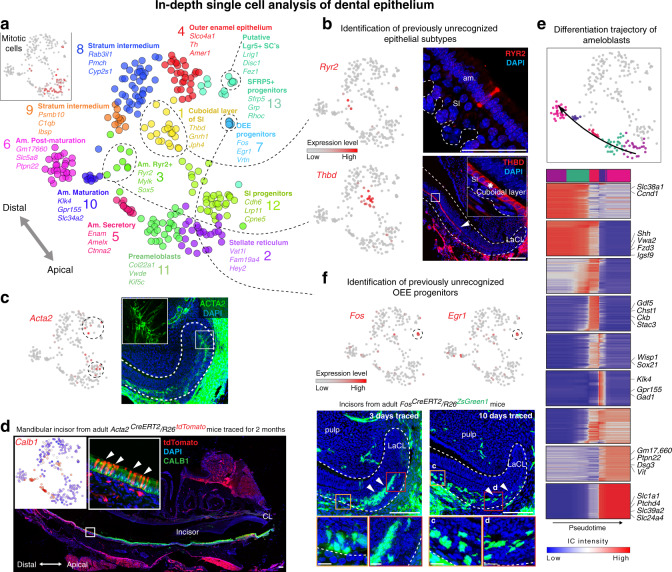
In-depth single-cell analysis of dental epithelium. **a** t-SNE dimensional reduction shows subpopulations of 268 single epithelial cells. 13 unbiased clusters (colors) reveal previously unrecognized stem, progenitor and mature epithelial subtypes. Inset: mitotic signature as defined by average expression of cell-cycle-related genes. **b** Identification of a previously unrecognized cellular subtypes of epithelial layer. RYR2^+^ cells in ameloblasts’ layer and THBD^+^ subpopulation of stratum intermedium organized into cuboidal layer found by immunohistochemistry. **c** Panel on the right shows localization of ACTA2-expressing cells inside the labial cervical loop (immunohistochemistry) and corresponding expression of *Acta2* predicted from RNA-seq analysis (left panel). **d** Long-term (2 months) lineage tracing of a *Acta2*^*CreERT2*^*/R26*^*tdTomato*^ dental epithelial stem cells shows the traced cells in both apical (near the cervical loop) and distal ameloblasts. Ameloblast character was proved both morphologically and by expression of CALB1 (immunohistochemistry). **e** Transcriptional program of ameloblasts differentiation. Four clusters corresponding to different stages of ameloblasts maturation (upper). Transcriptional states of ameloblasts progenitors were modeled as a single trajectory, which reveals sequence of cell state transitions and linked activity developmental gene modules (bottom). Heatmap: the cells (columns) are arranged according to estimated pseudotime, genes (rows) were clustered in nine modules. Smoothed gene expression profiles are shown. **f** Transient progenitor population found in labial cervical loop is demarcated by the expression of *Egr1* and *Fos*. Panels in the bottom part shows the lineage tracing of *Fos*^*CreERT2*^*/R26*^*ZsGreen1*^. Insets show the lineage traced cells in outer enamel epithelium. Of note, *Fos*^*CreERT2*^*/R26*^*ZsGreen1*^ traced cells in epithelial and mesenchymal compartments are of distinct origins since compartments are spatially separated. (LaCL Labial Cervical Loop, SI Stratum Intermedium, Am. Ameloblasts). Scale bars: **b**, **d**, **e**: 50 µm; **c** and insets of **e**: 10 µm.

**Fig. 3:**
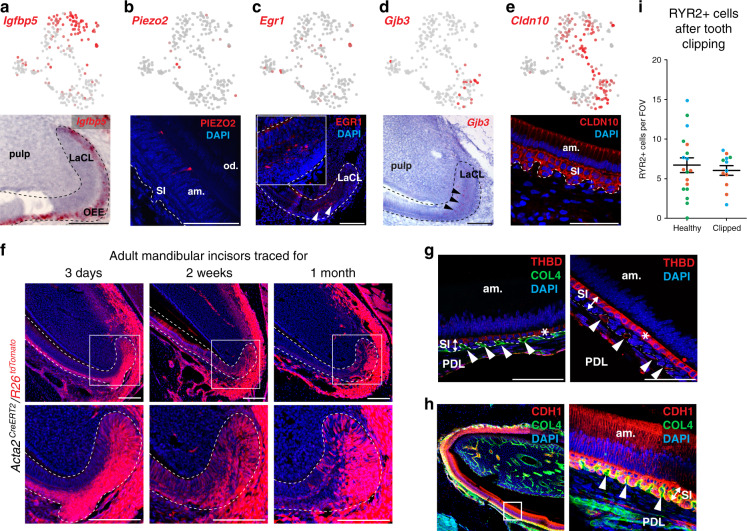
Identification of previously unrecognized cell types in dental epithelium and stem cells. **a**–**e** In situ hybridization (*Igfbp5, Gjb3*) and immunohistochemistry (PIEZO2, EGR1, and CLDN10) validations of selected markers demarcating different progenitor and differentiated states in epithelial layer. Note: Validation of expression of *Igfbp5* enables identification of outer enamel epithelium clusters on a t-SNE representation (**a**). Validation of PIEZO2-expressing cells shows sporadic cells inside the ameloblast layer (**b**). *Egr1*^+^ cells are present in the progenitor area on the edge of stellate reticulum and outer enamel epithelium (**c**). Mapping of *Gjb3* on the section tissue consistently reveals the position in stellate reticulum within the labial cervical loop (**d**). Validation of *Cldn10* expression helps to outline all non-ameloblastic parts of epithelial differentiation including developing stratum intermedium and outer enamel epithelium (**e**). **f** *Acta2*^*CreERT2*^*/R26*^*tdTomato*^ genetic tracing shows significant contribution of *Acta2*^+^ cells of the labial cervical loop to more differentiated cell types of dental epithelium including ameloblasts, stellate reticulum, outer enamel epithelium, and stratum intermedium after 3 days, 2 weeks, and 1 month long tracing period. **g**, **h** Immunohistochemistry identification of the Cuboidal layer of stratum intermedium (expressing THBD) and spatial relation to the neighboring blood vessels submerged into the papillary structure of stratum intermedium. COL4 expression characterizes the blood vessels on left panel. **h** Papillary structure of stratum intermedium with submerged blood vessels (COL4) and CDH1 expressing ameloblasts and cells from stratum intermedium. Note. Cuboidal layer characterized by THBD expression (**g**) forms subpopulation of stratum intermedium cells (**h**). Immunohistochemistry. **i** Comparison of RYR2^+^ ameloblasts in healthy (mean 6.71 ± 0.93 SEM per FOV, Field Of View) and unilaterally clipped (mean 6.04 ± 0.61 SEM per FOV) mouse incisor. Counts of RYR2^+^ ameloblasts per FOV are plotted, and the color-code of dots corresponds to 3 individual animals per healthy or clipped condition. (am. ameloblasts, od. odontoblasts, LaCL Labial Cervical Loop, SI stratum intermedium, OEE Outer Enamel Epithelium, am. Ameloblasts, PDL periodontal ligamentum). Scale bars: 50 µm.

**Fig. 4:**
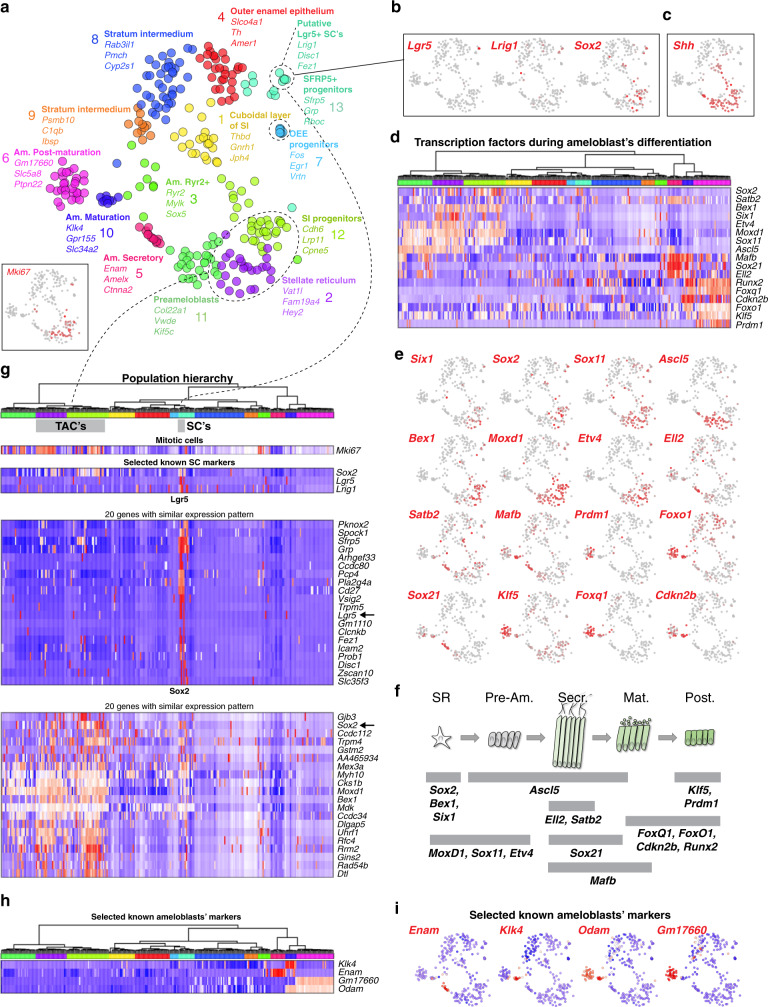
Extended analysis of the heterogeneity of dental epithelial subtypes. **a** t-SNE dimensional reduction visualizes the similarity of the expression profiles of 268 single dental epithelial cells. Thirteen unbiased clusters shown by different colors including revealed stem, progenitor and mature epithelial subtypes. **b** Previously unrecognized identified stem-cell subpopulation shows expression of *Lgr5, Lrig1*, and *Sox2*. Unlike *Lgr5* and *Lrig1*, *Sox2* is more widely expressed also in TAC’s (also shown in panel **g**). **c** *Shh* is expressed in the progenitor populations including the stellate reticulum, stratum intermedium progenitors or preameloblasts (clusters 2, 11, and 12). **d**–**f** Transcriptional factor code associated with ameloblasts differentiation. **f** Schematic drawing summarizing expression of various selected transcription factors in different stages of ameloblasts development. **g** Heatmap showing the expression of mitotic and stem-cell markers within identified clusters of dental epithelial cells. Population hierarchy axis colors resemble the same populations on tSNE from panel **a**. Note that some of previously described stem-cell markers: *Lrig1, Sox2, Bmi1*, *Gli1*, Lgr5, or Igfbp5 are co-expressed only within a subcluster of cluster 13. This subcluster possesses a unique and extensive multigenic signature, including previously unknown markers *Pknox2, Zfp273, Spock1*, and *Pcp4*. The putative DESCs from cluster 13 might represent one type of epithelial stem cells in the tooth. The listed stem-cell markers show reasonably large and partly overlapping domains of expression that coincide with clusters containing proliferating progenitors. *Sox2*^+^ DESCs give rise to *Shh*^+^ populations including transient amplifying cells (TAC’s) in the epithelial compartment. In agreement with that, we observe that the *Sox2*^+^/*Shh*^+^ clusters 12 and 2 contain the majority of TAC’s and most likely represent less differentiated states as compared to *Sox2*^-^/*Shh*^+^ clusters 11, 5, 1, and 13. **h**, **i** Expression of well-known markers corresponding to a different ameloblast stage proving the gradual differentiation from secretory ameloblasts stage (*Enam*^+^) through maturation ameloblast stage (*Klk4*^+^, *Odam*^+^) into postmaturation ameloblast stage (*Gm17660*^+^).

**Fig. 5:**
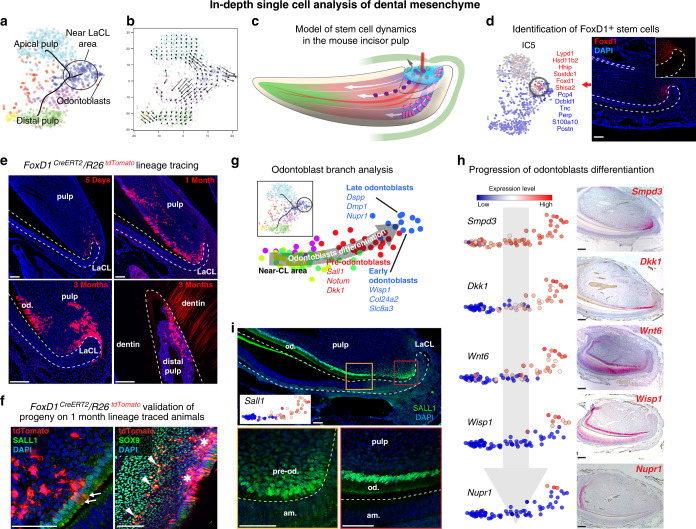
Developmental dynamics of dental mesenchyme. **a** Analysis of mouse-incisor dental mesenchymal cells isolated for separate analysis from general dataset. Colors show unbiased clusters. The principal tree correctly captures positions of mature mesenchymal derivative and progenitor populations. **b** Analysis of RNA velocity shows major directions of cell progression in the transcriptional space. The arrow start- and endpoints indicate current and predicted future cell states. **c** Model of stem-cell dynamics in mesenchymal compartment with relation to dental follicle. **d** Prediction and validation of spatially restricted *Foxd1*^+^ stem cells. The Foxd1-associated axis was selected for validation, and is shown on t-SNE (genes with the strongest positive and negative associations are shown in red and blue respectively). *Foxd1*^+^ cells (in situ hybridization) are located in the mesenchyme surrounding the labial cervical loop. **e** Lineage tracing of *FoxD1*^*CreERT2*^*/R26*^*tdTomato*^ strain confirmed the predicted stem-cell nature of *Foxd1*^+^ mesenchymal cells. In short-term tracing (5days) tdTomato^+^ cells are predominantly around LaCL in contrast to long-term (1-month-long and 3 months) tracing where pulp and odontoblast progeny are observed. Importantly, tdTomato^+^ cells are maintained in their original position in the long-term manner (3 months) and at the same time point tdTomato^+^ distally located odontoblasts can be observed supporting the theory of *Foxd1*^+^ cells being a long-living mesenchymal stem cells. **f** Nature of Foxd1^+^ cells progeny confirmed by a combination of *FoxD1*^*CreERT2*^*/R26*^*tdTomato*^ tracing and SALL1 and SOX9 immunohistochemical stainings. *FoxD1*^*CreERT2*^*/R26*^*tdTomato*^-traced cells contribute to both the SALL1^+^ odontoblasts (arrows) and SOX9^+^ pulp cells (arrowheads). Asterisks show the of subodontoblast layer *FoxD1*^*CreERT2*^*/R26*^*tdTomato*^ traced cells. **g** Variability of cells assigned to a branch leading to odontoblasts (inset) was reanalysed using principal component analysis. Colors mark five clusters obtained by unbiased hierarchical clustering. Left-right axis reflects developmental stages of odontoblasts. **h** Gradual odontoblast differentiation (suggested in **g**) from near-CL area into fully differentiated odontoblasts. Left: expression pattern acquired from scRNA-seq, right: in situ hybridization-based histological validations of the proximal part of the mouse incisor proving suggested gradual transition. **i** Spatial pattern of a discovered (pre)odontoblast transcription factor—SALL1 (Immunohistochemistry). (LaCL Labial Cervical Loop, pre-od. preodontoblasts, Od. Odontoblasts, Am. Ameloblasts). Scare bars: 50 µm.

**Fig. 6:**
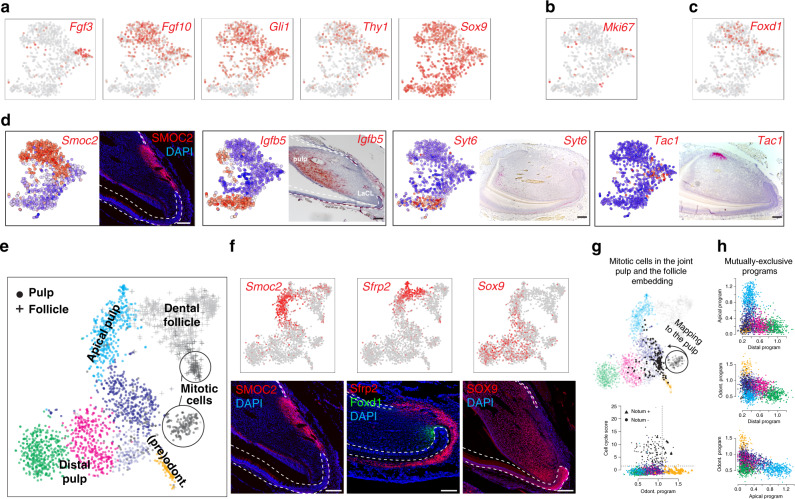
Portrait of transcriptional heterogeneity in dental mesenchymal populations. **a** t-SNE representations of selected, previously known marker genes. **b** t-SNE representation showing position of *Mki67*^+^ cells. **c** t-SNE representation showing position of *Foxd1*^+^ cells. **d** Immunohistochemistry (SMOC2) and in situ hybridization (*Igfbp5, Syt6,* and *Tac1*) characterization of key populations in the mesenchymal population. Importantly, Igfbp5 and Syt6 demarcate more distal pulp and Tac1 is a unique marker for dental pulp attached to the lingual cervical loop. **e** Landscape of mesenchymal cells of dental pulp (dots) and follicle (crosses) is reproduced and extended with 10× Chromium. t-SNE embedding shows 2552 mesenchymal cells grouped in clusters, where clusters colours reflect colours of annotated Smart-seq2 pulp clusters (see Fig. 3a). Of note, cells from apical pulp gradually extend into cells of dental follicle. **f** Experimental validations of gradual spatio-transcriptional gradient from apical pulp to dental follicle. Immunohistochemistry of SMOC2 highlights the position of the apical pulp state, corroborated by Smart-seq2 and 10× Chromium single-cell datasets. In situ hybridization of *Sfrp2*, consistent with 10x Chromium and Smart-seq2 t-SNE representations, labels coherent states between apical pulp and dental follicle. Immunohistochemistry for SOX9 labels all pulp cells but not odontoblasts or dental follicle. **g** In silico mapping of mitotic cells onto non-mitotic landscape pinpoints progenitor states of active cell division (upper). To remove effect of mitotic program, mitotic cells were re-positioned as average of 10 transcriptionally similar non-mitotic cells. Comparison of intensity of odontoblast (lower, *X*-axis) and cell cycle (lower, *Y-*axis) programs in each cell reveals a subset of mitotic cells with activated odontoblast program (lower). Dashed lines demarcate cells with active programs. **h** Mutually exclusive activation of fate-specific programs (odontoblasts, distal and apical fates). An estimate of activity of fate-specific programs in each cell was based on average expression of 20 fate-specific markers. Comparison of pairs of fates (three panels of fate pairs) shows activation of only one of fates indicating lack of noticeable multilineage priming.

**Fig. 7:**
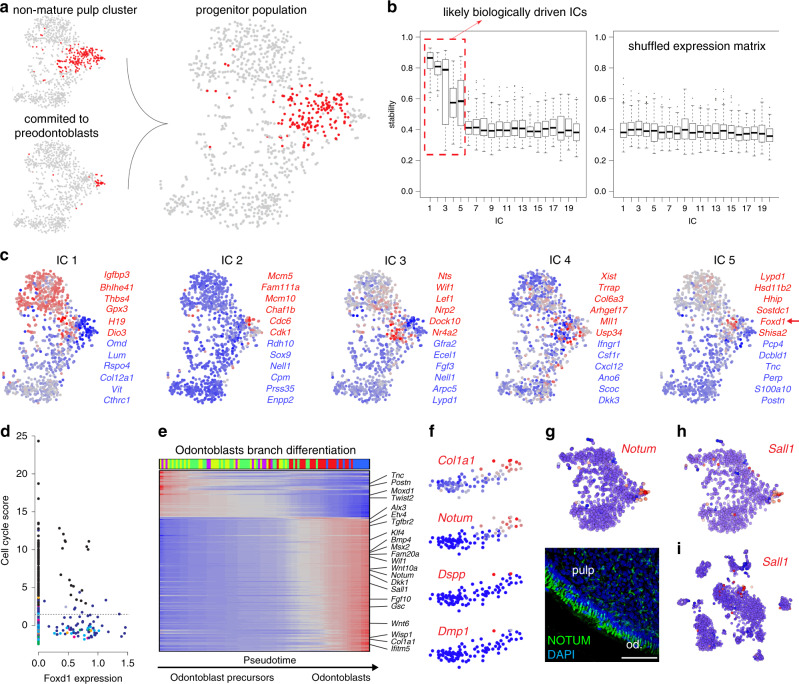
Detailed analysis of mesenchymal branching point and odontoblast lineage. **a** Selection of non-mature subpopulation. An unbiased cluster of cells that do not represent mature pulp populations was selected (left, upper), and preodontoblasts (left, lower), were excluded from it, resulting in non-mature subpopulation (right). **b** Analysis of ICs stability reveals five biologically driven aspects of heterogeneity of non-mature subpopulation. Average correlation of ICs to the most similar ICs across 100 runs of subsamplings of 70% of cells (right) reveals 5 out of 20 ICs with stability substantially higher than expected from shuffled control (left). Stability of all ICs of control shuffled matrix and 15 ICs of original matrix are around 0.4 indicating background expectations of spurious components. **c** Five ICs of non-mature subpopulation reveal processes related to (from left to right) apical gradient (IC 1), cell cycle (IC 2), *Fgf3*-mediated (IC 3), and *Foxd1*-mediated (IC 5) heterogeneity restricted to the progenitor states. Colors show intensities of identified ICs. Genes that have the highest (red) and lowest (blue) associations with corresponding IC are shown. **d** Graph showing cells expressing *Foxd1* in comparison to cell-cycle score. **e** Transcriptional events during pulp differentiation trajectories. Each trajectory encompasses cell states from progenitor, as identified from analysis of preodontoblasts, to mature states with cells arranged by pseudotime reflecting maturation process. The black cells belong to the trajectory being shown, while other cells are shown in light gray (upper panels). Heatmap shows smoothed gene expression profiles with cells arranged by pseudotime and genes (rows) arranged by pseudotime of maximal expression (lower panels). **f**–**i** Expression analysis of the selected genes determining odontoblasts (*Col1a1, Dmp1,* and *Dspp*) and together with previously unrecognized identified odontoblast marker genes (*Notum* and *Sall1*). Notum expression is visualized on t-SNE embedding of the pulp dataset and immunohistochemistry proves NOTUM to be expressed in odontoblasts (**g**). *Sall1* expression is visualized on t-SNE embeddings of the both pulp (**h**) and complete incisor dataset (**i**). Scale bars: 50 µm.

**Fig. 8:**
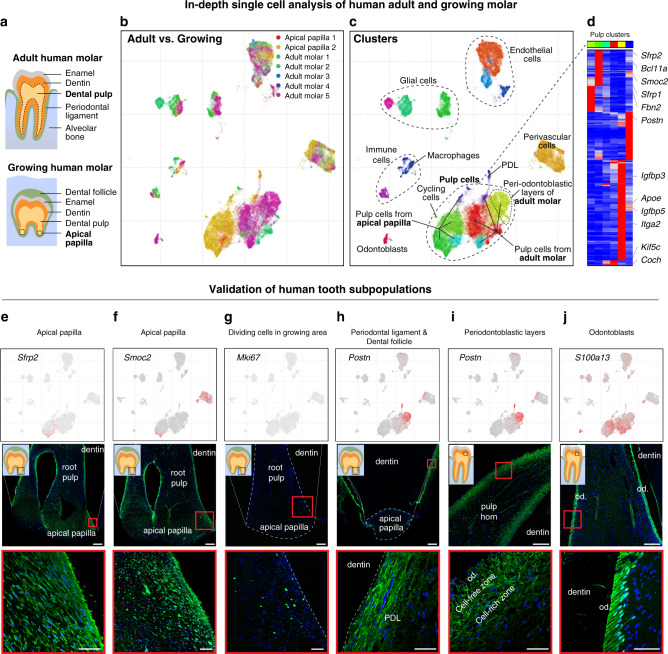
Single-cell analysis of human adult and growing teeth. **a** Scheme of pulp regions isolated for single-cell RNA-seq from adult human molars and apical papillae of growing human molars (dotted regions). **b** Characterization of cell composition across five adult and two growing human molars using scVI deep learning framework. UMAP dimensionality reduction visualizes similarity of expression profiles of 39,095 single cells. Colors correspond to individual datasets and indicate clustering by cell types. **c** Characterization of dental cell types in human teeth. Colors demonstrate 17 clusters as defined by leiden clustering. Major clusters are defined by expression of known markers. **d** Human dental pulp have at least six transcriptionally distinct states. Top color bar reflects colors of clusters shown in **c**). Top 198 genes enriched in each cluster are shown (maximum to medium expression across clusters is at least four-fold and *p* value < 10^−50^, *one-way* ANOVA test). **e**, **f** Identification of apical-like-mouse-incisor regions in the growing apical papilla of human molar shown by the expression of SFRP2 and SMOC2 (immunohistochemistry) in the growing region of apical papilla. **g** Dividing, MKI67^+^ cells are positioned in the growing part of the apical papilla. **h**, **i** Expression of *POSTN* shows very regionalized pattern in two main clusters: periodontal ligament (PDL) on the samples from apical papillae (**h**), but also demarcate the periodontal layers of adult dental pulp previously recognized as a cell-rich and cell-free zones (**i**). Immunohistochemical POSTN staining. **j** *S100A13* was proposed as a marker of human odontoblasts. This gene is highly overexpressed in one of the subclusters, which is on t-SNE located in the close proximity to dental pulp. S100A13 was proved to be expressed in odontoblasts by immunohistochemistry. (Od. Odontoblasts; PDL periodontal ligament). Scale bars: 50 µm, insets: 250 µm.

**Fig. 9:**
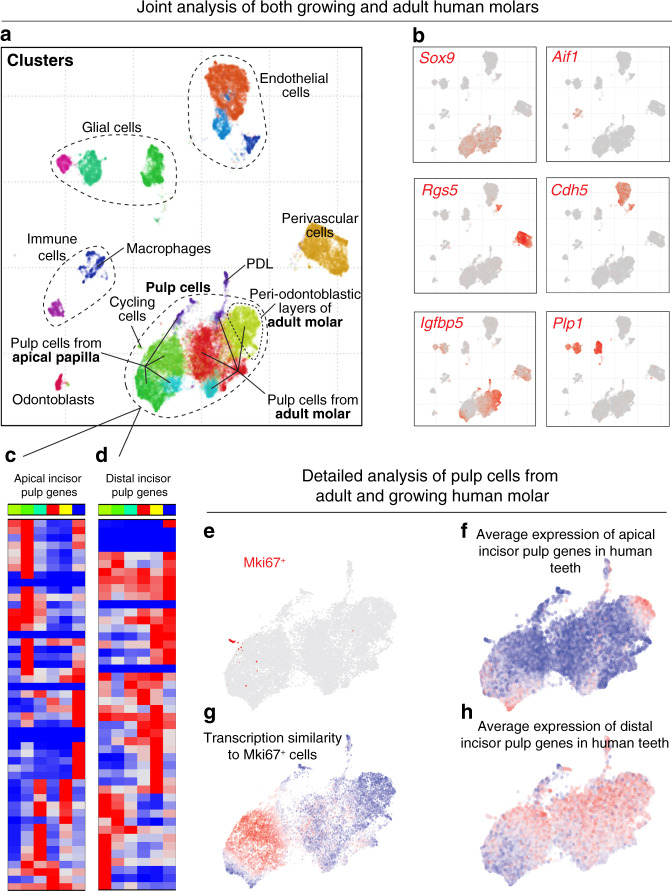
Analysis of adult and growing human molars. **a** Dental cell types in human teeth, see Fig. 4. **b** Expression of selected marker genes. **c**, **d** Expression of genes coordinately active in apical (51 genes, **c**) or distal (48 genes, **d**) incisor pulp across clusters of human mesenchyme reveals divergence of pulp expression programs. Apical incisor genes were defined as at least three-fold and significantly (*p* < 10^−10^, two-sided *t*-test) overexpressed in apical compared to distal incisor pulp in both 10× Chromium and Smart-seq2 datasets. The same for distal incisor genes. **e** Expression of *MKI67* in cells of human pulp shows a group dividing cells. **f** Transcriptional similarity of the group of dividing cells to individual nondividing mesenchyme cells. **g**, **h** Average expression of apical incisor genes (**g**) and distal incisor genes (**h**) in cells of human mesenchyme outline tendency to expression in complementary cell states.

**Fig. 10:**
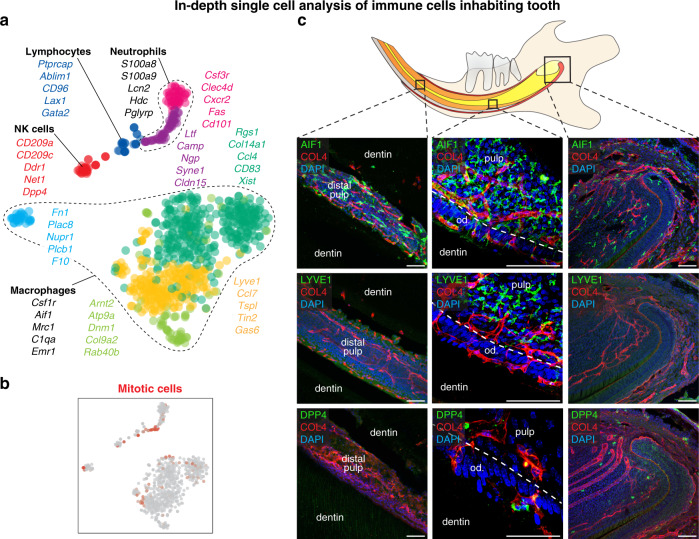
Heterogeneity of immune cells in mouse incisor. **a** t-SNE dimensional reduction shows ten identified populations of immune cells. **b** Position of mitotic cells in the immune cluster. **c** Location of tissue-residential immune cells in the different parts of mouse incisor. AIF1^+^ macrophages are located in the whole incisor including apical pulp, cervical loop, odontoblast layer, and distal pulp in contrast to LYVE^+^ macrophages which mostly resides in the middle part of the pulp, but not inside the odontoblast layer. DPP4^+^ immune cells are sporadically located in the apical part of the tooth and odontoblast layer. COL4 immunohistochemical staining visualize the blood vessels. (Od. Odontoblasts), Scale bars: 50 µm.

To address the entire course of differentiation of cell types in the tooth during self-renewal, we first isolated all dental tissues from the adult mouse incisor and sequenced individual cell transcriptomes with the Smart-seq2 protocol to obtain high sequencing depth^12^ (Fig. 1a, b). Clustering using PAGODA revealed 17 major cell subpopulations (Fig. 1c–e; Supplementary Figs. 1–3 and Supplementary table 1,2,3 and Supplementary Data File 1), including the major immune, epithelial, and mesenchymal compartments. The relative in vivo cell-type abundances might not be reflective of clusters proportions due to cell isolation biases and strategies^13^. All general cell types show considerable degree of internal heterogeneity (Supplementary Figs. 1e–g, i, 3) emphasizing complexity of interactions and physiological processes in a growing and self-renewing tooth. We next focused on the most striking aspects of population complexity of epithelial (Figs. 2–4) and mesenchymal (Figs. 5–7) compartments, their human analogues (Figs. 8, 9), and finally immune (Fig. 10) populations.

### Heterogeneity of the epithelial compartment in mouse incisor

The epithelial compartment of the tooth is essential to generate the enamel, as well as for the morphogenetic guidance of tooth development and self-renewal. Focused reanalysis of the epithelial (*Krt14* and *Cdh1* co-expressing) subpopulation showed a complex mixture of at least 13 distinct epithelial clusters (Fig. 2a). These include mature subpopulations, such as enamel-generating ameloblasts, and a heterogeneous pool of stem/progenitor cells. During sequencing, we enriched for epithelial progenitors by using *Sox2*-driven GFP in *Sox2*^*GFP*^ animals and subsequent FACS of fluorescent cells. Ameloblasts and enamel development in the incisor is not restricted to early developmental stages as it is in molars. Continuous replenishing of enamel is essential for the incisor growth. Thus, we can find all the ameloblasts’ stages: closer to the labial cervical loop we can find early stages and closer to the tip more differentiated stages. Differentiation of ameloblasts starts at the preameloblasts stage (early fate decision and first differentiation), then continues through secretory stage during which the enamel backbone is formed. Subsequently, the secretory ameloblasts are further differentiated in a maturation phase during which the first enamel backbone is fully calcified^2^. During the last phase, known as a postmaturation phase, the enamel epithelium diminishes and enamel production is completed. Consistent with these stages, we observed spatially separated stages of ameloblast differentiation, including preameloblasts (*Shh*^+^ cluster 11), secretory (*Enam*^+^ cluster 5), maturation (*Klk4*^+^ cluster 10) and postmaturation (*Gm17660*^+^ cluster 6) stages (Fig. 2a, e, Fig. 4a, c, h, i)^14–16^. Our results show that transitions in gene expression profiles between the canonical stages are rather abrupt, consistent with the fact that the stages were previously characterized based on significant morphological and functional changes during ameloblast differentiation. The data show progressive modulation of transcription factor expression during different stages of ameloblast development (Fig. 4d–f), connecting known spatial and morphological transitions associated with the ameloblast differentiation with previously uncharacterized intermediate transcriptional states. In addition to these spatially separated populations, we observed a subset of RYR2^+^ cells scattered in the ameloblasts layer (cluster 3) (Fig. 2a, b). The function of these cells is unknown, however this population expresses different mechanotransduction-related genes: *Piezo2*, *Trpm2, Trpm3*, and *Trpm6* cation channels, as well as calcium-dependent genes (*Itpr1, Ryr1,* and *Ryr2*) (Fig. 3b and Supplementary Table 1)^17–19^. To clarify if these cells can respond by changing their numbers to the lack of mechanical load, we clipped the incisor on one side of the jaw to prevent the usage of this tooth for a significant period of time. This unilateral tooth clipping experiment did not reveal any changes in the number and distribution of RYR2^+^ cells (Fig. 3i).

Other mature populations in the tooth epithelium included stratum intermedium (clusters 8, 9, and 1) and outer enamel epithelium (cluster 4), whose functions are poorly understood (Fig. 2a). The identity of cluster 1 was unclear, but immunohistochemistry using THBD as a marker specific to this population, revealed that these previously uncharacterized cells reside in a distinct anatomical structure that we named the cuboidal layer of stratum intermedium (Figs. 2b, 3g, h). The broader gene expression signature of these cells (*Cygb, Nphs1,* and *Rhcg*) suggested that they maintain the functional interphase between blood vessels and metabolically active ameloblasts^20–23^. Such function might be important for proper ameloblasts’ activity essential for the efficient enamel synthesis.

The repertoire of stem and progenitor cells supporting these diverse tooth epithelial populations is poorly characterized. A combination of putative markers for dental epithelial stem cells (*Sox2, Lrig1, Bmi1, Gli1, Igfbp5,* and *Lgr5*) identified from studies of late embryogenesis^24–28^, showed most consistent expression in a subset of cluster 13 (Figs. 2a, 4b, c, g). Even this small subpopulation, however, was heterogeneous. For instance, *Sox2*, *Acta2* and many other genes were specifically co-expressed in a single cell from the stem-cell subcluster, suggesting a distinct stem-cell subtype. Indeed, lineage tracing with *Acta2*^*CreERT2*^*/R26*^*tdTomato*^ mice traced ameloblasts and other cell types of dental epithelium in adult animals after 3 days, 2 weeks, 1 month and 2 months after tamoxifen injection (Figs. 2d and 3f). The presence of ACTA2^+^ cells was confirmed by immunohistochemistry (Fig. 2c) in the outer half of stellate reticulum, outer enamel epithelium and dental follicle. Lineage tracing data using the *Acta2*^*CreERT2*^*/R26*^*tdTomato*^ mice appeared consistent with this immunohistochemical staining. In the short-term lineage tracing experiment (3 days), numerous cells appeared traced within dental epithelium. However, the mature ameloblasts were not traced, which is different from long-term lineage tracings (1−2 months), where mature ameloblasts are robustly detected (Figs. 2d, 3f). At the same time, the overall numbers of all traced cells decreases over time because these cells are being replaced by the progeny of non-labeled stem cells. Only a small fraction representing traced epithelial cells is derived from ACTA2^+^ epithelial stem cells, which retain self-renewing capacity and can produce a minor proportion of epithelial progeny constantly during incisor self-renewal.

The expression patterns of the epithelial stem-cell markers show partial overlap with diverse clusters of proliferating progenitors. These include *Shh*^+^ cells^25,29^ (*Sox2*^+^/*Shh*^+^ clusters 12 and 2, as well as more differentiated *Sox2*^−^/*Shh*^+^ clusters 11, 5, 1, 13; Figs. 2a, 4c). Expression of *Egr1* and *Fos* in cluster 7 suggested a distinct type of an epithelial progenitor. Immunohistochemistry labelling showed that *Egr1*^+^ epithelial cells were positioned adjacent to the stem-cell niche (Figs. 2f, 3c). Lineage tracing in *Fos*^*CreERT2*^*/R26*^*ZsGreen1*^ mice revealed epithelial progeny inside the cervical loop, and predominantly in the outer enamel epithelium 10-days after the induction of lineage tracing in *Fos*^*CreERT2*^*/R26*^*ZsGreen1*^ mice (Fig. 2f). These *Egr1*^+^/*Fos*^+^ cells, thus, although being *Sox2* negative, represent a long-lasting progenitors that disappear after 1 month of the lineage tracing from the cervical loop, and that are fate-biased towards outer enamel epithelium. Overall, our analysis of the epithelial compartment revealed a complexity of stem, progenitor and mature cell types, many of which were previously unknown and provide opportunities for further characterization.

### Heterogeneity of the mesenchymal compartment in mouse incisor

Mesenchymal cell types in teeth build cementum, dentin, and soft tissue of pulpal cavity, and have diverse spatial localizations inside and around the tooth. Our data revealed that the tooth is surrounded by two subtypes of the dental follicular cells and is encapsulated by the alveolar bone (Fig. 6e–h, Supplementary Fig. 3a, and Supplementary Table 1)^27,30,31^. The dental follicle populations express *Aldh1a2 -* the key enzyme for retinoic acid production (Supplementary Fig. 3a, b). Retinoic acid, being a key morphogen, is known to control dental development and self-renewal^32,33^. Correspondingly, complementary receptor genes *Rara, Rarb*, and *Rarg* are expressed in some of the major populations of the tooth itself (Supplementary Fig. 3a, b). This suggests previously unanticipated crosstalk between retinoic acid producing and sensing populations in incisor growth and maintenance.

Inside, the incisor contains a continuously replenished mesenchymal compartment, comprised of odontoblasts producing dentin (the most abundant type of hard matrix in teeth), and heterogeneous sets of pulpal cells whose role and subtypes remain to be understood from the functional point of view. Smart-seq2 data showed at least three major mesenchymal populations inside of the mouse incisor: odontoblasts and two distinct pulp subtypes, all connected by a continuum of transient cell states (Fig. 5a, b). The first pulp subtype, constitutively expressing *Smoc2* and *Sfrp2*, is localized specifically to the apical pulp in the area between cervical loops according to validation experiments (Fig. 6d, f). Expression of genes linked to self-renewal properties in the incisor mesenchyme (*Thy1* and *Gli1*) was restricted to cells of apical subtype (Fig. 6a)^27,34^. However, dividing cells (*Mki67*^+^) are mostly segregated to a distinct heterogeneous transcriptional subpopulation localized in the pulp near the cervical loops, as evident from *Fgf3* and *Foxd1* expression (Fig. 6a–c). This indicates that apical pulp subtypes include diverse pools of quiescent stem cells and stromal cells likely supporting the stem-cell niche. The other pulp subtype corresponded to incrementally differentiating distal pulp cells finally labelled by the expression of *Igfbp5* and *Syt6* (Fig. 6d). Transcriptional trajectory modelling of the three mesenchymal populations, predicted a central branchpoint at a subpopulation with a strong mitotic signature, suggesting a likely active pool of stem/progenitor cells within the mesenchymal compartment (Figs. 5a–c, 6g)^27,35^. The potential area of active progenitors was corroborated by RNA velocity (Fig. 5b)^36^.

To improve the resolution of the active stem/progenitor subpopulation, we profiled mouse incisor by sequencing a larger number of cells using the 10× Chromium platform, which recovered the same mesenchymal landscape and overall population structure (Supplementary Fig. 4e, h). Branch analysis showed that transcriptional programs of the three populations were activated in a mutually exclusive manner in individual cells, without a notable multilineage primed state (Fig. 6h)^37^. A small fraction of the dividing cells showed activation of population-specific transcriptional biases, including an odontoblastic program. Obtaining cells of odontoblast sublineage became possible because we enriched for it by using *Dspp*^*cerulean*^*/Dmp1*^*Cherry*^ transgenic animals^38^. The immunohistochemistry confirmed activation of early odontoblast markers *Notum* and *Sall1* in the near cervical loop mesenchymal area, indicating that odontoblast fate selection happens before embedding into the odontoblastic layer (Figs. 5g, i.7g). However, it is not clear if all *Notum-* and *Sall1-*expressing progenitor cells always irreversibly and selectively commit to the odontoblast fate or these factors convey a strong bias towards odontoblast differentiation. This goes in-line with the previously established fact that proximity of a stem cell to the epithelial compartment was shown to modulate selection of odontoblast fate, indicating the extrinsic signal from epithelium might induce odontoblast program^39^. This initial fate selection step, as well as clear transcriptional progression through at least three spatially separated stages of odontoblast differentiation provide a useful resource for ongoing efforts for targeted differentiation of odontoblasts (Fig. 5g, 7e–i).

Analysis of the apical progenitor subpopulation demarcated several axes of transcriptional heterogeneity that could identify programs specific to progenitor pools, one of which is marked by *Foxd1* expression (Figs. 5d, 7a–c). In situ hybridization confirmed the expression of *Foxd1* exclusively near the labial cervical loop area (Fig. 5d). A fraction of these cells is mitotic (Fig. 7d). To test whether *Foxd1* expression designates a functionally distinct subpopulation of biased stem cells residing in apical area, we performed lineage tracing using *Foxd1*^*CreERT2*^*/R26*^*tdTomato*^. Indeed, we found that *Foxd1*-traced cells gave rise predominantly to periodontoblastic pulp cells and dentin-secreting odontoblasts (Fig. 5e, f). Even after 3-months-long tracing, *Foxd1*-traced cells in the apical stem-cell area were detected only near the labial cervical loop revealing a spatially restricted structure of self-renewal pathway in the mouse incisor (Fig. 5e). Thus, the initial position of stem cells along the central-periodontoblastic axis is associated with its transcriptional state, migratory trajectory, and fates of progeny (Fig. 5c).

### Comparisons of composition of growing vs. nongrowing mouse teeth

Although the mouse incisor stands as a model for a growing tooth, molecular features that distinguish it from nongrowing teeth remain unexplored. Therefore, we generated single-cell transcriptional snapshots of a nongrowing adult mouse molar using both 10X Chromium and Smart-seq2 platforms. To leverage total scale of multiple datasets, we analysed them jointly and together with self-renewing incisor datasets using Conos data integration strategy (Supplementary Fig. 4)^40^. Coarse-grained cell-type composition appeared similar between molar and incisor, except for the lack of epithelial populations in adult molars (Supplementary Fig. 4a–c). However, molar pulp appeared significantly more homogeneous as compared to the pulpal populations of the incisor given the resolution of the current measurements. Joint Conos clustering of incisor and molar datasets shows that molar mesenchyme falls into a single cluster shared with the distal mouse-incisor pulp (Supplementary Fig. 4a, b). Analysis of mesenchyme heterogeneity using separately 10× and Smart-seq2 platforms corroborated the heterogeneous population structure of mouse incisor and homogeneous distal-like population of mouse molar (Supplementary Fig. 4d, e). At the same time, gene expression programs of mouse molar and distal incisor pulp have noticeable expression differences in 379 genes (*p* value < 10^−2^, *t*-test group means comparison and at least two fold change in both Smart-seq2 and 10× Chromium datasets) (Supplementary Fig. 4f). Mouse-incisor apical genes tend to show high expression in a *Smoc2*^*+*^ compared to *Smoc2*^*−*^ human apical papilla. On the other hand, mouse-incisor distal genes tend to show high expression in a *Smoc2*^*−*^ and not *Smoc2*^*+*^ human apical papilla. In adult teeth, mouse-incisor distal genes are uniformly expressed in all populations, but incisor apical genes show the affinity to the periodontoblastic pulp. The meaning of this heterogeneity is unknown and requires further investigation. Altogether, these results support aetiology of the apical subtype in the incisor as stromal and quiescent cells of the niche, the structure absent in the nongrowing molar. We thus, suggest that the distal-like subtype is a constitutive terminally differentiated population, while the apical pulp state is an emergent property of growing mesenchymal dental tissue. Importantly, the apical incisor pulp shows a coherent expression of genes involved in regenerative response in a tooth and production of a hard matrix in case of physical damage (*Sfrp2, Lef1, Fzd1, Sfrp1, Rspo1, Trabd2b, Gli1,* and *Wif1*)^41^, which is much less present in the pulp populations found in molars (Supplementary Fig. 4d, e).

### Parallels and differences between growing and nongrowing human teeth

The studies of mouse incisor are generally motivated by the translational insights on human tooth development. In humans, the growth of teeth stops postnatally after permanent teeth erupt between 6–21 years of age (eruption of the 3rd molar is variable). To determine the extent to which the observed pulp contrast between growing and nongrowing teeth in mouse reflects human biology, we conducted single-cell profiling of 39,095 cells from healthy nongrowing and growing wisdom teeth in humans (Figs. 8, 9). To focus on the growth-relevant populations, the cells were isolated from the apical papilla located in most apical part of developing wisdom tooth where the tooth is still growing. The analysis revealed that human teeth contain cell types analogous to those in mice, including vascular and perivascular cells, glia and immune populations, and distinct subpopulations of pulp cells (Figs. 8a–d, 9a, b).

Human pulp cells significantly differ between the growing apical papilla and nongrowing molar, and form at least several transcriptionally distinct subpopulations (Fig. 8c, d). In that regard, the pulp of human nongrowing molars appeared to be much more transcriptionally diverse compared to the mouse nongrowing molars. In particular, human molar contained a pulp subpopulation that was spatially localized in the periodontoblastic layer, previously morphologically described as cell-free and cell-rich zones, which are absent in mouse (Fig. 8i)^42^. We detected a group of proliferative cells in a growing human apical papilla, which showed pronounced transcriptional similarity to a *Smoc2*^−^ human apical papilla pulp, and dissimilarity with any subpopulations of nongrowing human molars (Figs. 8e–g, 9e, g). To explore similarity of genetic programs in a mesenchymal compartment of human and mouse teeth, we compiled a set of marker genes that are differentially expressed between apical and distal mouse-incisor subtypes in both Chromium 10x and Smart-seq2 datasets (Supplementary Table 4). Assessment of average expression of marker genes of apical and distal incisor pulp subtypes showed their preferential expression in corresponding populations of human pulp cells (Fig. 9f, h). In particular, similar to incisor apical pulp, *Smoc2*^+^ human pulp cluster tends to express apical incisor markers and repress distal incisor markers. Immunostaining reveals localization of *Smoc2*^+^ human subtype to mesenchymal regions demarcating apical papilla around the Hertwig epithelial root sheath (Fig. 8e, f). Overall, these data indicate that *Smoc2*^−^ and *Smoc2*^+^ human pulp subtypes might form a maturation hierarchy similar to that in mouse incisor. However, individual genes inside both apical and distal incisor pulp modules often have incoherent patterns across human pulp subtypes (Fig. 9c, d). This suggests an evolutionary divergence between mouse and human gene expression programs governing development and homeostasis of dental pulp tissue (Fig. 9c, d) and precludes establishing the homologous fine subtypes between mouse and human pulp. Thus, some human pulp subpopulations do not appear to have clear parallel in mouse teeth.

### Heterogeneity of tissue-residential immune cells in mouse incisor

Immune cells are the first responders to any infection invading the pulp cavity^43^. Understanding the organization and diversity of the dental immune system can help develop approaches to improve dental treatments to preserve dental pulp and odontoblasts. We observed eight well-defined immune cell populations in the mouse incisor, dominated by an extensive repertoire of macrophages and other innate immune cells including intravascular and tissue-resident DPP4^+^ natural killer (NK) cells (Fig. 10a, c; Supplementary Fig. 5a, b, e and Supplementary Table 1).

The population of macrophages and dendritic cells contained three subclusters (Fig. 10a). The most evident was presence of *Aif1*^*+*^*/Lyve1*^*+*^ and *Aif1*^*+*^*/Lyve1*^*−*^ populations (Fig. 10, Supplementary Fig. 5a, b and Supplementary Table 1) Unexpectedly, immunohistochemistry demonstrated regional specificity of LYVE1^+^ and LYVE1^−^ macrophage subpopulations: while LYVE1^+^ macrophages resided in the pulp distant from odontoblast layers, LYVE^−^ macrophages were scattered ubiquitously and penetrated the odontoblast layer (Fig. 10c, Supplementary Fig. 5c, d). Given the importance of the tooth immune system in preventing caries, we tested whether similar patterns are also present in human teeth. Indeed, examination of analogous macrophage populations in the human dentition confirmed regional specificity of the LYVE1^+^ population across species (Supplementary Fig. 5f). Interestingly, the density of macrophages in an intact mouse unerupted incisor was much higher than in the surrounding tissues (Supplementary Fig. 5c) and this tooth shows the same patterns as fully developed adult incisor in presence of of *Aif1*^*+*^*/Lyve1*^*+*^ and *Aif1*^*+*^*/Lyve1*^*−*^ macrophages populations.

## Discussion

Coordination of mesenchymal and epithelial compartments is a common feature of self-renewing and developing tissues and organs. Continuously growing mouse incisor has been widely used as a model of tooth development as well as a model of self-renewing organ in general^44,45^. Earlier studies, using bulk RNA-seq, have elucidated some of the transcriptional complexity, characterizing differentiated and progenitor cells in stem-cell niches^27^. Our results based on a single-cell transcriptomics go further to reveal previously unappreciated complexity of the terminal and transient cell states that altogether enable self-renewal and growth of mammalian teeth.

In addition to the previous lineage tracing studies reveling the nature of the *Sox2*^*+*^, *Bmi1*^*+*^, and *Lrig1*^*+*^ dental epithelial stem cells^24,25,27^, we identified stem population of *Acta2*^*+*^ cells in the labial cervical loop. The lineage tracing experiments presented here or published by other authors never showed the entire population of ameloblasts to be traced. Instead, the epithelial progeny appears in characteristic patches, supporting the diversity of epithelial stem cells. Furthermore, we identified *Egr1*^+^ long-lasting epithelial progenitors, which appeared to be similar to a concept of short-living stem cells. Thus, the progenitor area might rely on functional diversification of different stem cells with a stemness gradation. Such diversity of epithelial progenitor cell subtypes might also reflect the remarkable plasticity noted by earlier studies^35,46^.

Aside from the discovered epithelial stem-cell types, our unbiased scRNA-seq approach uncovered different subtypes within incisor epithelium including the subtypes of stellate reticulum, stratum intermedium or a population of ameloblasts that expresses some mechanotransduction-related genes. Future research is required to clarify a precise role of this population. Although the functional and histological structure of mouse-incisor enamel organ was previously extensively investigated^25,27,46^, we introduced a sublayer of stratum intermedium—cuboidal layer, which is positioned immediately underneath the ameloblast layer. The in-depth characterization of a transition from progenitors to mature ameloblasts may benefit ongoing attempts to establish a system of ameloblast differentiation in vitro or to grow dental organoids.

Although our Smart-seq2-based analysis provided a sequencing depth allowing to find populations with fine transcriptional differences, it is laborious and expensive, which precludes the analysis of large cell numbers. Complementary to our Smart-seq2-based study of the epithelial compartment, Sharir and co-authors addressed heterogeneity and the plasticity of the incisor epithelium at a single-cell level^46^. In addition to major epithelial groups, also described in their single-cell study, we identified a number of small subpopulations with finer transcriptional differences, including cuboidal epithelial layer, *Ryr2*^*+*^ population and subtypes of stem or progenitor cells (*Acta2*^*+*^*, Egr1*^*+*^). Sharir et al. demonstrated the capacity of Notch1-expressing cells to convert into ameloblasts upon injury, which significantly extends our results in a domain of dental regenerative response. Compared to other single-cell studies, we characterized also heterogeneity of all cellular subtypes of mouse and human teeth.

Although several dental mesenchymal stem-cell markers had been previously presented^27,34,39,47,48^, in all cases they are not specific to mark the stem cells only as they are expressed in wider population extending in the area between the cervical loops or along the neurovascular bundle. Here we present a spatially completely segregated and specific subtype of multipotent long-lasting *Foxd1*^+^ mesenchymal stem cells attached to the labial cervical loop. These stem cells contribute to odontoblasts, subodontoblastic subtype of pulp cells and other populations of dental pulp. We identified this population based on the in-depth scRNA-seq analysis and proved their functionality by the lineage tracing. Next to a stem-cell niche, odontoblast and pulp fates demonstrated unexpectedly fast separation occurring in a spatially restricted manner, which suggests cell–cell interaction between odontoblast fate-inclining cells with the epithelial layer (Supplementary Fig. 4). We further run a separate analysis of pulp branch and subsequently odontoblast branch only. By doing this we were able to map a complete differentiation pathway of odontoblasts differentiation which was subsequently proved by in situ hybridization. Whereas the previous studies utilized the bulk sequencing providing only a fraction of specific marker genes^27^, our approach enables to obtain the complete picture of transcriptional states across the entire differentiation timeline of odontoblasts.

The comparison between mouse growing and nongrowing tooth showed high homogeneity of mouse molar pulp populations. Therefore, the diversity of mesenchymal populations in the self-renewing incisor can be largely explained by the necessity to maintain growth and self-renewal. Despite mouse molar pulp homogeneous appearance, human nongrowing molar pulp showed a clear presence of several distinct subpopulations, which differed by the specialized matrix production and some other parameters. For instance, the apical papilla part, being the growing region of an unerupted human tooth, demonstrated corresponding growth-related cell-type heterogeneity. At the same time, the fully grown and erupted human molar teeth also preserved apical pulp-like transcriptional aspects in some pulp subpopulations, which, for instance, might be taken advantage of during reparative response. Thus, the preservation of some residual apical-distal or growth-related heterogeneity aspects in growing and fully grown human molar teeth highlights the key aspects of heterogeneity of human dental pulp transcriptional states. The functional meaning of these growth-related aspects will require further analysis in experimental ex vivo and in vivo settings. At this point, our data of such heterogeneity markers (also including signatures for proliferative populations) can serve as a guide for the isolation and culture of mesenchymal stem cells for tissue engineering and fundamental understanding of different pulp cell subtypes.

Finally, we addressed the heterogeneity of immune cells in mouse and human teeth including macrophages. The analysis revealed the human-specific aspects of macrophage localization, which suggest better protection of mouse teeth versus human. The predominant concentration of human macrophages in odontoblastic and periodontoblastic space suggests the existence of unknown cell–cell interactions and heterogeneously distributed homing factors that can be potentially tackled for increasing the protection of our teeth against infections.

Overall, we hope that the presented detailed and validated map of dental cell types, supplemented by human comparison, will serve as a key resource stimulating further studies of cell dynamics in tooth morphogenesis, also including reparative and regenerative therapies.

## Methods

### Animals and human tissue

All animal experiments were approved by the Ethik-Kommission der MedUni Wien zur Beratung und Begutachtung von Forschungsprojekten am Tier in Austria as well as Ethical Committee on Animal Experiments (Stockholm North Committee) in Sweden and performed according to the Austrian, UK, Swedish and international regulations. All mice were kept under SPF conditions in 12/12 light/dark cycle, 18–23 °C and 40–60% humidity. Experiments with human samples were performed with the approval of the Committees for Ethics of the Medical Faculty, Masaryk University Brno & St. Anne´s Faculty Hospital (No. 13/2013) and Ethik-Kommission der Medizinischen Universität Wien (No. 018/03/2018, 631/2007). Written informed consent was obtained from all participants, in-line with the Declaration of Helsinki. *Fos*^*CreERT2*^*/R26*^*ZsGreen1*^ strain was used for genetic tracing of outer enamel epithelium progenitor cells. *DMP1-Cherry/DSPP-cerulean* mice were used for visualization of alveolar bone^38,49^. *Acta2*^*CreERT2*^*/R26*^*tdTomato*^ mice were used for lineage tracing of *Acta2*^+^ dental epithelial stem cells in cervical loop^31^. *Sox2-GFP* animals were used to enrich epithelial stem cells population for single-cell sequencing^25^. *Foxd1*^*CreERT2*^*/Ai9* mice were used for lineage tracing of dental mesenchymal stem cells^50^. Mice used for all experiments were sacrificed by an isoflurane (Baxter KDG9623) overdose. Human teeth were extracted for clinically relevant reasons at Clinic of Stomatology, St. Anne’s Faculty Hospital, Brno, Czech Republic or Department of Oral Surgery, Medical University of Vienna, Austria.

### Tissue handling and staining

Mice used for all experiments were sacrificed by an isoflurane (Baxter KDG9623) overdose, mandibles were carefully dissected out, fixed in 4% paraformaldehyde pH 7.4 for 5–15 h, decalcified in 10% EDTA pH 7.4 for 7 days at +4 °C, cryopreserved in 30% sucrose overnight at +4 °C and embedded in OCT medium (Tissue-Tek, 4583) on dry ice. Samples were cut on cryostat (Leica CM1850UV) in sagittal orientation as 14-μm thick sections. Human teeth extracted for clinically relevant reasons were fixed in 4% paraformaldehyde pH 7.4 for overnight, decalcified in 10% EDTA pH 7.4 for 7 days at +4 °C and paraffin embedded. Samples were cut on microtome (Leica SM2000R) as 2-μm thick sections. Before antibody staining antigen retrieval was performed (Dako S1699). Staining with primary antibodies was performed overnight at room temperature followed by Alexa-conjugated secondary antibodies staining at room temperature for 1 h (Invitrogen, 1:1000) or HRP-conjugated streptavidin-biotin antibody and immunoreactivity was visualized with ImmPACT DAB Peroxidase (Vecor Laboratories, SK4105).Used antibodies: ACTA2 (Protein Tech, 23081-1-AP; 1:500), AIF1 (Novus, NB100-1028; 1:500), CALB1 (Swant; CB-38a; 1:500); COL4 (AbD Serotec, 2150-1470; 1:500), CDH1 (Novus, Af748; 1:500), CSF1 (NSJ, R31901; 1:200), CLDN10 (Sigma–Aldrich, HPA042348; 1:200), DLX5 (LSbio, LS-C352119; 1:200), DPP4 (Novus, AF954; 1:200), EGR1 (Cell Signalling, 4154; 1:200), F4/80 (Abcam, ab6640; 1:200), LYVE1 (Novus, AF2125; 1:200), MKI67 (Zytomed, RBK027-05, 1:200), NOTUM (Sigma–Aldrich, HPA023041; 1:200), PIEZO2 (Sigma–Aldrich, HPA040616; 1:200), POSTN (Novus, NBP1-30042; 1:200), RYR2 (ThermoFisher, PA5-36121; 1:200), S100A13 (DAKO; IS504; 1:500), SALL1 (Abcam, ab31526; 1:200), SMOC2 (MyBioSource, MBS2527784; 1:200), SOX9 (Sigma–Aldrich, HPA001758; 1:200), SOX10 (Santa cruz, sc-365692; 1:200), THBD (RnD systems, MAB3894; 1:200). Cell nuclei counterstaining was performed with DAPI (Sigma–Aldrich, D9542) diluted 1:1000 in PBS + 0.1% Tween 20 (Sigma–Aldrich, P9416) and slides were mounted with 87% glycerol (Merck, 104094) or Fluoromount Aqueous Mounting Medium (Sigma–Aldrich, F4680). Imaging was performed using Zeiss LSM880 laser scanning confocal microscope and Lightsheet Z.1 microscope. ZEN2.1 (ZEISS) and Imaris (Bitplane) software was used for image processing. Conventional histological staining after Clodrosome or Encapsome treatments was performed after 4 weeks decalcification of dissected mandibles in 19% EDTA. Mandibles were embedded in wax blocks and sectioned using 8μm thickness. Sections were stained using Masson’s Trichrome.

### RNAscope

*C56Bl6/J* mice (7 days to 4 month old) were used to verify scRNA-seq candidate gene expression. Dissected mouse mandibles were fixed in 4% paraformaldehyde pH 7.4 overnight, decalcified in 10% EDTA for 7 days at +4 °C for 7 days (*Foxd1, Sfrp2*) or in 0.5 M EDTA at +4 °C for 20 days (*Gjb3, Krt15,* and *Igfbp5*). All samples were embedded in paraffin and sectioned at 7 μm. Tissue were subsequently processed using the RNAscope multiplex fluorescent detection reagents v2 (ACD, 323110) (*Foxd1, Sfrp2*) or RNAscope 2.5 HD Assay-RED detection kit (ACD, 322350, 322360) (*Gjb3* (508841), *Igfbp5* (425731), *Smpd3* (815591), *Dkk1* (402521), *Wnt6* (401111), *Wisp1* (501921), *Nupr1* (434811), *Syt6* (449641), *Tac1* (410351)) according to the manufacturer’s instructions. Notably, slides were boiled in the target retrieval buffer and incubated in Protease Plus solution at 40 °C for 15 min before probes were incubated at 40 °C for 2 h. The following probes were used: *Foxd1* (495501), *Gjb3* (508841), *Igfbp5* (425731), *Sfrp2* (400381). Samples were counterstained either with DAPI for 30 s (*Sfrp2* and *Foxd1*) or with Hematoxylin Gills #2 (20% dilution) for 15 s, followed by 10 s in ammonium hydroxide. Imaging was performed using a Leica DM5000 B (*Gjb3, Igfbp5*) or Zeiss LSM880 laser scanning confocal microscope (*Foxd1, Sfrp2*).

### Statistics and reproducibility

Images: 1e; 2b–d, f; 3a–k; 5d, e, f, i; 6d, f; 7g; 8e–j; 10c, d and Supplementary Fig. 5c–f were selected as a representative pictures. The same or similar results were obtained in >3 independent experiments.

### Single-cell preparation

*Mouse*: Wild-type *C56Bl6*, and *Sox2-GFP* mice were used for cell isolation from mandibular incisors for single-cell transcriptomics experiments. Age of all mice used for single-cell experiments was between 2 and 4 months. Mice were sacrificed by isoflurane overdose. Mandibles were carefully dissected and under stereomicroscope and surrounding soft tissue was removed. Using scalpel and scissors mandibular bone was gradually removed to obtain separated incisor. Particularly careful handling was performed in the soft area around the most proximal part of incisor where cervical loops. Both epithelial and mesenchymal parts from the whole of incisor were together dissected and processed as described further.

*Human*: Tissue from adult human healthy molar pulp, adult human molar pulp with caries and apical papilla from growing human molar was harvested for isolation of cells for RNA-seq analysis. Adult human healthy molar and adult human molar with caries were sectioned carefully to avoid damaging pulp using dental drill through enamel and part of dentin in mesial-distal direction. Dental pulps from adult molar and dental papilla were harvested, sectioned on petri dish in droplet of HBSS (Sigma–Aldrich, H6648) on ice into small pieces and further processed in the same way as mouse tissue.

Human tissue or mouse dental pulps with dental epithelium and surrounding dental follicle were isolated, cut into small pieces, transferred to 15 mL falcon tube with 2,5 mL Collagenase P (3 U/mL; Sigma–Aldrich, COLLA-RO ROCHE) dissolved in HBSS and incubated for 20–30 min at 37 °C shaking (120 rpm). During enzymatic digestion, tissue pieces were homogenized three times using 1 ml pipet. After incubation, the suspension was finally homogenized using pipet and 10 mL of 2% FBS (ThermoFisher Scientific, 10500064) in HBSS were slowly added. The suspension was centrifuged in 4 °C precooled centrifuge for 10 min at 300 × *g*. After centrifugation, supernatant was removed, the pellet was resuspended in 1 mL 2% FBS in HBSS, suspension was filtered using Tubes with Cell Strainer Snap Cap (Corning, 352235) and FACS (BD FACSAria III; software used: BD FACSDiva 8.0.1) was performed. All the work (except of enzymatic digestions at 37 °C) was performed on ice.

### Numbers of used mice for single-cell RNA-seq analyses

For analysis of adult healthy mouse-incisor 78 mandibular incisors were used out of 39 animals in total. For analysis of mouse molar pulps 48 first molars out of 12 adult animals were used in total. For adult human tooth analyses 7 wisdom molars out of 7 healthy randomly selected males and females of age 18–31 were used and 6 apical papillae out of 3 patients/teeth were used.

### Single-cell sorting and single-cell transcriptomics

All the sortings were performed on BD FACSAria III Cell Sorter into pre-prepared 384-well plates with lysis buffer. To minimize time of the cells outside the body no viability staining was performed. Three gating aspects were selected for isolation of non-traced cells: (a) SSC-A/FSC-A, (b) FSC-A/FSC/W, (c) SSC-A/SSC/W and strict gates were applied to remove debris, dead cells, and doublets. When genetically traced organisms were used the fourth gate (d) was applied during FACS sorting. Negative control using wild-type organism was applied to make a correct gating (e). After sorting, plates were frozen on dry ice and until being processed kept at −80 °C. Single-cell sequencing was performed according to smart-seq2 protocol following published guidelines^12^.

### Flow cytometry

P7 incisor pulp was extracted in ice cold PBS and cut into small pieces using a fine scissor. The pulp was then resuspended in 5 ml of Collagenase D (0.5U/ml, Roche, 11088866001) and Dispase II (1.5U/ml, Roche, 4942078001). The tissue was allowed to dissociate by incubating the suspension in a cell culture incubator at 37 °C in 5% CO_2_ for 30 minutes. Following enzymatic digestion the cell suspension was filtered through a 70-um Falcon Cell Strainer (Falcon, 352350) and the enzyme reaction quenched using 10 ml of ice cold PBS. Cells were centrifuged at 300 × *g* for 10 minutes, and resuspended in 200 ul of FACS staining buffer (BioLegend, 420201). 0.10 ug of rat anti mouse Gr-1—Alexa Fluor 488 conjugated (108417, BioLegend), and rat anti mouse F4/80—APC conjugated (123116/BioLegend) were added to the cell suspension. Cells were incubated with the antibodies on ice for 30 min. Excess staining buffer was added to quench the reaction and cells were centrifuged twice as before to remove excess antibody. Following the final centrifugation, cells were resuspended in 500 ul of staining buffer and 1.5 ug of DAPI (D1306, Invitrogen) added to be used as a dead cell exclusion marker. Samples were then analyzed on BD FACSAria III fusion machine. Data analysis was performed on FlowJo v10 software. Cells were gated based on size using standard SSC-A and FSC-A parameters so that debris is excluded. Following gating of cells, we focused on single cells and excluded doublets using SSC-A and SSC-W parameters. Live cells were then selected as cells identified to be dimly fluorescing in DAPI. Appropriate gating strategies were then used to select cells positive for the antibodies being used as deducted from the use of unstained controls.

### Data processing of mouse incisor

The reads were aligned to the UCSC mm10 genome assembly, and per-gene read counts in each cell were determined using feature Counts software. STAR aligner was used to align scRNA-seq reads. The cells were filtered to exclude those with fewer than 800 detected genes resulting in 2889 out of 3312 cells for further analysis. Only genes that had at least 60 reads in at least 30 cells were considered for downstream processing. The data were analysed using PAGODA using k-nearest neighbour error models (k = 20 and plain batch correction across samples). Gene expression levels were normalized per mean expression level in every cell and log10 transformed (abbreviated below as fpm). Annotated Gene Ontology (GO) categories and de-novo gene clusters showing statistically significant overdispersion (z-score > 2.3) were clustered to determine the top aspects of transcriptional heterogeneity. Mitotic signature was removed from gene expression values by regressing out the mitotic expression signature, as previously described using a set of cell-cycle-related genes from^6,31^. The cells were grouped in 17 clusters using unbiased clustering as determined by PAGODA. t-SNE embedding was generated using *Rtsne* package and PAGODA-based cell–cell distance with perplexity = 25. Expression of a set of genes, where it is shown, was defined as their average expression for each cell.

To characterize gene modules controlling cell-type identities, we selected genes that have at least 2 fpm difference between maximum and mean average expression among clusters. For supplementary heatmap of general dataset genes were clustered using hclust() and cutree() R functions in 20 clusters using hierarchical clustering with Euclidean distance between gene expression profiles using Ward’s linkage and cells were arranged using PAGODA clustering described above. Similar procedure was used to characterize gene modules separately in epithelial (552 genes) and other compartments using different cutoffs of 1.5 and 0.5 fpm difference on expression between maximum and mean average expression among clusters.

### Epithelial compartment

Three epithelial clusters comprising 268 cells altogether were identified based on high expression of Krt14 and reanalyzed separately. Gene expression levels in epithelial cells were adjusted to account for variance-mean trend in cell–cell expression variability as defined by PAGODA (knn = 40). Epithelial cells were grouped in 13 clusters by hierarchical clustering with Ward linkage using correlation-based cell–cell distance of expression levels of 10410 the most variable genes (standard deviation variance-adjusted expression levels >0.8) in epithelial compartment. t-SNE embedding was generated using the same cell–cell distance and perplexity = 20. Mitotic signature was calculated as average expression of mitotic genes.

Five clusters representing progressive ameloblast differentiation were identified based on known markers of respective stages. Heterogeneity of ameloblasts cells was modelled as a principal trajectory using crestree R package approach^51^, with parameters (lambda = 100, sigma = 0.03, M = 100) and cosine-based cell–cell distance. Root of the reconstructed trajectory was selected to biologically correspond to progenitor population of ameloblasts and each cell was assigned pseudotime as a distance from the root along the trajectory. Gene expression levels were modeled as a function of pseudotime using splines of the fifth degree with *gam* function from *mgcv* R package. Significance of association was calculated as Benjamini–Hochberg adjusted *gam* estimates of spline *p* value. Fitted gene expression levels were used to estimate magnitude of expression levels variation along the trajectory and downstream clustering. Five hundred and fifty-six genes that had more than 100-fold magnitude differences along the trajectory and adjusted *p* value < 10^−5^ were clustered in nine clusters using hierarchical clustering with Ward linkage based on Euclidean distance.

### Mesenchymal compartment

Four mesenchymal clusters of the general dataset comprising 1111 cells were reanalyzed separately. Only genes that had at least 20 reads in at least 10 cells were considered for downstream processing using PAGODA (k = 20 and plain batch correction across wells). Mitotic signature was regressed out and top aspects of transcriptional heterogeneity were determined as for general dataset. The cells were grouped in five clusters using unbiased clustering as determined by PAGODA and t-SNE embedding was generated using PAGODA-based cell–cell distance with perplexity = 20. To clean up non-mesenchymal admixture of cells from other populations, only 1042 cells that had mean correlation of more than 0,2 to 200 the most correlated cells were retained for further analysis.

Transcriptional states of mesenchymal cells were modelled as a principal tree using our *crestree* R package based on the SimplePPT approach^51,52^. Briefly, given a set of data points x_1,..,x_N in M-dimensional space, a set of principal points z_1,..,z_K are arranged and are connected as a tree in the same space. Positions of principal points and tree structure are learned as alternate convex optimization problem that balances overall proximity of prinicipal tree to data points and stringency of the tree. Tree was learned with parameters (lambda = 2000, sig = 0.03, M = 200) and cosine-based cell–cell distance. Principal tree contained three major branches and a few small sporadic branches that were removed.

For analysis of odontoblasts differentiation, cells assigned to a branch leading to mature odontoblasts were isolated and projected to the first two principal component using *pcaMethods* R package. PCA was performed using 259 the most overdispersed genes (standard deviation variance-adjusted expression levels >1.3) whose expression was adjusted to account for variance-mean trend as described in PAGODA. The first principal component (PC1) corresponded to transition of progenitor population to odontoblasts and was used as cell pseudotime. To identify differentiation-associated genes, expression levels were modeled as described for epithelial trajectory modeling. For heatmap visualization 252 genes that had magnitude of fitted expression levels of more than 1 fpm difference along PC1 and adjusted *p* value < 10^−5^ were arranged by pseudotime of maximum expression and pseudotime of the first derivative pass of ratio of expression magnitude to pseudotime magnitude. Sharp transition in expression pattern along pseudotime marked transition point from progenitor population to preodontoblasts and was used to separate progenitor population. To identify genes associated with distant and periodontoblastic pulp trajectories, cells assigned to one of pulp branches and cells of progenitor population from odontoblastic branch were arranged by pseudotime defined for the whole tree. Expression levels were modeled by a function of pseudotime as described above. For each of two pulp trajectories 100 genes with the highest magnitude along pseudotime and adjusted *p* value < 10^−4^ are shown.

To identify sources of heterogeneity in progenitor mesenchymal cells, all mature populations were removed using the following procedure: first, we selected the only one of five unbiased clusters does not represent mature pulp populations; second, we removed preodontoblasts from the cluster (Suppl. Fig. 4B) thus retaining only progenitor or immature committed cells (immature subpopulation below). Only 5343 the most overdispersed genes (standard deviation variance-adjusted expression levels across progenitor >0.9) in immature subpopulation were considered and their expression levels were normalized to zero mean and unit dispersion among immature cells. Independent components (ICs) of transcriptional variability were identified by independent component analysis (ICA) using *icafast()* function from *ica* R package. The number of statistically meaningful components was identified by comparison of components stability with that of control expression matrix, the latter obtained by shuffling of expression levels among immature cells independently for each gene. Twenty components of ICA were calculated for full original and control matrices and for their 100 subsamplings of 70% of cells. Stability of an IC of full matrix is estimated as the average correlation with the most similar components among 100 subsamplings. It reveals that all ICs of control matrix and 15 ICs of original matrix have stability of about 0.4, while 5 ICs of original matrix have substantially higher stability indicating confident statistical signal behind them. Final five ICs were predicted by running ICA with nc = 5.

We next analysed larger sample of 2552 mesenchymal cells profiled with 10x Chromium. Mesenchymal cells were isolated as clusters among 10× Chromium mouse-incisor populations expressing known mesenchymal marker, excluding minor admixture of epithelial cells in clusters based on expression of *Epcam, Krt14*, or *Cdh1*. The cells were reanalysed using standard PAGODA2 processing, including normalization of expression levels per mean in every cell, log10 transform and dimensionality reduction to 20 principal components (conducted with correction of expression levels for mean-variance trend). Mesenchymal cells were grouped in 12 clusters using default PAGODA2 multilevel community detection method. Three follicle clusters were merged, while nine dental pulp clusters were coloured to reflect the most similar cluster colour of Smart-seq2 annotation (see Fig. 4a). For analysis of fate-specific expression programs, 20 genes of each fate that have the largest mean expression difference between a fate cluster (e.g. apical, distal and pre-odontoblastic clusters) and progenitor clusters were considered as fate-specific markers. Intensity of fate-specific expression program in each cell was estimated as mean expression among 20 fate-specific genes. Cell-cycle score was defined as first principal component of cells transcriptional variability based on cell-cycle-annotated genes from. Cells from a cluster of mitotic cells were projected onto t-SNE embedding of non-mitotic mesenchymal landscape as a mean position of 10-nearest neighbours non-mitotic cells, where neighbours were defined using cosine-based distance in dimensionally reduced space of 20 PCs.

### Immune cluster analysis

We isolated four clusters of cells representing immune subpopulations, partitioned them in eight clusters using hierarchical clustering with Ward linkage and visualized using t-SNE with perplexity = 20. For clustering and visualization 1-cor(.) cell–cell distance was used restricted to 1739 the most overdispersed genes (standard deviation variance-adjusted expression levels across progenitor >1.1) in immune compartment as estimated by standard deviation of mean-variance trend adjusted expression levels.

### Pericytes, glia, and endothelium analysis

Subpopulations of pericytes and endothelial cells were partitioned in three groups each, while glial cells were partitioned in two groups using hierarchical clustering with Ward linkage. Clustering, t-SNE visualization and PCA were based on mean-variance-adjusted expression levels restricted to the most overdispersed genes in each compartment (glia: 873 genes, endothelium: 1878 genes, pericytes: 2110 genes; standard deviation variance-adjusted expression levels across progenitor >1.5 for glia, 1 for endothelium, 1 for pericytes)). 1-cor(.) cell–cell distance was used for clustering and t-SNE (perplexity = 20).

### Assessment of cell quality

Cell quality was additionally probed using metrics reflecting expression complexity, mitochondrial content and doublet probabilities. Toward that goal, we assessed tradeoffs between number of expressed genes and UMIs (or reads for Smart-seq2) per cells, fraction of total reads from mitochondrial genes and cell doublet probabilities estimated using Scrublet with default parameters^53^. We estimated and explored these metrics for four datasets and manually excluded one cluster that had low number of genes compared to UMIs (it was a cluster of spike-in cells, see below) and a number of clusters of joint human analysis that were likely doublets (see Extended Data Fig. 2e–h).

### Data preprocessing of 10x Chromium samples

CellRanger- 10× Chromium software was used to perform alignment to GRCh38 human genome or mm10 mouse genome assemblies, filtering, barcode counting and UMI counting. For Apical papilla 1, Adult molar 3, Adult molar 4, incisor (10×), mouse molar 1 (10×) datasets preprocessing was performed using CellRanger-2.2.0 following by filtering of cells having less than 500 UMIs. For other datasets datasets preprocessing was performed using CellRanger 3.0.2 following by default CellRanger 3.0.2 filtering of cells. Additionally, a protocol of library preparation used by the facility included spike-in of Jurkat and 32D cells of human and mouse species. Spike-in cells were not used for data processing or analysis and were excluded as *Hbb*^+^ clusters; they are also easily detectable as having low complexity and forming a separate outlier transcriptional cluster.

### Joint analysis of mouse datasets

Two mouse-incisor datasets (10x Chromium, 4236 cells, and Smart-seq2,2889 cells) and three mouse molar datasets (two 10× Chromium, 1460 and 384 cells, and Smart-seq2, 195 cells), each composed of multiple teeth (see chapter “Numbers of used mice for single-cell RNA-seq analyses” in materials and methods and Supplementary Table 2), were processed independently using PAGODA2 R package^13^ routine basicP2proc(), which performs normalization, log transformation, correction for mean-variance trend of expression levels, dimensionality reduction via PCA and clustering. After filtration of spike-in clusters identified through expression of Hbb, processed datasets were then jointly analysed using CONOS R package, which enables integrative analysis of single-cell datasets across samples and conditions^40^. Joint graph of 9164 cells from all datasets was constructed using CONOS routine buildGraph() with nearest neighbour parameters k = 15, k.self = 15, k.self.weight = 0.1 in space of 10 common principal components (CPCA) estimated using 1000 overdispersed genes for each pair of samples. Joint graph was layout in 2D using UMAP method through CONOS routine embedGraph() with parameters spread = 1 and min.dist = 0.05. Graph-based leiden community method with resolution = 1.0 was used to partition cells in 22 clusters.

To provide additional statistical support for reproducible structure of mesenchymal incisor populations and homogeneous distal-like molar state, we computationally isolated clusters of mesenchymal cells and separately explored cells of Smart-seq2 and 10x Chromium platforms. Platform-specific mesenchymal cells were processed using routine basicP2proc() with default PAGODA2 batch correction across samples and dimensionality reduction to 10 principal components based on top 1000 overdispersed genes. t-SNE method with perplexity = 50 was used to make 2d embedding.

Differential gene expression between molar and distal incisor mesenchymal cells was estimated as fold change between cluster-specific expression levels, estimated as sum of gene reads to total reads in a cluster. Significance of expression changes was estimated as *p* value of *t*-test comparing group means between normalized expression levels of molar and distal incisor clusters.

### Joint analysis of human datasets

Two growing apical papilla and five adult molar 10× Chromium datasets of human teeth, comprising totally 41673 cells, were analysed using single-cell variational inference (scVI) deep learning framework^54^. Gene space was subsampled to 3000 genes using scVI subsample_genes routine following by setting up parameters of variational autoencoder using VAE() routine with parameters (n_hidden = 128, n_latent = 30, n_layers = 2 and dispersion = ’gene’) and training it using UnsupervisedTrainer() routine with n_epochs = 150. Batch effect correction was by default performed using harmonization approach^55^. The resulting 30-dimensional reduced scVI space was used to make 2d embedding of the datasets using UMAP() routine with parameter spread = 1. K-nearest neighbour cell graph (k = 30) was constructed using cosine-based cell–cell distances estimated in scVI space and then used to make leiden clustering (resolution = 1).

To explore behaviour of apical and distal mouse-incisor gene modules across human mesenchyme populations, we chose apical-specific and distal-specific sets of genes and assessed their averaged expression in human mesenchyme clusters. In details, apical and distal-specific genes were defined as those having at least three-fold change between apical and distal incisor states and *p* value < 10^−10^ in both 10 Chromium and Smart-seq2 mouse-incisor datasets. We then calculated (1) average expression levels of each apical and distal-specific gene in human mesenchyme clusters and (2) cell-specific sum of expression levels of apical genes or distal genes.

Similarity of nondividing cells to a group of dividing mesenchymal cells was estimated as an average Pearson correlation with dividing cells in latent scVI space.

### Assignment of sub/clusters identities

Cell clusters and subclusters were characterized on several levels. After the unbiased cell clustering based on the expression similarities we searched for the most specific and highly expressed genes for every main sub/cluster and performed manual literature search to define their identity. Every sub/cluster was characterized based on co-expression of several (5–10) genes known to be expressed in particular cell sub/type. For the small subclusters where the identity wasn’t possible to determine by literature search because their identity was unknown we performed either immunostainings or in situ hybridizations to determine their histological location. Based on the position of these cells in the tissue and a specific expression patterns of the selected genes we could then assess their role in the tissue. No functional experiments to prove such a role was not performed.

### Reporting summary

Further information on research design is available in the Nature Research Reporting Summary linked to this article.

## Supplementary information

Supplementary Information

Reporting Summary

Description of Additional Supplementary Files

Supplementary Data 1

## Data Availability

All single-cell RNA-seq datasets have been deposited in the GEO under accession code GSE146123. Processed data and interactive views of datasets can be accessed on the authors’ website: [http://pklab.med.harvard.edu/ruslan/dental.atlas.html].

## Code availability

Code is freely available on the authors’ website: [http://pklab.med.harvard.edu/ruslan/dental.atlas.html].
