# Supplementary material for: Dental cell type atlas reveals stem and differentiated cell types in mouse and human teeth
Source: Nat Commun. 2020 Sep 23;11:4816. doi: 10.1038/s41467-020-18512-7 (PMC7511944; doi:10.1038/s41467-020-18512-7)
Supplement: Supplementary file 1 — Supplementary Information [file 41467_2020_18512_MOESM1_ESM.pdf]

Dental cell type atlas reveals stem and differentiated cell types in mouse and human teeth

Krivanek et al.

SUPPLEMENTARY FIGURES

Supplementary Figure 1

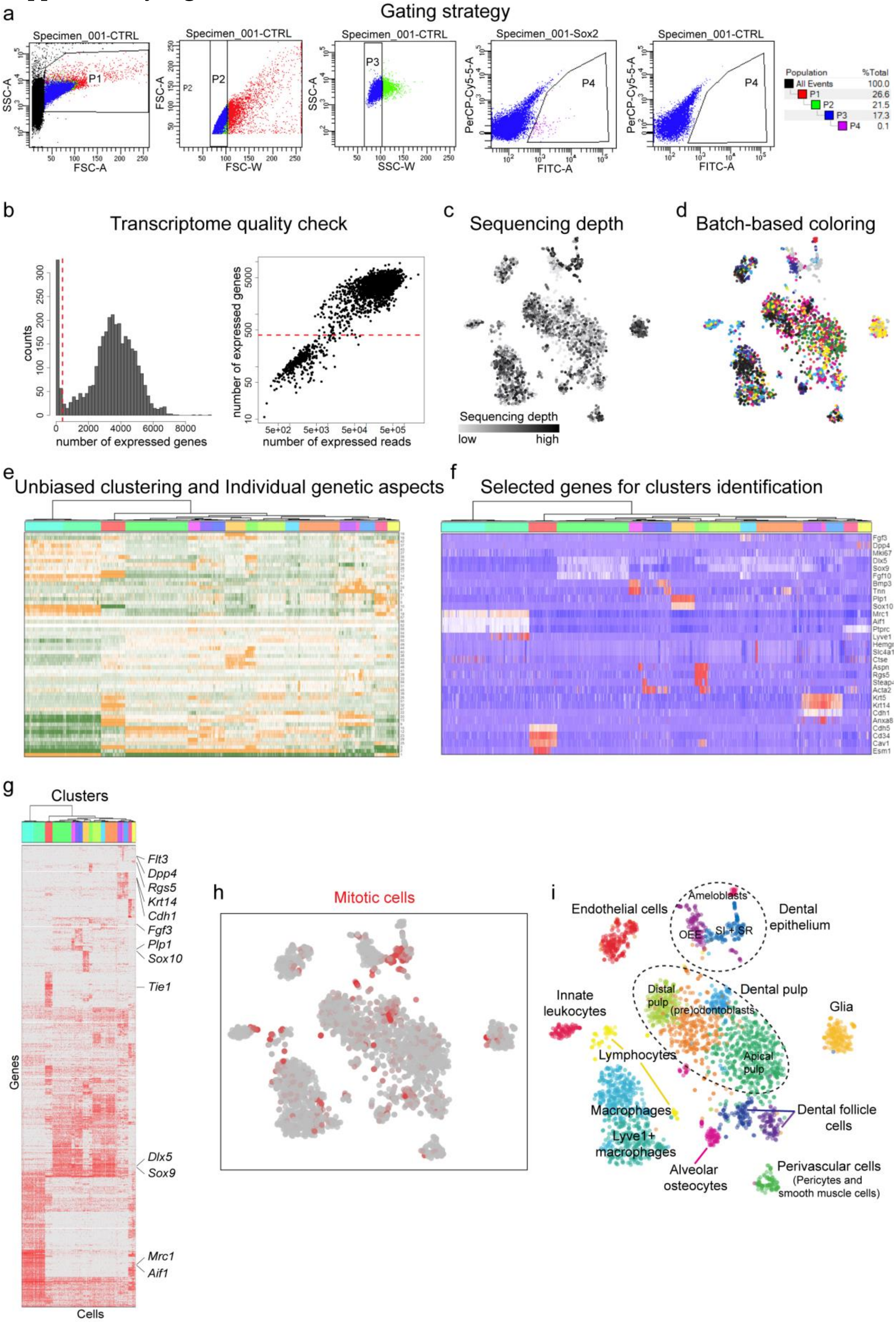

Supplementary Figure 1. Transcriptional patterns of major cell populations in the mouse incisor.

a) Plots showing an example of gating strategy used for the single-cell sorting into 384-well plates for smart-seq2 protocol.

b) Number of reads and expressed genes per cell. Bimodal distribution of the number of expressed genes per cell (left) and high correspondence between number of reads and expressed genes per cell (right) allows to unambiguously identify and to exclude low quality cells.

c) Depth (number of reads) per cell and batches are only subtly associated with structure of clusters.

d) Batch effect. Plot shows the cell clustering based on different isolations (each color shows separate single-cell RNA-seq analysis).

e) Individual multigenic aspects of heterogeneity identified for the full dataset.

f) The selection of comprehensive and known markers outlining the clusters.

g) Heatmap showing combinatorial expression patterns of genes strongly associated with subpopulations used for cluster identification.

h,i) t-SNE dimensional reduction visualizes mitotic cells **(f)** among all major populations **(g)** identified in the whole dental cell type dataset.

(SI – stratum intermedium, SR – Stellate Reticulum, OEE – Outer Enamel Epithelium)

Supplementary Figure 2

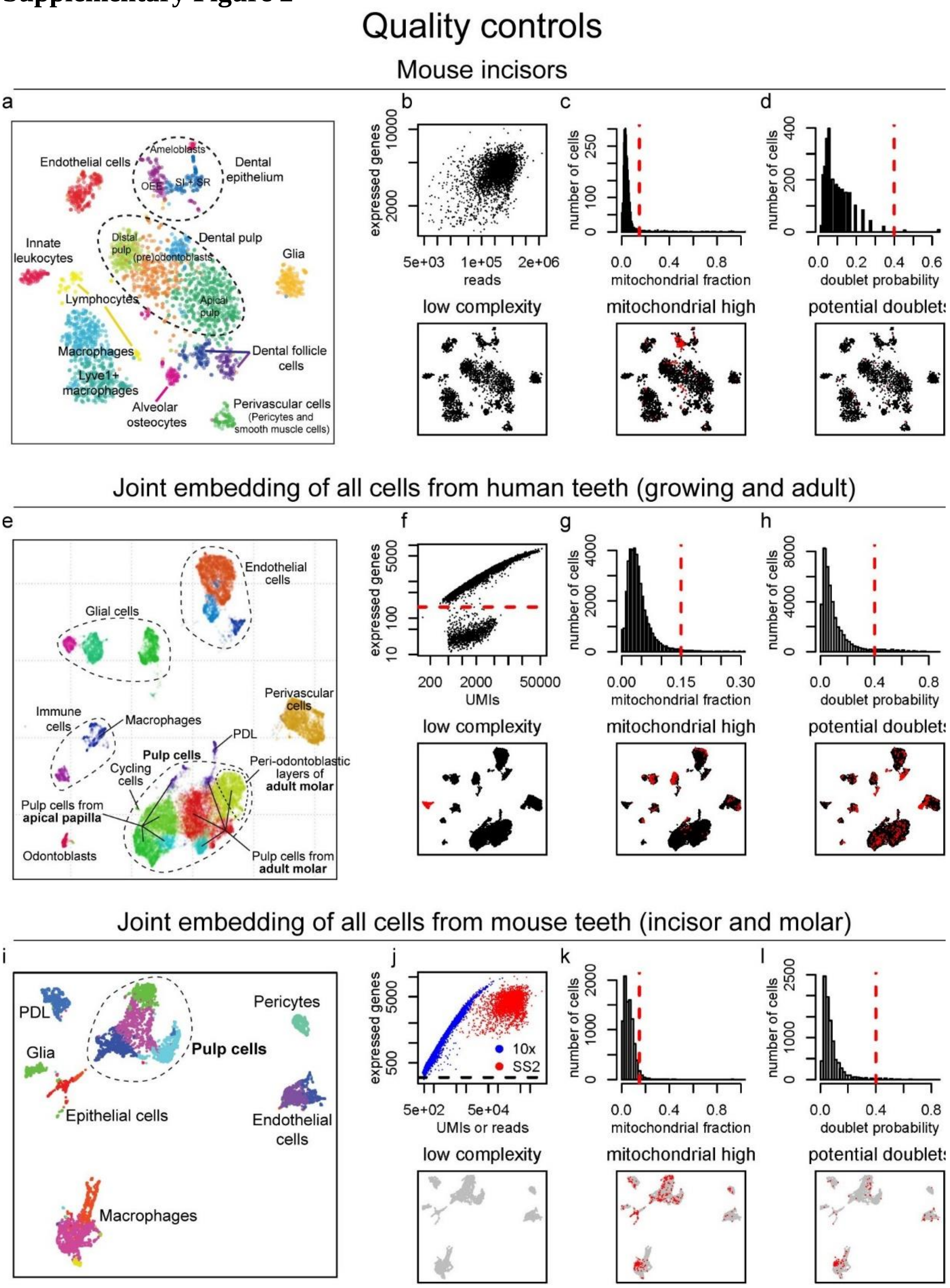

Supplementary Figure 2. Assessment of cell quality using three diagnostic metrics across four meta-datasets.

a,e,i) Embeddings of four meta-datasets corresponding to Extended Data Figure 9, Figure 4, Figure 1, Extended Data Figure 13.

b,f,j) Relationship between number of UMIs (or reads for Smart-seq2) and number of expressed genes per cell (upper panel) reveals an outlier population with low complexity in

the human meta-dataset, panel f). Red cells in embeddings reflect cells with lower than 300 expressed genes (horizontal dotted line).

c,g,k) Distribution of fraction of mitochondrial reads per cells (upper panel) shows preferentially high-quality cells. Bottom plots show low-quality/suspicious cells onto embeddings (0.15 cutoff, see vertical red dotted line on the top plots).

d,h,l) Assessment of doublets in meta-datasets using Scrublet inferred doublet probabilities. Distribution of doublet probabilities (upper plots) reveals a number of cell groups in human meta-dataset likely composed of doublets, panel h). Cells with doublet probability of more than 0.4 are shown onto embeddings (bottom plots).

Supplementary Figure 3

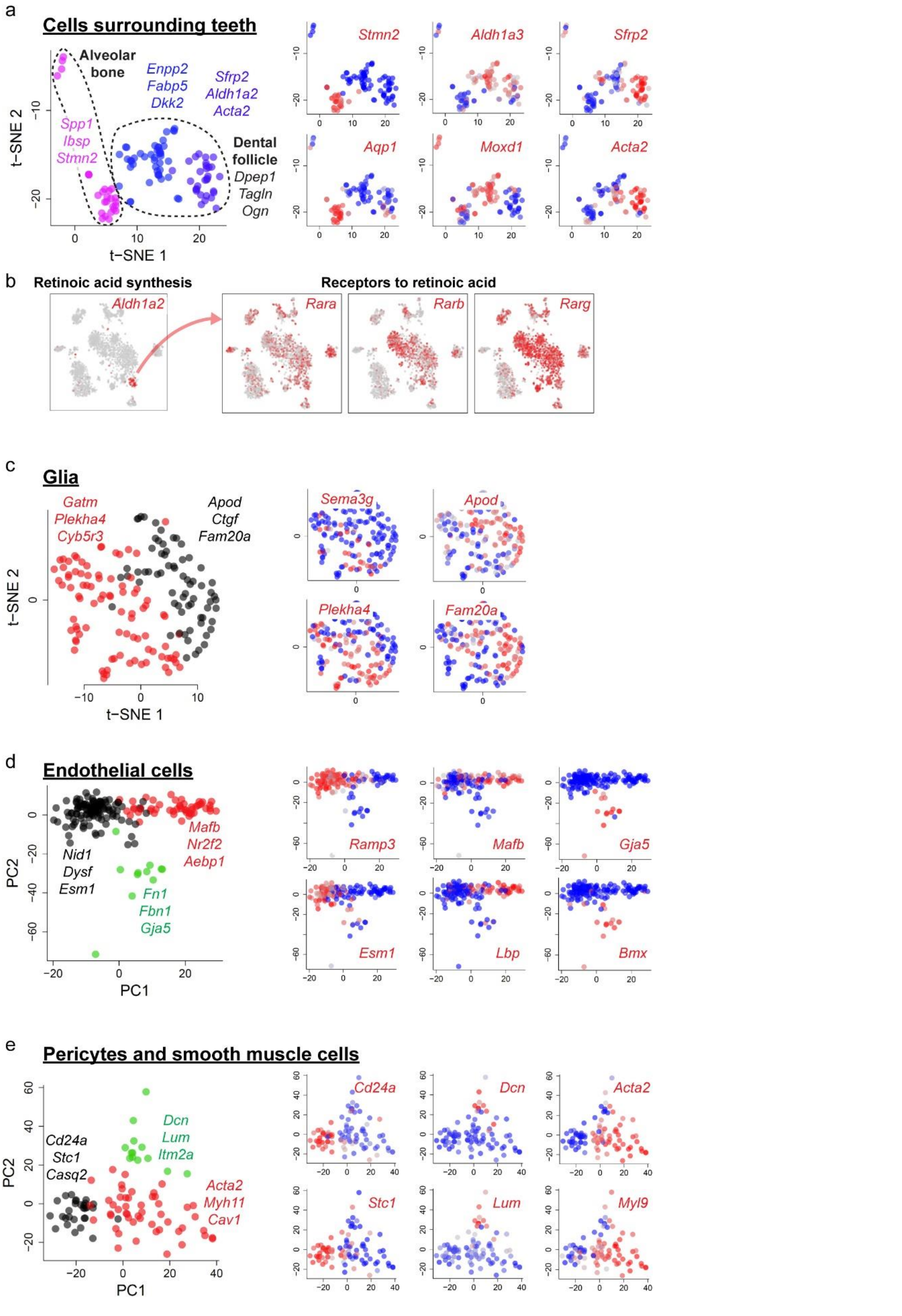

Supplementary Figure 3. Heterogeneity of Cells surrounding teeth, Glial, Endothelial and Perivascular cells from the incisor tooth.

a) Heterogeneity of cells surrounding the incisor. Two major cell populations can be distinguished: alveolar bone marked by *Ibsp, Bmp3, Dmp1, Spp1, Stmn2, Tnn* and *Ltbp2*, and the dental follicle marked by *Aldh1a2, Acta2, Tagln, Gdf10, Cldn2,* and *Igfbp5*.

b) The expression of *Aldh1a2* in the dental follicle suggests previously unanticipated roles of retinoic acid signalling in incisor maintenance. Correspondingly, complementary receptor genes *Rara, Rarb* and *Rarg* are expressed in some of the major populations of the tooth.

c) Internal heterogeneity of glial cluster.

d) Internal heterogeneity of endothelial cluster.

e) Internal heterogeneity of perivascular cells. Note: the red cluster outlined by expression of *Acta2, Myh11* and *Cav1* represents perivascular smooth muscle cells.

Supplementary Figure 4

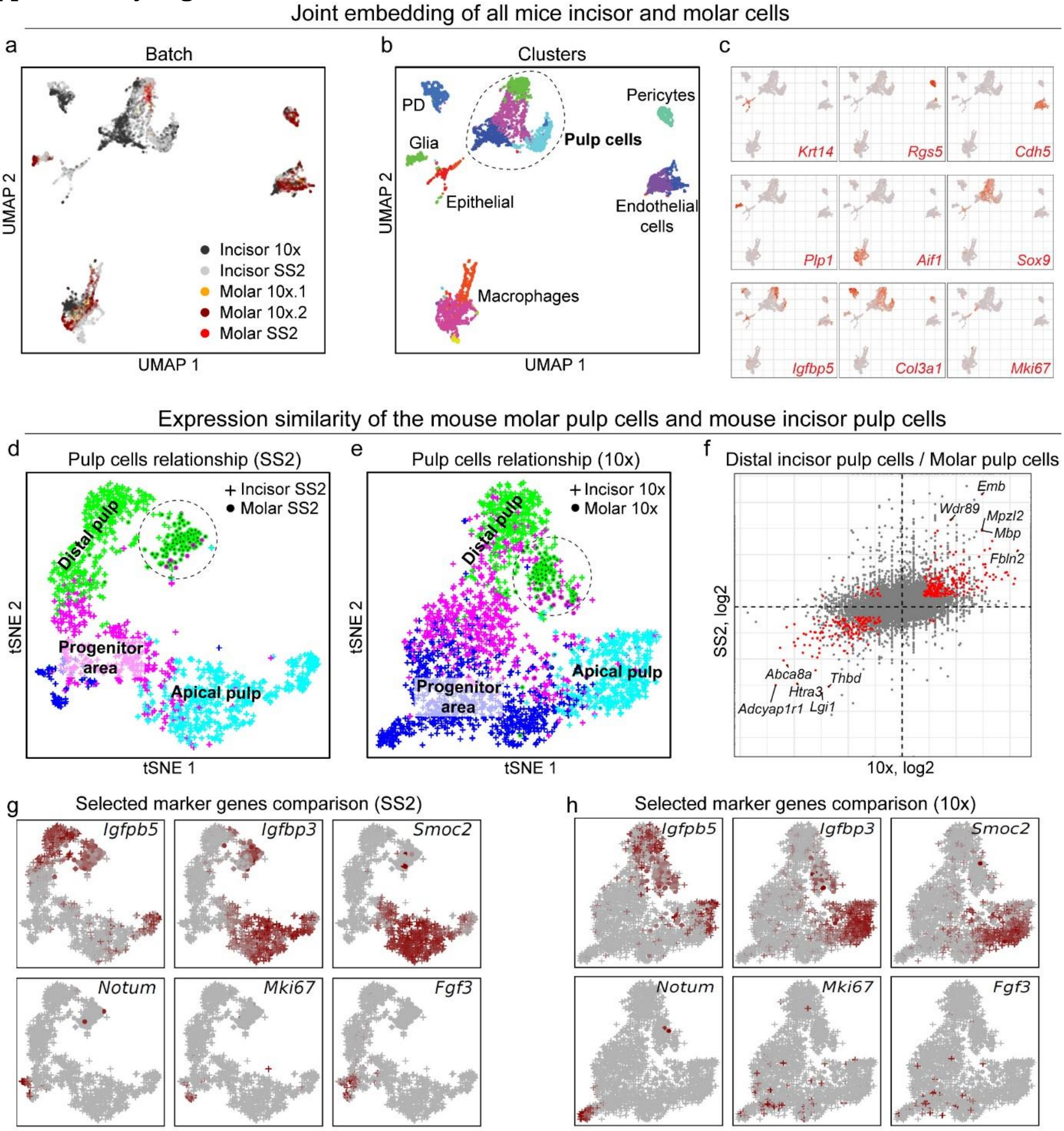

Supplementary Figure 4. Comparison of mouse incisor and molar teeth.

a) UMAP embedding of CONOS joint graph visualizes dental cell composition of 9164 cells across two incisor (4236 cells of 10x Chromium and 2889 cells of Smart-seq2) and three molar (1460 and 384 cells of 10x Chromium and 195 cells of Smart-seq2) mouse teeth datasets. Each dataset is composed of multiple teeth (for details please see materials and methods).

b) Leiden clustering (22 clusters) shows similarities and contrasts of dental cell states between incisor and molar. Colors of mesenchymal clusters match colors of incisor pulp subpopulations on Figure 3.

c) Expression of selected marker genes.

d, e) tSNE embedding of Smart-seq2 **(d)** or 10x Chromium **(e)** mesenchymal cells corroborates three-branch structure of mouse incisor mesenchyme and emphasize more homogeneous distal-like transcriptional state of mouse molar (dotted circle outline mouse molar pulp). Cell colors correspond to colors of clusters on panel b.

f) Differential expression analysis between distal incisor pulp and molar pulp is consistent between Smart-seq2 and 10x Chromium and reveals markers of molar pulp. X/Y axes show fold change of average gene expression levels between distal incisor and molar pulps, red color shows genes significantly differentially expressed in both platforms (at least two fold change and $p < 10^{-2}$, t-test).

Supplementary Figure 5

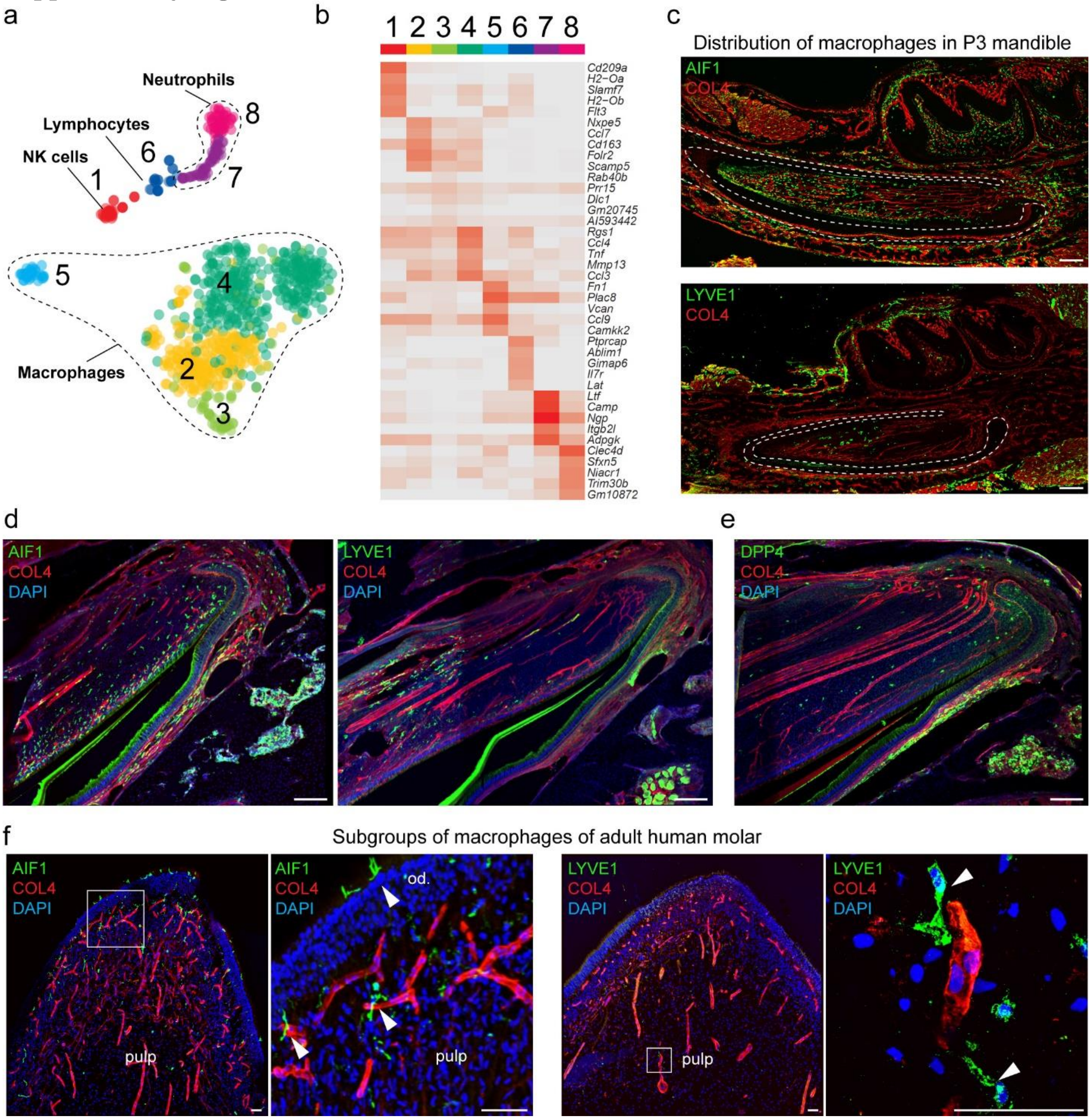

Supplementary Figure 5. Heterogeneity and signaling interactions in immune system of a tooth.

a,b) t-SNE dimensional reduction and corresponding heatmap with key differentially expressed genes visualizes 8 populations assigned as immune cells including lymphocytes, innate immune cells and macrophages. Markers of macrophage activation (*Nos1*, *Nos2*, *Nos3*, *Il4*, *Slc7a2*, *Arg1*, *Arg2*)were absent, suggesting that macrophages in non-damaged teeth remain inactive.

c) Cross-section through P3 mandible showing the differential spatial distribution of AIF1^{+} and LYVE1^{+} macrophages (immunohistochemistry). COL4 immunohistochemical staining visualize the blood vessels.

d) Immunohistochemistry-based validation of AIF1^{+}, LYVE1^{+} macrophages inside the mouse incisor. Together with the figure 5c these panels prove the different histological

positioning of two different kind of macrophages ($LYVE1^+$ and $LYVE1^-$). $AIF1^+$ macrophages are located in the whole incisor including apical pulp, cervical loop, odontoblast layer and distal pulp in contrast to $LYVE^+$ macrophages which mostly resides in the middle part of the pulp, but not inside the odontoblast layer. COL4 immunohistochemical staining visualize the blood vessels.

e) Immunohistochemistry-based validation of the NK-cells cluster using DPP4 marker. Position of $DPP4^+$ cells outside the blood vessels in the tooth was proved in 5 independent animals. COL4 immunohistochemical staining visualize the blood vessels.

f) Two different types of macrophages ($LYVE1^+$ and $LYVE1^-$) demonstrate spatially restricted distribution also in human molar (immunohistochemistry). Scale bars: 50 µm.

Supplementary table 1

General dataset

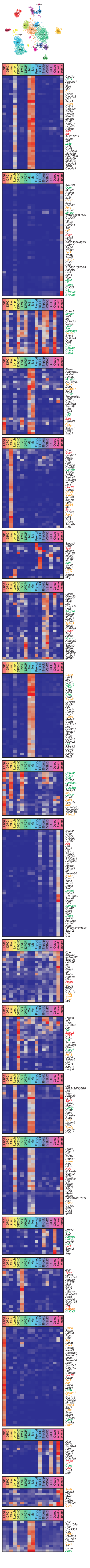

Epithelium

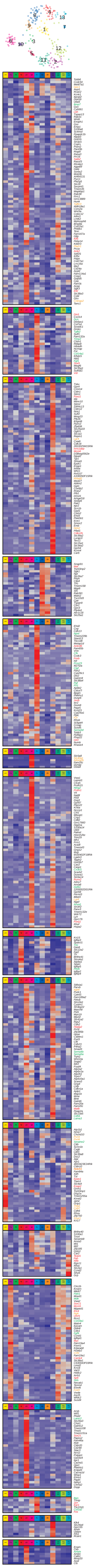

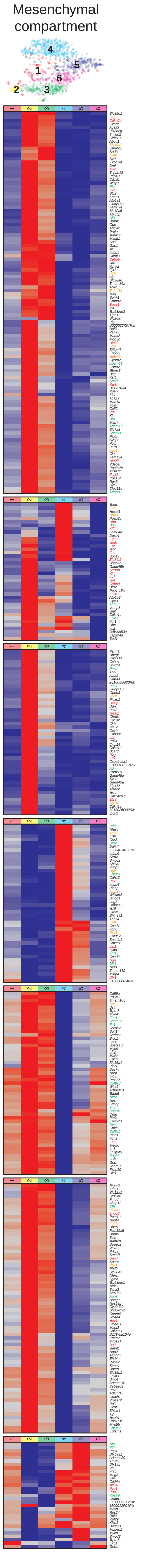
Mesenchymal
compartment
1
2
3
4
5
6

Immune cells

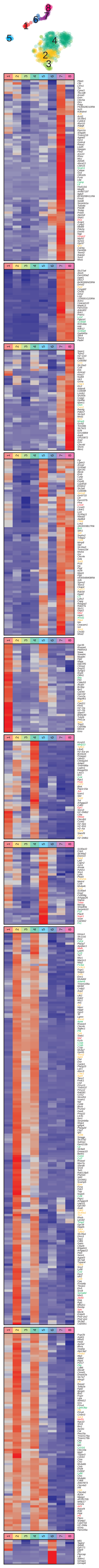

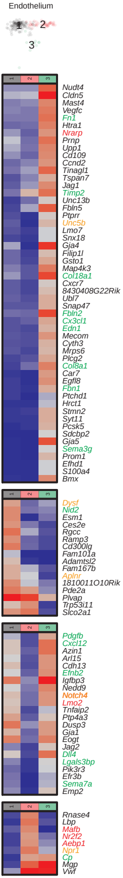
Endothelium
1
2
3
1 2 3
Nudt4
Cldn5
Mast4
Vegfc
Fn1
Htra1
Nrarp
Prnp
Upp1
Cd109
Ccnd2
Tinagl1
Tspan7
Jag1
Timp2
Unc13b
Fbln5
Ptprr
Unc5b
Lmo7
Snx18
Gja4
Filip1l
Gsto1
Map4k3
Col18a1
Cxcr7
8430408G22Rik
Ubl7
Snap47
Fbln2
Cx3cl1
Edn1
Mecom
Cyth3
Mrps6
Plcg2
Col8a1
Car7
Egfl8
Fbn1
Ptchd1
Hrct1
Stmn2
Syt11
Pcsk5
Sdcbp2
Gja5
Sema3g
Prom1
Efhd1
S100a4
Bmx
1 2 3
Dysf
Nid2
Esm1
Ces2e
Rgcc
Ramp3
Cd300lg
Fam101a
Adamtsl2
Fam167b
Aplnr
1810011O10Rik
Pde2a
Plvap
Trp53i11
Slco2a1
1 2 3
Pdgfb
Cxcl12
Azin1
Arl15
Cdh13
Efnb2
Igfbp3
Nedd9
Notch4
Lmo2
Tnfaip2
Ptp4a3
Dusp3
Gja1
Eogt
Jag2
Dll4
Lgals3bp
Pik3r3
Efr3b
Sema7a
Emp2
1 2 3
Rnase4
Lbp
Mafb
Nr2f2
Aebp1
Npr1
Cp
Mgp
Vwf

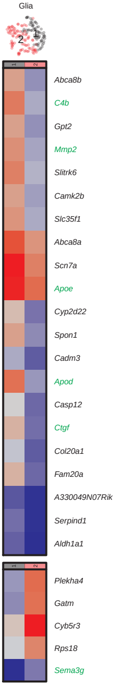
Glia
2
1
Abca8b
C4b
Gpt2
Mmp2
Slitrk6
Camk2b
Slc35f1
Abca8a
Scn7a
Apoe
Cyp2d22
Spon1
Cadm3
Apod
Casp12
Ctgf
Col20a1
Fam20a
A330049N07Rik
Serpind1
Aldh1a1
Plekha4
Gatm
Cyb5r3
Rps18
Sema3g

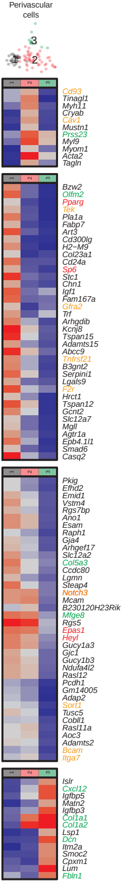
Perivascular
cells
1
2
3
Cd93
Tinagl1
Myh11
Cryab
Cav1
Mustn1
Prss23
Myl9
Myom1
Acta2
Tagln
Bzw2
Olfm2
Pparg
Tek
Pla1a
Fabp7
Art3
Cd300lg
H2−M9
Col23a1
Cd24a
Sp6
Stc1
Chn1
Igf1
Fam167a
Gfra2
Trf
Arhgdib
Kcnj8
Tspan15
Adamts15
Abcc9
Tnfrsf21
B3gnt2
Serpini1
Lgals9
F2r
Hrct1
Tspan12
Gcnt2
Slc12a7
Mgll
Agtr1a
Epb4.1l1
Smad6
Casq2
Pkig
Efhd2
Emid1
Vstm4
Rgs7bp
Ano1
Esam
Raph1
Gja4
Arhgef17
Slc12a2
Col5a3
Ccdc80
Lgmn
Steap4
Notch3
Mcam
B230120H23Rik
Mfge8
Rgs5
Epas1
Heyl
Gucy1a3
Gjc1
Gucy1b3
Ndufa4l2
Rasl12
Pcdh1
Gm14005
Adap2
Sort1
Tusc5
Cobll1
Rasl11a
Aoc3
Adamts2
Bcam
Itga7
Islr
Cxcl12
Igfbp5
Matn2
Igfbp3
Col1a1
Col1a2
Lsp1
Dcn
Itm2a
Smoc2
Cpxm1
Lum
Fbln1

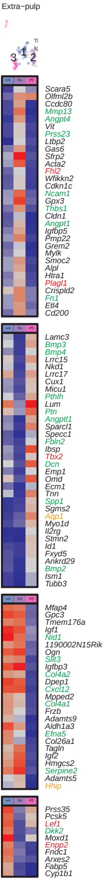

Extra−pulp
3
1
2
Scara5
Olfml2b
Ccdc80
Mmp13
Angpt4
Vit
Prss23
Ltbp2
Gas6
Sfrp2
Acta2
Fhl2
Wfikkn2
Cdkn1c
Ncam1
Gpx3
Thbs1
Cldn1
Angpt1
Igfbp5
Pmp22
Grem2
Mylk
Smoc2
Alpl
Htra1
Plagl1
Crispld2
Fn1
Etl4
Cd200
Lamc3
Bmp3
Bmp4
Lrrc15
Nkd1
Lrrc17
Cux1
Micu1
Pthlh
Lum
Ptn
Angptl1
Sparcl1
Specc1
Fbln2
Ibsp
Tbx2
Dcn
Emp1
Omd
Ecm1
Tnn
Spp1
Sgms2
Aqp1
Myo1d
Il2rg
Stmn2
Id1
Fxyd5
Ankrd29
Bmp2
Ism1
Tubb3
Mfap4
Gpc3
Tmem176a
Igf1
Nid1
1190002N15Rik
Ogn
Slit3
Igfbp3
Col4a2
Dpep1
Cxcl12
Mpped2
Col4a1
Frzb
Adamts9
Aldh1a3
Efna5
Col26a1
Tagln
Igf2
Hmgcs2
Serpine2
Adamts5
Hhip
Prss35
Pcsk5
Lef1
Dkk2
Moxd1
Enpp2
Fndc1
Arxes2
Fabp5
Cyp1b1

Supplementary table 2

	cluster: En	cluster: DF	cluster: Gli	cluster: Ly	cluster: DF	cluster: PV	cluster: DF	cluster: pa	cluster: pa
Clec7a	0.11	0.07	0.01	1.38	0.04	0.15	0.05	1.92	2.63
Cd14	0.43	0.33	0.02	1.28	0.3	0.29	0.23	2.62	3.07
Apobec1	0.25	0.26	0.63	1.6	0.2	0.21	0.11	2.42	3.15
Fyb	0.09	0.17	0.05	1.85	0.21	0.09	0.05	2.51	2.91
Plek	0.15	0.18	0.05	1.91	0.18	0.13	0.09	2.5	2.77
Il1b	0.21	0.19	0.04	1.31	0.16	0.16	0.06	1.01	2.71
Ccr1	0.1	0.11	0.02	0.82	0.07	0.08	0.06	2.08	2.37
Lpcat2	0.16	0.15	1.08	1.2	0.16	0.07	0.08	2.68	3.08
Clec4a2	0.05	0.08	0.02	1.18	0.05	0.06	0.03	2.82	2.67
Lst1	0.07	0.13	0.05	1.6	0.13	0.16	0.06	2.72	2.62
Fcgr3	0.26	0.22	0.03	1.79	0.16	0.1	0.15	3.56	3.47
C5ar1	0.09	0.08	0.01	0.36	0.03	0.13	0.04	2.47	2.1
Cyth4	0.72	0.11	0.03	2.3	0.08	0.16	0.07	2.81	2.92
Cd300a	0.09	0.11	0.03	1.87	0.07	0.21	0.04	2.65	2.72
Evi2a	0.06	0.11	0.1	2.01	0.09	0.09	0.06	2.5	2.6
Lrrc33	0.99	0.06	0.06	2.46	0.14	0.12	0.08	2.7	2.4
Neurl3	0.2	0.1	0.07	1.88	0.09	0.04	0.04	2.3	2.7
Mpeg1	0.18	0.17	0.03	2.18	0.08	0.12	0.1	3.01	3.51
Cd68	0.29	0.22	0.09	1.96	0.18	0.17	0.18	3.38	3.35
Wfdc17	0.1	0.12	0.04	1.79	0.11	0.1	0.08	3.17	2.43
Fcgr2b	0.24	0.16	0.84	2.19	0.18	0.16	0.12	3.26	3.26
Rgs10	0.16	0.24	0.38	1.57	0.14	0.59	0.07	2.94	2.97
Atf3	0.49	0.23	0.44	1.78	0.19	0.63	0.12	2.03	3.12
Irf5	0.13	0.15	0.04	2.09	0.1	0.22	0.1	2.3	2.65
AF251705	0.11	0.08	0.01	1.65	0.07	0.09	0.04	2.41	3.08
Il10ra	0.1	0.09	0.03	1.9	0.05	0.2	0.04	2.58	2.9
Irf8	0.18	0.08	0.19	1.49	0.07	0.08	0.05	2.51	2.8
Ly86	0.09	0.1	0.01	2.07	0.08	0.12	0.07	3.16	3.29
Pld4	0.15	0.11	0.04	2.49	0.15	0.14	0.08	3.05	3.11
Cd48	0.08	0.08	0.01	2.44	0.06	0.09	0.04	2.6	2.7
H2-DMa	0.33	0.31	0.09	2.22	0.29	0.18	0.24	2.56	3.14
Ms4a6d	0.17	0.11	0.01	1.04	0.02	0.03	0.04	2.67	2.55
AI607873	0.1	0.09	0.01	1.26	0.03	0.08	0.02	2.63	2.81
Ms4a6b	0.09	0.12	0	1.81	0.04	0.06	0.03	2.91	2.85
Ms4a6c	0.07	0.08	0.02	1.94	0.03	0.03	0.04	2.64	2.64
Clec4a3	0.13	0.13	0.02	1.55	0.06	0.08	0.05	2.94	2.96
Tifab	0.06	0.02	0.03	1.97	0.02	0.02	0.05	2.24	2.63
Clec4a1	0.06	0.1	0.02	1.48	0.02	0.05	0.05	2.7	2.31
Adam8	0.08	0.21	0.03	0.61	0.12	0.1	0.09	0.15	0.18
Osm	0.03	0.06	0.01	0.86	0.05	0.04	0.02	0.58	1.03
Mmp8	0.07	0.13	0.02	0.54	0.03	0.02	0.01	0.04	0.05
Retnlg	0.16	0.11	0.05	0.43	0.05	0.21	0.04	0.12	0.09
Slfn1	0.01	0.03	0.02	0.89	0.01	0.01	0	0.17	0.2
Il1f9	0.02	0.01	0.01	0.06	0	0.03	0.01	0.03	0.01
Cxcr2	0.04	0.03	0	0.16	0.01	0.01	0.01	0.01	0.01
Il1r2	0	0.08	0	0.28	0.02	0.05	0.01	0.05	0.04
Clec4d	0.02	0.03	0.01	0.41	0.01	0.01	0.01	0.29	0.19
Fpr1	0.02	0.03	0	0.03	0.01	0.01	0.01	0.72	0.22
Slc2a3	0.11	0.29	0.19	0.47	0.23	0.28	0.35	0.2	0.21
Mmp9	0.07	0.08	0.07	0.2	0.04	0.25	0.09	0.81	0.18
1810033B	0.02	0	0.02	1.47	0	0.02	0	0.03	0.05

Cd300lf	0.01	0.02	0.01	0.79	0.01	0.02	0	0.28	0.51
Fgr	0.02	0.05	0.03	1.58	0.01	0.02	0	0.13	0.25
Ifitm6	0.05	0.02	0	1.57	0.01	0.03	0	0.32	0.1
Pstpip1	0.04	0.07	0	1.78	0.06	0.02	0.02	0.48	0.42
Slpi	0.03	0.06	0.03	0.8	0.06	0.02	0	0.01	0.02
Sell	0.04	0.05	0.01	1.49	0.01	0.01	0.01	0.02	0
Hp	0.03	0.05	0.02	1.24	0.02	0.05	0.01	0.03	0.02
Nfe2	0.02	0.15	0	1.06	0.04	0.03	0.01	0	0.03
Ly6c2	0.47	0.06	0.01	1.6	0	0.04	0.01	0.03	0.05
Gda	0.11	0.13	0.01	1.11	0.02	0.2	0.07	0.02	0.07
B430306N	0.04	0.06	0.04	0.38	0.01	0.01	0.01	0.47	0.22
Pram1	0.05	0.08	0.04	0.61	0.05	0.08	0.13	0.18	0.14
Padi4	0.03	0.06	0.03	0.26	0.05	0.01	0.01	0.02	0.01
Trem3	0.01	0.02	0	0.51	0	0	0	0.01	0.03
Fpr2	0.01	0.02	0	0.19	0	0	0	0.31	0.22
Trem1	0.01	0.04	0.02	0.29	0.01	0	0.02	0	0.02
Alox5	0.02	0.1	0.02	0.3	0.02	0.01	0.02	0.87	0.44
Cd177	0	0.06	0.01	0.6	0.01	0.03	0.02	0.02	0.02
Chi3l1	0.03	0.12	0.02	0.06	0.25	0.01	0.1	0.05	0.01
Hdc	0.08	0.09	0	0.54	0.02	0.09	0.02	0.02	0.03
1100001G	0.02	0.02	0.01	0.15	0.01	0.02	0.01	0.01	0.03
Pglyrp1	0.15	0.02	0.03	0.67	0.03	0.02	0.01	0.01	0.02
Lrg1	1.86	0.06	0.02	0.1	0.03	0.01	0.01	0.04	0.03
Slfn4	0.45	0.09	0.02	0.28	0.01	0.03	0.01	0.1	0.04
Ngp	0.28	0.19	0.04	0.81	0.11	0.23	0.08	0.17	0.19
Lcn2	0.17	0.11	0.04	0.79	0.08	0.12	0.05	0.08	0.11
Ltf	0.15	0.09	0.02	0.39	0.06	0.15	0.03	0.11	0.1
Camp	0.11	0.09	0.04	0.42	0.05	0.11	0.04	0.07	0.08
Chi3l3	0.11	0.12	0.02	1.14	0.05	0.11	0.02	0.04	0.07
C3	0.02	0.08	0.03	1.08	0.02	0.05	0.01	0.23	0.15
S100a9	0.35	0.27	0.11	0.97	0.15	0.34	0.1	0.3	0.21
S100a8	0.43	0.3	0.13	1.34	0.17	0.37	0.12	0.32	0.3
Cdh11	0.27	2.77	0.18	0.25	3.1	1.57	2.81	0.27	0.24
Mmp2	0.56	2.81	2.01	0.29	2.77	1.67	2.88	0.71	1.09
Wif1	0.48	1.96	0.26	0.38	2.88	0.33	2.14	0.49	0.43
Islr	0.21	2.63	0.95	0.11	3.37	0.96	2.68	0.3	0.2
Colec12	0.46	2.6	0.14	0.15	3.18	1.53	3.09	0.57	0.34
Cpxm1	0.19	1.7	0.25	0.18	1.46	0.67	2.97	0.18	0.17
Fbln1	0.21	2.02	0.33	0.18	2.98	1.08	2.92	0.32	0.16
Ptn	1.05	2.64	1.22	0.17	2.28	0.43	3.12	0.25	0.24
Serping1	0.23	2.07	0.43	0.13	2.79	2.87	2.62	0.19	0.12
Pdgfrb	0.18	1.84	0.19	0.21	2.51	3.4	2.12	0.2	0.17
S1pr3	0.29	2.91	0.68	0.35	3.52	2.91	2.91	0.42	0.29
Col12a1	0.21	2.52	1.64	0.26	2.29	1.04	0.93	0.29	0.26
Omd	0.19	2.23	0.11	0.25	2.2	0.33	0.93	0.25	0.19
Lum	0.79	3.53	0.31	0.63	4.24	1.07	2.1	1.25	0.93
Dcn	0.25	2.85	0.17	0.17	3.44	0.68	1.56	0.5	0.38
Col1a2	0.57	3.14	2.78	0.75	3.46	2.11	2.72	0.82	0.77
Col1a1	0.69	3.1	1.41	0.86	3	1.93	2.43	1.09	1.03
Col3a1	0.69	3.22	2.42	0.77	3.41	3.5	2.6	0.72	0.58
Gatm	0.51	0.16	1.42	0.76	0.09	0.15	0.12	2.89	3.14
Arhgap19	0.19	0.29	0.34	0.53	0.09	0.01	0.1	2.58	2.24

Vcam1	0.73	0.31	0.31	0.16	0.19	0.74	0.22	3.15	2.83
Pla2g7	0.17	0.58	0.05	0.98	0.35	0.45	1.31	3.05	3.15
Mmp13	0.11	0.14	0.1	0.23	0.17	0.21	0.1	1.05	2.2
H2-DMb1	0.34	0.66	0.04	1.66	1.03	0.52	0.52	1.73	2.83
Tgfbr1	0.75	0.74	0.4	0.79	0.76	0.52	0.63	2.21	3.04
Dab2	1.67	0.6	0.06	0.7	0.63	0.53	0.56	3.5	3.24
Slc40a1	0.45	0.44	0.06	0.14	1.49	0.26	0.29	3.37	2.28
Folr2	0.08	0.11	0.03	0.1	0.09	0.08	0.07	2.49	0.71
F13a1	0.08	0.18	0.05	1.24	0.12	0.06	0.14	2.92	1.04
Tmem106a	0.41	0.26	0.08	0.98	0.27	0.22	0.26	2.57	2.12
Cbr2	0.2	0.11	0.01	0.1	0.09	0.07	0.06	2.82	1.39
Casp1	0.49	0.12	0.07	1.33	0.18	0.07	0.05	1.8	1.57
Bcl2a1b	0.02	0.01	0	0.5	0.01	0.02	0.01	1.31	2.32
Slamf9	0.2	0.2	0.06	1.1	0.17	0.25	0.18	1.45	2.67
Cd83	0.18	0.29	0	0.92	0.43	0.2	0.3	2.03	2.96
Rgs1	0.2	0.19	0.02	0.99	0.11	0.17	0.08	1.46	2.98
Ccl4	0.17	0.18	0	0.79	0.13	0.15	0.06	1.3	2.57
Ccl3	0.1	0.09	0	0.67	0.13	0.06	0.04	1.67	2.75
Ets1	2.96	0.88	2.06	0.72	1.13	2.22	0.45	0.59	0.58
Ptp4a3	2.06	0.91	0.29	1.29	1.11	3.16	0.6	1.63	1.38
Nrp1	3.43	0.72	0.84	0.91	0.44	2.79	0.59	2.9	2.64
Cd93	3.15	0.14	0.04	0.82	0.07	1.81	0.05	2.26	2.3
Entpd1	3.03	0.24	0.02	0.91	0.11	1.2	0.12	2.63	2.68
Cd38	2.38	0.28	0.04	0.26	0.6	0.34	0.12	2.83	1.78
Stab1	2.32	0.23	0.05	0.24	0.11	0.1	0.08	3.33	2.96
Cnp	0.58	0.84	3.19	1.48	0.96	0.32	0.5	1.46	1.23
Gas7	0.48	1.42	3.41	1.13	1.77	0.38	0.27	1.21	0.91
Plekhb1	0.11	1.51	3.32	0.07	0.96	0.14	0.6	0.1	0.09
Fndc1	0.09	1.35	2.97	0.09	1.88	0.2	1.06	0.13	0.06
Dmd	0.38	0.23	2.71	0.12	0.07	2.04	0.3	0.04	0.06
Aatk	0.19	0.21	2.82	0.05	0.6	0.33	0.4	0.29	0.22
Gpm6b	0.11	0.72	3.3	0.08	0.77	0.26	0.6	0.09	0.05
Cyp2j9	0.31	0.55	2.43	0.06	0.64	0.18	0.46	0.04	0.03
Adam23	0.03	0.49	2.59	0.18	1	0.07	0.12	0.04	0.07
S100b	0.04	0.07	2.39	0.01	0.04	0.06	0.05	0.05	0.02
Fabp7	0.02	0.11	2.34	0.03	0.18	0.97	0.06	0.04	0.06
Atp1a2	0.02	0.31	2.41	0.03	0.39	0.8	0.27	0.02	0.04
Col28a1	0.12	0.1	2.51	0.01	0.02	0.05	0.02	0.01	0.02
Kcna2	0	0.1	2.69	0	0.04	0.08	0.03	0.03	0.04
Lgi4	0.02	0.27	2.82	0	0.17	0.09	0.02	0.03	0.06
Sox10	0.01	0.16	3.24	0.01	0.41	0.05	0.06	0.03	0.01
Cdh19	0.02	0.35	2.75	0	0.48	0.03	0.07	0.03	0.02
Gfra3	0.03	0.06	2.52	0	0.01	0.04	0.02	0.01	0
Gpr37l1	0	0.02	2.22	0	0.01	0.03	0.01	0.01	0.01
Kcna6	0.22	0.13	2.46	0	0	0.03	0.16	0	0.02
Scn7a	0.04	0.12	3.24	0.01	0.01	0.16	0.13	0.03	0.01
Egfl8	0.35	0.06	2.5	0.14	0.04	0.05	0.05	0.05	0.08
Erbb3	0.04	0.16	2.26	0.04	0.09	0.08	0.02	0.12	0.11
Mal	0.4	0.04	2.23	0.03	0.01	0.05	0.01	0.02	0.01
Esr1	0.16	0.24	2.33	0.15	0.31	0.33	0.09	0.25	0.12
L1cam	0.05	0.05	2.78	0.48	0.02	0.05	0.02	0.01	0.03
Ptprz1	0.01	0.5	2.65	0.05	0.05	0.11	0.12	0.03	0.04

Plp1	0.08	0.33	3.85	0.03	0.15	0.15	0.23	0.09	0.05
Art3	0.63	0.13	3.21	0.02	0.02	1.44	0.05	0.02	0.03
Cryab	0.02	0.06	3.16	0.09	0.05	1.38	0.05	0.03	0.09
Abca8a	0.04	0.25	2.89	0.03	0.13	0.63	0.32	0.08	0.02
Chl1	0.02	0.12	3.01	0	0.04	0.05	0.04	0.03	0.02
Smpd3	0.09	0.93	0.15	0.11	0.1	0.09	0.17	0.17	0.13
Car2	0.48	0.97	0.02	0.43	0.75	0.53	0.1	0.11	0.07
Sp6	0.13	1.46	0.07	0.16	1.24	1.08	0.45	0.12	0.14
Moxd1	0.13	0.95	0.69	0.07	0.73	0.08	1.32	0.11	0.05
Cldn10	0.08	0.73	0.04	0.14	2.74	0.25	0.24	0.19	0.08
Igfbp2	0.1	0.06	0.02	0.01	0.08	0.22	0.02	0.03	0.02
Epcam	0.06	0.12	0.03	0.07	0	0.03	0.02	0.03	0.04
Krt17	0.05	0.09	0.08	0.07	0.05	0.05	0.03	0.02	0.03
Shh	0.11	0.16	0.19	0.27	0.04	0.12	0.07	0.11	0.06
Vwa2	0.03	0.05	0.08	0.05	0.02	0.04	0.01	0.04	0.02
Fras1	0.08	0.53	0.16	0.07	0.78	0.37	0.07	0.08	0.12
Ccr2	0.02	0.05	0	2.29	0	0.07	0.02	0.11	0.72
Itgb7	0.04	0.07	0.03	2.24	0.03	0.03	0.09	0.03	0.06
Napsa	0.02	0.05	0.01	2.57	0.01	0	0.01	0	0.09
Mbp	0.06	0.33	2.58	1.64	0.05	0.12	0.05	0.3	0.53
Postn	0.54	1.05	3.25	0.39	1.06	2.6	1.09	0.79	0.57
Olfml2b	0.32	0.4	1.44	0.17	1.67	2.35	0.2	0.15	0.2
Spon1	0.15	0.6	1.82	0.29	0.28	1.23	1.1	0.07	0.11
Myl9	0.17	1.56	1.28	0.28	1.75	2.42	1.76	0.28	0.18
Lsp1	0.13	1.14	0.1	3.22	0.96	0.47	2.06	0.23	0.53
Crispld2	0.09	0.6	0.34	0.17	1.42	1.23	1.36	0.14	0.12
Ogn	0.13	0.84	0.07	0.16	2.07	0.74	0.52	0.13	0.07
Angpt4	0.13	1.57	0.09	0.13	2.5	0.26	0.94	0.16	0.1
Scara5	0.02	0.77	0.05	0.01	1.79	0.91	0.64	0.12	0.1
Igfbp5	0.55	0.72	0.26	0.38	3.51	1.38	0.66	0.77	0.38
Dpep1	0.06	0.19	0.07	0.09	0.26	0.21	0.77	0.05	0.03
Grem2	0	0.05	0.03	0.02	0.03	0.31	1.07	0.02	0.01
Epha3	0.03	0.11	0.09	0.03	0.03	0.6	0.49	0.03	0.02
Thsd4	0.02	0.16	0.48	0.13	0.21	0.56	0.61	0.04	0.05
Col26a1	0.04	0.96	0.09	0.08	1.06	0.24	0.16	0.05	0.04
Foxf2	0.03	1.12	0.05	0.09	0.86	0.79	1.77	0.11	0.07
Tagln	0.09	0.1	0.88	0.17	0.15	1.55	0.14	0.13	0.09
Acta2	0.16	0.28	0.15	0.4	0.27	2.35	0.23	0.28	0.17
Ncam1	0.2	1.48	2.71	0.27	1.32	0.83	2.22	0.22	0.11
Hmgcs2	0.12	0.46	1.17	0.09	0.82	0.49	2.33	0.13	0.08
Sfrp2	0.42	1.11	0.55	0.67	0.96	0.81	3.49	0.65	0.36
Smoc2	0.42	0.72	0.46	0.31	0.72	0.66	4.07	0.53	0.28
Mfap4	0.17	1.87	0.15	0.18	0.89	0.35	3.51	0.14	0.17
Shisa2	0.12	1.16	0.35	0.13	1.28	0.19	2.79	0.09	0.07
Wfikkn2	0.09	0.54	0.08	0.07	0.3	0.14	2.54	0.09	0.08
Crabp1	0.06	1.95	0.11	0.11	1.51	0.11	2.49	0.44	0.16
Igfbp3	2.58	0.91	1.55	0.27	1.26	1.25	3.73	0.7	0.46
Cx3cr1	0.13	0.16	0.03	1.05	0.14	0.17	0.12	3.21	3.32
Emr1	0.25	0.2	0.03	1.34	0.12	0.14	0.17	3.53	3.53
Mrc1	0.28	0.26	0.12	0.48	0.17	0.24	0.25	3.66	3.23
Hpgd	0.17	0.18	0.02	0.65	0.11	0.29	0.13	3.16	2.72
Cxcl16	0.44	0.21	0.03	0.84	0.24	0.16	0.11	2.68	3.35

C1qa	0.37	0.35	0.07	0.31	0.25	0.3	0.21	3.8	3.83
C1qc	0.38	0.34	0.11	0.32	0.31	0.34	0.26	3.97	3.93
Fcrls	0.3	0.28	0.03	0.21	0.22	0.2	0.22	3.65	3.37
C1qb	0.63	0.61	0.1	0.49	0.44	0.53	0.52	4.13	4.11
Lilra5	0.09	0.06	0.01	0.1	0.04	0.11	0.03	2.09	2.79
Trem2	0.05	0.07	0.01	0.3	0.02	0.06	0.04	2.55	2.62
P2ry13	0.05	0.06	0.02	0.17	0.06	0.05	0.04	2.38	2.14
Gpr34	0.15	0.11	0.01	0.46	0.05	0.18	0.08	2.64	2.82
Aif1	0.11	0.12	0.01	0.77	0.08	0.08	0.05	3.09	3.09
Hpgds	0.1	0.08	0.04	0.43	0.06	0.04	0.06	2.87	2.69
Clec4n	0.09	0.13	0.01	0.28	0.06	0.07	0.07	3.14	2.86
Fcgr1	0.11	0.09	0	0.77	0.07	0.03	0.03	2.9	3.03
C3ar1	0.13	0.1	0.02	0.1	0.11	0.08	0.1	2.86	3.21
Ms4a7	0.18	0.19	0.02	0.23	0.1	0.15	0.1	3.36	3.55
Gpr65	0.09	0.11	0.01	0.96	0.09	0.08	0.07	2.74	2.9
Slc11a1	0.1	0.12	0.03	0.76	0.06	0.33	0.06	2.67	2.86
Lair1	0.06	0.1	0.04	0.63	0.03	0.09	0.05	2.37	2.9
Slco2b1	0.77	0.14	0.01	0.04	0.06	0.18	0.05	2.72	2.57
Tbxas1	0.09	0.13	0.02	0.7	0.05	0.06	0.06	2.48	2.24
Msr1	0.05	0.09	0.06	0.56	0.02	0.06	0.03	2.55	2.26
Cd86	0.07	0.07	0	0.81	0.06	0.06	0.03	2.5	2.48
Siglec1	0.1	0.13	0.05	0.23	0.11	0.15	0.12	2.93	2.41
Prune2	0.03	0.24	0.04	0.09	0.14	0.06	0.06	2.55	1.77
Pf4	0.36	0.15	0.02	0.15	0.09	0.15	0.08	3.31	2.62
P2ry12	0.06	0.11	0.15	0.2	0.09	0.05	0.03	2.53	2.4
Slc9a9	0.09	0.07	0.02	0.5	0.08	0.12	0.07	2.55	1.99
P2ry6	0.23	0.11	0.03	1.18	0.08	0.19	0.05	2.77	2.79
Adap2	0.05	0.08	0.01	0.41	0.02	1.56	0.02	2.18	2.51
Fgd2	0.08	0.08	0.02	1.15	0.04	0.04	0.03	2.34	2.48
Col4a2	3.32	0.8	2.51	0.34	1.11	3.32	0.57	0.21	0.26
Col4a1	3.44	0.72	2.72	0.12	1.03	3.57	0.45	0.21	0.32
Nid1	2.56	1.93	2.83	0.27	1.67	2.68	1.74	0.17	0.1
Cd200	3.19	0.6	2.54	0.26	0.89	2.19	0.93	0.12	0.13
Serpine2	0.35	0.79	3.46	0.28	0.64	3.26	1.86	0.19	0.14
Mcam	2.53	0.12	3.19	0.08	0.04	2.98	0.09	0.07	0.1
Col18a1	1.34	0.17	3.35	0.02	0.12	2.36	0.1	0.06	0.07
Tm4sf1	3.2	0.17	2.32	0.06	0.05	2.78	0.14	0.07	0.1
Itga1	1.58	0.21	2.82	0.23	0.14	2.66	0.09	0.03	0.05
Sorbs1	1.64	1.1	3.25	0.24	0.92	2.03	0.6	0.12	0.12
Crip2	3.16	0.28	2.87	0.08	0.43	2.75	0.28	0.1	0.09
Aqp1	2.91	1.5	0.38	0.23	2.31	0.62	0.24	0.21	0.24
Ppap2a	3.26	0.66	1.62	0.22	0.38	0.75	0.45	0.1	0.07
Eng	3.67	0.94	2.2	0.47	1.25	2.1	0.9	0.67	0.76
Slc9a3r2	3.14	0.66	1.6	0.07	0.98	1.55	0.49	0.11	0.15
Tmem204	3.01	0.41	1.01	0.15	0.83	1.69	0.79	0.2	0.16
Tspan18	2.75	0.66	0.15	0.06	1.97	0.32	0.47	0.14	0.1
Ramp2	3	0.89	0.3	0.09	0.67	0.52	0.82	0.06	0.03
Nipal2	0.04	0.47	0.05	0.04	0.36	0.21	0.59	0.03	0.02
Gng4	0.05	0.11	0.04	0.02	0.04	0.07	0.39	0.02	0.04
Cxadr	0.1	0.25	0.02	0.07	1.5	0.06	0.2	0.05	0.06
Cyb561	0.8	0.05	0.01	0.09	0.01	0.02	0.01	0.01	0.01
Lamb3	0.23	0.14	0.06	0.17	0.09	0.12	0.22	0.15	0.11

Klf5	0.03	0.09	0.13	0.02	0.04	0.14	0.09	0.03	0.02
Sfn	0.2	0.03	0.02	0.12	0.01	0.03	0.04	0.03	0.08
Pkp1	0.04	0.11	0.06	0.04	0.01	0.02	0	0	0.04
Dsc2	0.05	0.06	0	0.02	0.01	0.02	0.01	0.02	0
Trim29	0.04	0.1	0.01	0.02	0.06	0	0.04	0.02	0.04
Gpr115	0.04	0.12	0.03	0.06	0.18	0.03	0.05	0.04	0.02
S100a14	0.03	0.01	0.01	0	0	0	0.01	0	0
Serpinb5	0.01	0.06	0	0	0.01	0	0.02	0.01	0.05
Lad1	0.08	0.04	0.05	0.09	0.02	0.05	0.02	0.02	0.03
Zfp185	0.04	0.08	0.01	0.01	0.03	0.03	0.08	0.01	0.02
Mboat1	0.05	0.14	0.1	0.28	0.03	0.24	0.05	0.27	0.06
Mt2	0.08	0.04	0.75	0.25	0.03	0.4	0.03	0.63	0.37
Serpinb8	0.14	0.06	0.17	0.26	0.06	0.02	0.03	1.2	1.1
Ntrk2	0.03	0.33	1.05	0	0.1	0.27	0.17	0.06	0.03
Ckmt1	0.12	0.05	0.05	0.11	0.01	0.07	0.02	0.05	0.1
Tns4	0.03	0.09	0.03	0.29	0.01	0	0.03	0.27	0.42
Kcnk1	0.07	0.12	0.07	0.1	0.39	0.06	0.13	0.04	0.04
Dmkn	0.04	0.17	0.02	0.2	0.73	0.01	0.02	0.08	0.09
Amtn	0.12	0.12	0.04	0.15	0.07	0.08	0.05	0.12	0.13
Lama3	0.78	0.17	0.08	0	0.06	0.03	0.02	0.03	0.05
Palmd	1.56	0.13	0.06	0.02	0.15	0.02	0.01	0.01	0.05
Gm17660	0.14	0.07	0.01	0.07	0.06	0.06	0.09	0.1	0.06
Anxa8	0.07	0.08	0.01	0.1	0.03	0.07	0.05	0.05	0.07
Odam	0.44	0.21	0.06	0.3	0.22	0.12	0.34	0.36	0.38
Flt3l	0.24	0.07	0.31	0.32	0.16	0.24	0.11	0.25	0.21
Sema3d	0.08	0.2	0.39	0	0.07	0.08	0.02	0.02	0.02
Gpr87	0.01	0.02	0	0	0	0	0.01	0.01	0.01
Dsg3	0.02	0.03	0.02	0	0	0	0	0	0.01
Ngef	0	0.07	0.02	0	0.03	0.02	0	0.02	0.01
Defb1	0.01	0.01	0.02	0	0	0	0.01	0.01	0.01
Wdr72	0.01	0.06	0	0.02	0.02	0.06	0.01	0.02	0.04
Fam25c	0.02	0	0	0	0	0	0	0	0
Slc5a8	0.01	0.04	0	0	0.01	0	0.01	0	0.01
Nat8l	0.01	0.05	0.08	0.34	0.02	0.05	0.02	0.03	0.08
2200002D	0.26	0.02	0.09	0.05	0.01	0.05	0.04	0.03	0.06
Sh3rf2	0.06	0.1	0.05	0.02	0.04	0.02	0.09	0.03	0.01
Cftr	0.04	0.05	0.03	0	0.02	0	0.01	0.01	0.01
Ggt1	0.07	0.04	0.03	0.03	0.03	0.01	0.04	0.06	0.02
Cpe	0.55	1.28	0.18	0.22	1.17	3.32	2.19	0.2	0.2
Scara3	0.26	2.51	0.09	0.22	3.49	1.85	1.01	0.53	0.19
Kidins220	1.29	2.17	1.12	1.17	2.23	1.24	1.62	1.09	1.07
Sparcl1	3.31	1.09	0.92	0.26	0.48	3.43	0.54	0.25	0.3
Sorbs2	1.71	1.74	1.82	0.06	2.53	1.42	0.26	0.19	0.11
Id4	0.16	1.13	0.5	0.06	2.36	1.32	0.31	0.18	0.11
Clu	1.96	1.33	0.57	0.41	2.83	0.42	0.49	0.43	0.39
Gsta4	0.08	1.87	0.18	0.11	1.9	0.82	2.01	0.3	0.14
Ide	0.77	0.81	0.52	0.91	1.03	0.74	0.69	0.79	0.64
Ambn	0.42	1.83	0.48	0.61	2.55	0.45	1.38	0.63	0.6
Higd1a	0.51	0.91	0.56	0.85	0.66	0.74	0.41	0.49	0.45
Gss	0.43	0.56	0.38	0.27	0.43	0.27	0.42	0.43	0.38
Foxq1	0.04	0.24	0	0.01	0.19	0.06	0.36	0.05	0.03
Lgr6	0.05	0.69	0.45	0.02	0.4	0.07	0.29	0.02	0.04

Btbd3	1.39	0.52	0.97	0.15	0.4	1.05	0.5	0.32	0.32
Avpi1	0.53	0.88	0.16	0.57	1.53	0.62	0.57	0.47	0.44
Cdkn1a	1.02	0.22	1.21	1.09	0.32	1.4	0.05	0.62	1.14
Gpc1	0.09	0.5	1.71	0.28	0.45	1.23	0.28	0.07	0.05
Itga6	1.68	0.17	1.86	0.15	0.05	0.44	0.08	0.59	0.94
F11r	2.91	0.07	0.02	0.73	0.08	0.59	0.05	0.51	1.64
Mt1	0.78	0.7	1.68	1.26	0.74	1.36	0.82	2.81	2.73
Olfml3	0.26	2.48	1.06	0.26	3.75	0.9	2.35	1.24	2.22
Igf1	0.36	1.31	0.16	0.08	1.38	1.74	0.81	2.9	1.73
Gpx3	0.52	1.41	0.28	0.94	3.54	0.97	2.46	2.71	2.09
Slc20a2	0.57	2.23	0.64	0.53	3.67	0.96	1.03	0.78	0.41
Alpl	0.29	1.51	0.12	0.37	3.37	1.37	0.9	0.52	0.3
Tnc	0.18	2.85	0.81	0.24	3.69	0.31	0.38	0.41	0.24
Enpp2	0.82	2.86	0.31	0.34	3.84	1.15	1.46	0.6	0.26
Hey1	0.46	1.77	0.14	0.13	2.96	1.35	0.42	0.53	0.27
Chka	0.54	2.76	0.76	0.59	3.33	0.89	1.38	1.03	1.36
Cfh	0.62	2.11	0.12	0.69	3.17	3.06	0.3	2.18	2.01
Scube1	0.46	2.31	0.06	0.07	2.06	0.16	2.04	0.12	0.1
Col23a1	0.15	1.99	0.21	0.15	2.76	0.8	1.13	0.1	0.1
Olfml1	0.15	2.71	0.29	0.14	3.26	0.16	1.9	0.22	0.22
Rspo4	0.28	3.12	0.16	0.3	3.01	0.38	2	0.61	0.39
Ifitm1	0.13	2.53	0.08	0.63	2.98	1.55	2.1	0.67	0.3
Fgfr3	0.59	2.18	0.05	0.08	2.95	0.53	1.03	0.15	0.07
Fmod	0.41	2.91	0.25	0.38	3.97	0.83	0.85	0.7	0.48
St8sia6	0.23	1.48	0	0.43	2.58	0.06	0.1	0.08	0.09
Stc2	0.07	1.48	0.06	0.1	2.53	0.15	1	0.1	0.05
Car12	0.06	1.89	0.05	0.16	3.08	0.07	0.11	0.09	0.06
Kcnj15	0.27	1.68	0.06	0.1	2.83	0.09	0.09	0.15	0.06
Enpp6	0.79	1.85	0.03	0.12	2.98	0.12	0.85	0.16	0.09
Lyn	1.83	0.23	0.3	2.74	0.13	0.58	0.12	2.99	3.16
4632428N	0.76	0.21	0.12	2.13	0.14	0.32	0.31	3.05	3.26
Lcp1	1.46	0.16	0.06	3.04	0.14	0.09	0.28	3.26	3.22
Srgn	3.02	0.24	0.09	3.2	0.21	0.36	0.12	2.25	2.88
Arhgdib	1.08	0.37	0.02	3.42	0.16	1.74	0.37	2.74	2.75
Lyz2	0.96	0.86	0.39	3.14	0.89	0.89	0.76	4.41	4.33
Sfpi1	0.08	0.07	0.03	2.33	0.08	0.03	0.03	2.65	2.69
Lilrb4	0.09	0.08	0	1.74	0.05	0.07	0.03	2.41	2.82
Myo1f	0.05	0.1	0.05	2.34	0.04	0	0.05	2.19	2.17
Selplg	0.04	0.06	0.01	2.82	0.03	0.01	0.02	1.95	2.21
Cd52	0.07	0.07	0.02	2.75	0.03	0.04	0.04	2.13	2.48
Ptprc	0.13	0.2	0.04	3.12	0.1	0.09	0.08	2.97	3.26
Coro1a	0.12	0.11	0.05	3.38	0.07	0.18	0.11	1.99	2.66
Rac2	0.16	0.15	0.03	3.26	0.09	0.23	0.08	2.72	2.61
Itgb2	0.2	0.21	0.03	2.69	0.19	0.23	0.23	2.3	2.29
Laptm5	0.3	0.23	0.08	3.45	0.19	0.18	0.17	3.56	3.66
Cd53	0.16	0.17	0.02	3.22	0.1	0.12	0.1	3.31	3.36
Tyrobp	0.29	0.29	0.06	3.02	0.21	0.25	0.21	3.66	3.65
Fcer1g	0.87	0.44	0.09	2.83	0.33	0.35	0.27	3.65	3.62
Cybb	0.21	0.21	0.05	2.38	0.15	0.21	0.1	3.13	3.51
Lilrb3	0.02	0.07	0.02	2.03	0.03	0.05	0.06	1.87	1.66
Nfam1	0.16	0.07	0.01	2.23	0.05	0.06	0.03	1.97	2.06
Syk	0.24	0.18	0.02	2.12	0.06	0.08	0.05	2.06	2.11

Evi2b	0.15	0.12	0.11	2.43	0.05	0.14	0.06	1.29	1.79
Hmha1	0.09	0.19	0.32	2.68	0.09	0.37	0.09	2.11	2.22
Itgam	0.03	0.07	0.03	1.83	0.03	0.07	0.04	2.06	1.74
Ncf1	0.12	0.08	0.01	1.81	0.05	0.05	0.04	1.92	2.16
Slfn2	1.05	0.05	0.02	2.07	0.04	0.04	0.02	2.04	2.07
Hcls1	0.67	0.12	0.02	2.44	0.06	0.03	0.05	2.61	2.57
Nckap1l	0.1	0.17	0.03	2.32	0.09	0.18	0.06	2.81	2.74
Gpsm3	0.23	0.09	0.59	1.81	0.17	1.01	0.06	1.7	1.68
Ncf2	0.12	0.15	0.04	2	0.09	1.2	0.09	2.04	2.13
Alox5ap	0.09	0.16	0.02	2.76	0.08	0.05	0.23	2.07	1.78
Cfp	0.18	0.23	0.05	1.54	0.29	0.24	0.18	2.61	1.01
Prkcb	0.14	0.18	0.03	1.49	0.07	0.6	0.04	1.93	2.06
Ptpn6	0.1	0.17	0.2	2.79	0.07	0.22	0.08	2.69	2.91
Plbd1	0.14	0.18	0.02	2.28	0.15	0.1	0.14	1.84	2.38
Cytip	0.03	0.12	0.02	2.61	0.01	0.16	0.02	0.29	0.86
Msrb1	0.24	0.56	0.34	1.17	0.41	0.64	0.3	0.61	0.72
Mgst1	0.83	0.07	0.08	0.99	0.42	0.12	0.09	1.2	0.57
F630028O	0.02	0.03	0	1.02	0.01	0.02	0.02	1.28	0.81
Hk3	0.02	0.1	0.01	0.64	0.02	0.08	0.02	1.61	1.13
Csf3r	0.06	0.05	0	1.02	0.04	0.05	0.01	1.06	1.23
Gp49a	0.06	0.05	0.02	1.05	0.02	0.01	0.02	1.24	1.87
Ncf4	0.05	0.08	0.01	1.71	0.02	0.03	0	1.56	2.15
Dok3	0.31	0.12	0.05	1.34	0.02	0.05	0.05	1.67	1.87
Igsf6	0.11	0.13	0.02	1.61	0.05	0.09	0.05	2.49	2.46
Lrrc17	0.04	0.97	0.03	0.08	0.83	0.35	0.55	0.05	0.08
Nts	0.06	0.65	0.04	0.03	0.25	0.51	1.14	0.03	0.06
Ptgfr	0.06	0.19	0.04	0.03	0.16	0.27	0.78	0.06	0.06
Angptl1	0.17	0.77	0.02	0.06	0.85	0.13	1.47	0.11	0.12
Bmp3	0.04	0.1	0.03	0	0.03	0.03	0.02	0.02	0.06
Lrrc15	0.03	0.25	0.03	0.1	0.16	0.21	0.1	0.05	0.08
Dkk2	0.95	0.14	0.03	0.04	0.11	0.29	0.06	0.05	0.03
Lef1	1.35	0.22	0.82	0.14	0.09	0.42	1.39	0.04	0.07
Stmn2	0.37	0.05	0	0.01	0.04	0.03	0.03	0.05	0.03
Tnn	0.16	0.78	0.06	0.12	0.19	0.09	0.11	0.15	0.16
Ibsp	0.53	0.26	0.05	0.32	0.21	0.21	0.1	0.32	0.46
Jag1	1.08	0.46	1.52	0.12	0.84	2.91	0.9	0.11	0.17
Epas1	3.31	1.25	0.23	0.26	2.43	3.3	0.3	0.34	0.29
Sept4	2.29	1.52	1.16	0.06	1.69	3	1.01	0.19	0.15
Gucy1a3	0.18	0.85	0.05	0.08	1.75	2.89	0.53	0.16	0.09
Atp1b2	0.16	0.48	1.23	0.08	1.39	3.03	0.97	0.05	0.05
Ccdc80	0.14	0.66	0.06	0.11	0.89	2.49	0.77	0.04	0.05
Mylk	1.27	1.2	0.15	0.07	1.57	2.97	1.86	0.58	0.18
Aspn	0.07	0.07	0.01	0.05	0.05	3.23	0.04	0.06	0.06
Rgs5	0.36	0.26	0.12	0.21	0.21	4.09	0.16	0.3	0.46
Rasl12	0.03	0.07	0.08	0.05	0.1	2.41	0.06	0.02	0.02
Ndufa4l2	0.05	0.02	0.09	0.01	0	2.44	0.01	0.04	0.02
Aoc3	0.06	0.02	0	0.01	0	2.16	0	0.01	0.01
Steap4	0.14	0.04	0.03	0.02	0.02	2.55	0.03	0.13	0.05
Gucy1b3	0.2	0.36	0.04	0.05	1.07	2.4	0.05	0.08	0.04
Mgp	1.3	0.14	0.03	0.18	0.12	3.33	0.35	0.14	0.19
Heyl	0.07	0.11	1.23	0.03	0.22	2.82	0.11	0.07	0.05
Notch3	0.06	0.41	0.06	0.06	0.97	2.93	0.22	0.06	0.04

Col5a3	0.12	0.18	1.59	0.04	0.3	2.57	0.04	0.05	0.06
Calcrl	2.54	0.39	0.03	0.44	0.62	0.72	0.19	0.66	0.5
Prkch	2.51	0.14	0	0.81	0.05	0.1	0.05	1.03	1.01
Pde2a	2.66	0.08	0.06	0.62	0.05	0.21	0.03	0.8	0.63
Rgcc	2.46	0.39	0.28	0.29	0.19	0.09	0.16	0.05	0.1
Cav2	2.85	0.11	0.57	0.35	0.08	1.24	0.08	0.61	0.48
Flt1	3.39	0.36	0.06	0.31	0.31	0.45	0.36	0.35	0.32
Esam	3.57	0.11	0.04	0.12	0.1	2.54	0.1	0.06	0.1
Cav1	3.41	0.1	0.18	0.06	0.06	1.55	0.08	0.06	0.08
Rasip1	2.59	0.19	0.06	0.06	0.13	0.08	0.15	0.05	0.05
Kank3	2.58	0.17	0.3	0.26	0.13	0.06	0.12	0.38	0.45
Rasgrp3	2.27	0.1	0.05	0.25	0.04	0.1	0.03	0.39	0.52
Arhgef15	2.43	0.36	0	0	0.3	0.19	0.12	0.03	0.02
Icam2	2.5	0.06	0.01	0.82	0.02	0.22	0.04	0.14	0.14
Tmem88	2.81	0.14	0.06	0.09	0.06	0.08	0.1	0.21	0.52
Ly6a	3.02	0.06	0.03	0.58	0.03	0.17	0.11	0.12	0.13
Slco2a1	2.56	0.1	0.04	0.04	0.07	0.09	0.91	0.06	0.02
Clec14a	2.96	0.24	1.09	0.04	0.24	0.07	0.35	0.04	0.04
Egfl7	3.56	0.25	0.03	0.17	0.12	0.19	0.14	0.38	0.13
Gimap6	3	0.12	0.02	0.51	0.06	0.6	0.08	0.41	0.25
Sox18	3.13	0.19	0.02	0.01	0.03	0.25	0.51	0.03	0.02
Plvap	3.59	0.1	0.12	0.1	0.06	0.33	0.08	0.1	0.13
Tie1	3.15	0.05	0.03	0.06	0.03	0.54	0.03	0.06	0.09
Kdr	3.17	0.28	0.02	0.05	0.14	0.13	0.07	0.15	0.12
Emcn	3.8	0.12	0	0.19	0.08	0.61	0.09	0.09	0.1
Ly6c1	3.25	0.1	0.02	0.28	0.06	0.09	0.12	0.07	0.08
Cd34	3.67	0.15	0.04	0.6	0.07	0.09	0.07	0.09	0.16
Pecam1	3.47	0.13	0.03	0.55	0.05	0.07	0.07	0.18	0.25
Tek	2.7	0.15	0.01	0.03	0.21	0.98	0.04	0.04	0.02
Gpr116	3.17	0.52	0.04	0.08	0.63	0.89	0.09	0.07	0.07
Gimap4	2.47	0.02	0.01	0.22	0.01	0.01	0.02	0.03	0.03
Mmrn2	2.86	0.08	0.04	0.09	0.07	0.23	0.09	0.1	0.07
Robo4	2.95	0.02	0.02	0.04	0.01	0.02	0.02	0.03	0.05
Eltd1	3.12	0.07	0.02	0.22	0.03	0.04	0.04	0.04	0.05
Cdh5	2.99	0.11	0.03	0.13	0.02	0.03	0.02	0.1	0.08
Ecscr	2.73	0.05	0.14	0.02	0.02	0.14	0.01	0	0.03
Myct1	2.47	0.05	0	0.07	0	0.01	0.01	0.01	0.02
Ushbp1	2.75	0.08	0.06	0.02	0.02	0.03	0.03	0.08	0.02
Podxl	3.2	0.1	0.05	0.04	0.05	0.08	0.07	0.05	0.07
Ctla2a	2.92	0.04	0	0.44	0.02	0.06	0.02	0.1	0.08
Ptprb	3.22	0.22	0.06	0.05	0.03	0.12	0.04	0.08	0.08
Krt5	0.37	0.26	0.14	0.24	0.17	0.26	0.29	0.27	0.25
Krt14	0.26	0.17	0.08	0.18	0.13	0.1	0.14	0.12	0.17
Slc39a8	0.77	0.42	0.08	0.18	0.39	0.1	0.51	0.37	0.27
Perp	0.14	1.11	0.12	0.25	2.67	0.31	0.18	0.45	0.23
Spint2	0.26	0.17	0.02	0.84	0.2	0.34	0.23	0.03	0.05
Cldn1	0.08	0.09	0.04	0.22	0.07	0.24	0.13	0.05	0.04
Fxyd3	0.02	0.09	0.44	0.01	0.02	0.01	0.02	0	0.01
Col17a1	0.07	0.14	0.03	0.09	0.06	0	0.03	0.03	0.03
Trp63	0.09	0.17	0.05	0.05	0.07	0.03	0.03	0.03	0.09
Isl1	0.06	0.05	0.01	0.04	0.02	0.01	0.01	0.04	0.04
Cdh1	0.04	0.1	0.06	0.17	0.01	0.01	0.01	0.02	0.03

Pitx2	0.06	0.06	0.04	0.07	0.01	0.07	0.02	0.01	0.04
Dsc3	0.06	0.16	0.05	0.01	0	0.04	0.02	0.03	0.02
Dsp	0.14	0.23	0.06	0.06	0.12	0.09	0.22	0.04	0.07
Sorl1	0.08	0.74	0.12	1.42	0.93	0.1	0.26	0.41	0.75
Lgals3	0.14	0.1	1.17	2.41	0.02	0.21	0.03	0.74	0.9
Gsr	1.06	1.4	0.56	1.68	1.75	0.78	0.82	0.9	0.82
Mxd1	0.32	0.56	0.23	0.59	0.53	0.57	0.38	0.6	0.75
Pygl	0.1	0.7	0.12	1.11	1.43	0.42	0.6	0.42	0.26
Dgat1	0.64	0.81	0.4	0.74	0.91	0.67	0.45	0.49	0.45
S100a6	1.45	0.59	3	2.15	0.58	2.82	1.1	0.12	0.28
Cd44	0.16	0.62	1.36	2.59	0.88	0.63	0.19	0.72	0.83
Ctsc	0.53	0.33	0.64	2.55	0.29	2.33	0.37	3.77	3.79
Fam105a	0.13	0.23	0.05	2.32	0.19	0.4	0.3	2.77	3.13
Ctsh	0.75	0.42	0.19	2.38	0.86	0.8	0.23	3.37	3.43
Unc93b1	1.18	0.99	0.77	2.59	0.95	1.02	0.61	3.42	3.44
Ctss	0.64	0.55	0.09	2.93	0.51	0.57	0.39	3.89	4.25
Csf1r	0.68	0.66	0.12	2.05	0.61	0.74	0.76	4.07	3.99
H2-Eb1	0.88	0.57	0.08	2.09	0.43	0.49	0.49	2.31	3.84
H2-Ab1	1.32	0.73	0.1	2.29	0.67	0.64	0.59	2.47	4.15
H2-Aa	1.07	0.74	0.09	2.19	0.65	0.67	0.6	2.57	4.3
Cd74	1.3	1.06	0.2	2.88	0.93	0.92	0.86	3.26	4.65
Trf	1.31	2.08	0.57	2.02	3.68	1.38	1.71	4.02	3.78
Lgmn	1.54	2.13	1.57	1.49	2.96	1.91	0.69	3.73	3.81
Apoe	2.21	1.83	3.32	1.79	2.37	1.38	1.81	4.86	4.92

cluster: Pre	cluster: SI_	cluster: E-p	cluster: VS	cluster: OE	cluster: Os	cluster: An	cluster: Inr	gene module	
0.05	0.04	0.04	0.04	0.05	0.09	0.24	1.56	1	
0.27	0.06	0.11	0.12	0.06	0.34	0.18	1.14	1	
0.14	0.07	0.24	0.17	0.17	0.31	0.07	0.54	1	
0.12	0.07	0.17	0.56	0.12	0.06	0.05	2.25	1	
0.15	0.02	0.17	0.12	0.04	0.12	0.02	2.03	1	
0.16	0.06	0.08	0.01	0.11	0.18	0.24	1.75	1	
0.12	0.05	0.02	0.06	0.03	0.1	0.04	2.54	1	
0.13	0.39	0.24	0.11	0.05	0.17	0.04	1.98	1	
0.06	0.03	0.02	0.06	0.04	0.07	0.02	2.34	1	
0.03	0.02	0.1	0.04	0.04	0.15	0	2.33	1	
0.11	0.08	0.1	0.09	0.17	0.18	0.05	2.73	1	
0.04	0.03	0.05	0.03	0.08	0.1	0.04	3.1	1	
0.06	0.01	0.09	0.06	0.05	0.14	0.03	1.17	1	
0.03	0.04	0.09	0.09	0.04	0.05	0.03	1.48	1	
0.14	0.04	0.11	0.11	0.04	0.2	0.09	1.59	1	
0.05	0.02	0.07	0.08	0.05	0.06	0.11	1.28	1	
0.02	0.02	0.05	0.01	0.07	0.09	0.08	1.71	1	
0.16	0.04	0.14	0.12	0.06	0.15	0.16	1.32	1	
0.15	0.21	0.14	0.12	0.48	0.22	0.09	0.45	1	
0.07	0.06	0.05	0.03	0.03	0.08	0.06	0.78	1	
0.09	0.01	0.15	0.11	0.08	0.14	0.08	0.68	1	
0.13	0.2	0.36	0.48	0.82	1.12	0.33	0.12	1	
0.22	0.14	0.08	0.08	0.24	0.27	0.08	0.29	1	
0.11	0.1	0.12	0.06	0.33	0.09	0.35	0.32	1	
0.09	0.02	0.1	0.05	0.09	0.1	0.04	1.08	1	
0.02	0.03	0.08	0.05	0.05	0.1	0.02	0.27	1	
0.06	0.01	0.04	0.1	0.06	0.09	0	0.04	1	
0.07	0.05	0.05	0.02	0.02	0.08	0.04	0.15	1	
0.1	0.04	0.08	0.11	0.12	0.2	0.09	0.08	1	
0.08	0.05	0.1	0.08	0.03	0.1	0.05	0.13	1	
0.22	0.13	0.26	0.18	0.15	0.28	0.28	0.14	1	
0.04	0.02	0.04	0.04	0.05	0.06	0.03	0.07	1	
0.04	0.03	0.08	0.02	0.12	0.05	0	0.04	1	
0.03	0.03	0.03	0.06	0.1	0.09	0.07	0.06	1	
0.05	0.03	0.05	0.09	0.04	0.05	0.07	0.06	1	
0.06	0.01	0.07	0.04	0.02	0.11	0.04	0.09	1	
0.04	0.02	0.09	0.06	0.02	0.05	0.02	0.05	1	
0.02	0.05	0.08	0.01	0.02	0.09	0.04	0.15	1	
0.17	0.05	0.16	0.02	0.09	0.05	0.75	2.65	2	
0.03	0.04	0.07	0.08	0.08	0.03	0.02	2.42	2	
0.09	0.16	0.08	0.12	0.15	0.04	0.08	2.85	2	
0.08	0.22	0.33	0.2	0.21	0.21	0	3.84	2	
0.02	0	0.03	0.05	0	0	0	2.55	2	
0	0.01	0.03	0.01	0.01	0	0.17	2.3	2	
0.05	0.01	0	0.01	0.02	0.01	0	2.78	2	
0	0.08	0.05	0.05	0.02	0	0	2.33	2	
0.03	0.01	0.05	0.05	0.04	0.03	0	2.37	2	
0	0.01	0.02	0.02	0.01	0.01	0	2.24	2	
0.27	0.23	0.48	0.57	0.21	0.38	0	2.95	2	
0.05	0.06	1.53	1.77	0.14	0.53	0.04	3.47	2	
0.03	0.06	0.04	0.12	0.03	0.04	0	3.42	2	

0	0.01	0	0.03	0	0.05	0	2.7	2	
0.02	0.03	0.02	0.04	0.05	0.03	0.09	2.79	2	
0.02	0.02	0.05	0.03	0.04	0.01	0.02	2.93	2	
0.17	0.33	0.07	0.06	0.06	0.11	0	2.38	2	
0.09	0.04	0.04	0.04	0.05	0.02	0.3	3.18	2	
0	0	0.05	0.05	0.02	0.02	0	2.9	2	
0.01	0.08	0.07	0.19	0.03	0.03	0	3.71	2	
0.02	0	0.03	0.1	0.08	0	0	2.55	2	
0.04	0.05	0.12	0.09	0.06	0.08	0	2.85	2	
0.1	0.1	0.04	0.03	0.31	0.11	0.62	3.02	2	
0.01	0.02	0	0	0.02	0	0	2.33	2	
0.04	0.07	0.08	0.17	0.05	0.04	0	2.65	2	
0.02	0.02	0.03	0.02	0	0.01	0	2.62	2	
0.01	0.01	0.02	0.03	0.03	0.01	0	2.46	2	
0	0	0.02	0.01	0.02	0	0	2.42	2	
0.01	0.01	0.01	0.01	0.02	0.04	0	2.32	2	
0.02	0.02	0.13	0.01	0	0.06	0.04	2.34	2	
0.02	0.08	0.07	0.11	0.06	0.02	0	2.85	2	
0.05	0.03	0.03	0.14	0.05	0.12	0.02	2.85	2	
0.06	0.06	0.07	0.11	0.04	0.05	0	3.23	2	
0.01	0.06	0.08	0.01	0.04	0.02	0.04	3.48	2	
0.01	0.04	0.05	0.08	0.06	0.01	0	3.48	2	
0.02	0.02	0.05	0.02	0.05	0.02	0.03	2.67	2	
0.03	0.05	0.05	0.03	0.05	0	0.03	3.16	2	
0.18	0.26	0.51	0.55	0.44	0.29	0.08	3.74	2	
0.09	0.19	0.32	0.33	0.25	0.16	0.07	4.07	2	
0.1	0.11	0.32	0.25	0.22	0.27	0.08	2.72	2	
0.11	0.15	0.24	0.26	0.27	0.1	0	2.85	2	
0.08	0.13	0.24	0.17	0.18	0.09	0	2.55	2	
0	0.04	0.07	0.06	0.04	0.08	0.02	2.35	2	
0.19	0.38	0.64	0.67	0.59	0.5	0.28	5.1	2	
0.27	0.42	0.74	0.77	0.77	0.58	0.08	5.33	2	
3.27	0.42	3.46	3.33	0.25	3.68	0.32	0.23	3	
3.28	0.08	3.44	3.37	0.22	3.84	0.45	0.48	3	
3.07	0.51	3.7	3.22	0.46	4.12	0.88	0.4	3	
2.06	0.23	2.84	2.84	0.6	2.96	0.2	0.12	3	
2.22	0.64	2.81	3.56	0.12	2.35	0.39	0.17	3	
2.31	0.93	2.45	3.31	1.07	2.6	0.32	0.14	3	
2.5	2.49	2.7	3.76	1.29	1.99	0.12	0.27	3	
3.7	1.82	2.36	1.89	0.69	3.53	0.15	0.13	3	
0.99	0.05	2.14	3.31	0.14	2.08	0.29	0.16	3	
1.77	0.13	2.29	2.61	0.15	2.65	0.08	0.2	3	
3.16	0.26	2.3	2.24	0.16	2.45	0.19	0.17	3	
2.31	0.16	2.63	2.38	0.61	3.72	0.17	0.14	3	
3.03	0.08	2.48	0.8	0.09	3.64	0.32	0.14	3	
4.18	0.16	3.23	1.33	0.59	4.31	0.96	0.47	3	
3.27	0.11	1.96	0.74	0.59	3.78	0.36	0.24	3	
3.31	0.57	4.01	3.65	1.28	4.29	2	0.64	3	
3.56	0.33	3.98	3.55	0.75	4.2	0.84	0.75	3	
2.8	0.34	4.31	3.92	0.76	4.4	1.02	1	3	
0.08	0.1	1.46	0.17	0.1	0.41	0.08	0.51	4	
0.3	0.34	0.21	0.08	0.22	0.09	0.31	0.83	4	

0.17	0.05	1.34	1.13	0.18	0.29	0.27	0.34	4	
0.28	0.06	1.7	0.74	0.56	0.34	0.19	1.21	4	
0.18	0.07	0.45	2.96	0.19	0.23	0.1	0.25	4	
0.7	0.28	0.71	1.03	0.64	0.6	0.5	0.24	4	
0.97	1.3	0.98	0.77	0.76	1.09	1.13	0.74	4	
0.77	0.16	1.95	1.79	0.25	1.16	0.6	0.13	4	
0.29	0.13	1.29	0.98	0.08	0.9	2.07	1.89	4	
0.07	0.05	0.14	0.09	0.04	0.07	0.07	0.11	4	
0.12	0.05	0.07	0.15	0.07	0.06	0.05	0.16	4	
0.18	0.09	0.27	0.39	0.28	0.16	0.26	0.07	4	
0.1	0.64	0.09	0.04	1.73	0.13	1.61	0.05	4	
0.05	0.22	0.04	0.08	0.66	0.11	2.81	0.58	4	
0	0	0.02	0	0.02	0.04	0	0.12	4	
0.23	0.06	0.11	0.03	0.09	0.19	0.28	0.13	4	
0.4	0.08	0.23	0.28	0.06	0.17	0.13	0.02	4	
0.18	0.02	0.08	0.11	0.13	0.27	0.03	0.14	4	
0.15	0.02	0.12	0.06	0.03	0.2	0.16	0.15	4	
0.09	0.02	0.05	0.07	0	0.13	0.07	0.31	4	
0.43	0.11	0.47	0.56	0.05	0.46	0.46	1.1	4	
0.67	0.47	1.08	1.05	0.39	0.76	0.33	0.29	4	
0.42	0.18	0.52	1.9	0.16	0.43	0.22	0.52	4	
0.09	0.04	0.05	0.13	0.05	0.14	0.03	0.28	4	
0.09	0.07	0.15	0.14	0.02	0.12	0.2	1.04	4	
0.06	0.04	0.03	0.07	0	0.13	0.13	0.05	4	
0.16	0.16	0.13	0.13	0.13	0.54	0.45	0.08	4	
0.32	0.41	0.68	0.28	0.49	1.14	0.4	0.16	5	
0.78	0.3	0.33	0.2	0.21	0.2	0.02	1.25	5	
0.14	0.4	0.34	0.31	1.08	0.14	0.41	0.13	5	
0.31	0.13	2.54	1.25	0.87	0.34	0.03	0.06	5	
0.28	1.77	0.72	0.32	1.23	0.07	0.98	0.03	5	
0.4	1.29	0.44	0.55	0.61	0.22	0.06	0.34	5	
0.66	1.39	1.33	0.28	1.38	0.19	0.02	0.01	5	
0.27	0.04	0.21	0.33	0.26	0.27	0.15	0	5	
0.69	0.48	0.22	0.93	0.14	0.13	0	0.07	5	
0.04	0.09	0.1	0.76	0.04	0.03	0	0.01	5	
0.01	0.02	0.01	0	0.03	0.06	0	0.01	5	
0.05	0.13	0.02	0.06	0.03	0.1	0	0.01	5	
0.1	0.07	0.02	0.01	0.09	0	0	0.01	5	
0.02	0.03	0.01	0.16	0.03	0.01	0	0.03	5	
0.07	0.22	0.04	0.04	0.24	0.09	0.14	0	5	
0.08	0.03	0.03	0.05	0.06	0.02	0	0.01	5	
0.08	0.04	0.06	0.02	0.04	0.05	0	0	5	
0.02	0.04	0.03	0.05	0.09	0.01	0	0.01	5	
0.01	0.04	0.07	0.03	0.01	0	0	0.03	5	
0.06	0.01	0.04	0.12	0.04	0.05	0	0.05	5	
0.07	0.1	0.06	0.34	0.12	0.02	0	0.05	5	
0.08	0.15	0.08	0.16	0.11	0.11	0	0.05	5	
0.04	0.32	0	0.02	0.15	0.02	0.56	0.06	5	
0.02	0.05	0.03	0.02	0.01	0	0.14	0.01	5	
0.13	0.04	0.25	0.09	0.06	0.15	0	0	5	
0.03	0.21	0.07	0	0.08	0.01	0.69	0.31	5	
0.86	0.07	0.1	0.07	0.08	0.32	0	0.09	5	

0.38	0.29	0.31	0.31	0.22	0.09	0.04	0.04	5	
0.01	0.09	0.05	0.13	0.2	0.06	0.06	0.02	5	
0.04	0.1	0.13	0.52	0.37	0.02	0	0.02	5	
0.09	0.14	0.53	0.31	0.08	0.53	0	0.03	5	
0.03	0.31	0.06	0.23	0.41	0.01	2.12	0.02	5	
2.77	0.1	0.14	0.07	0.09	0.29	0.05	0.03	6	
2.82	0.51	0.14	0.16	1.6	0.1	0.41	0.18	6	
2.43	2.98	0.12	0.15	0.82	1.41	1.6	0.12	6	
1.06	2.02	2.95	0.45	0.57	1.28	0.32	0.12	6	
0.28	1.82	0.25	0.53	1.79	0.24	0.19	0.06	6	
0.03	0.46	0.02	1.77	2.6	0.04	0	0.02	6	
0.19	2.87	0.04	0	1.57	0.02	2.14	0.03	6	
0.02	2.76	0.02	0.07	1.29	0.01	1.17	0.09	6	
0.1	2.55	0.19	0.19	0.54	0.02	0	0.4	6	
0.13	2.47	0.07	0.01	1.07	0.01	0.05	0.07	6	
1.11	2.49	0.22	0.24	1.52	0.2	0.25	0.04	6	
0.01	0.02	0.02	0.02	0.07	0.02	0.07	0.11	6	
0.05	0.16	0.06	0.05	0.05	0.08	0.28	0.13	6	
0.01	0.11	0.04	0.02	0.14	0.01	0	0.98	6	
0.09	0.72	0.13	0.2	0.2	0.06	0	1.46	6	
0.85	0.25	3.25	2.3	0.53	4.08	1.16	0.44	7	
0.16	0.1	1.07	2.79	0.2	3.16	0.46	1.07	7	
1.11	0.33	3.44	3.19	0.27	2.55	0.21	0.17	7	
0.84	0.45	3.23	3.52	0.58	2.37	0.04	0.42	7	
1.37	0.17	3.04	3.05	0.14	2.57	0.18	3.39	7	
0.14	0.02	2.21	3.15	0.12	1.53	0.09	1.75	7	
0.31	0.05	3	3.08	0.16	1.11	0.16	0.13	7	
0.21	0.01	0.46	2.66	0.05	0.27	0.09	0.09	7	
0.16	0.2	0.43	2.57	0.06	1.21	0.02	0.02	7	
0.13	0.29	1.09	2.8	2.46	0.32	0.48	0.26	7	
0.12	0.02	3.18	2.99	0.21	0.45	0.09	0.14	7	
0.07	0.03	1.15	2.31	0.04	0.02	0	0.02	7	
0.37	0.02	2.47	1.78	0.04	1.09	0.03	0.07	7	
0.16	0.23	1.35	2.54	0.38	1.24	0.73	0.04	7	
1.59	0.15	2.56	1.29	0.08	0.35	0	0.03	7	
1.3	0.02	2.86	2.73	0.04	2.11	0.13	0.1	7	
0.07	0.14	2.06	2.57	0.32	0.5	0.02	0.22	7	
0.31	0.24	1.72	3.59	0.4	0.35	0.06	1.15	7	
2.02	0.19	1.76	3.53	0.29	0.9	1.37	1.15	7	
0.38	0.06	2.48	3.21	0.42	0.31	0.07	0.09	7	
1.57	0.19	1.92	4.19	0.3	0.46	0.92	0.83	7	
0.49	0.33	1.83	2.89	0.42	0.37	1.76	0.23	7	
2.45	0.09	2.94	2.93	0.09	0.56	0.2	0.05	7	
1.13	0.72	0.67	1.27	0.65	0.25	0.13	0.06	7	
0.64	0.1	0.86	2.33	0.11	0.28	0.22	0.05	7	
3.04	0.13	0.86	1.68	0.17	1.44	0.06	0.03	7	
0.57	0.81	3.08	3.91	2.9	0.74	1.02	0.4	7	
0.12	0.06	0.1	0.07	0.07	0.18	0.13	0.11	8	
0.12	0.03	0.11	0.14	0.15	0.22	0.18	0.35	8	
0.2	0.08	0.2	0.13	0.2	0.33	0.09	0.16	8	
0.16	0.04	0.14	0.24	0.11	0.13	0.07	0.05	8	
0.11	0.12	0.19	0.17	0.22	0.2	0.49	0.03	8	

0.19	0.1	0.23	0.2	0.23	0.29	0.16	0.25	8	
0.22	0.08	0.25	0.17	0.26	0.42	0.26	0.39	8	
0.27	0.07	0.15	0.19	0.13	0.24	0.17	0.25	8	
0.42	0.15	0.37	0.32	0.45	0.49	0.42	0.38	8	
0.03	0.01	0.01	0.04	0.04	0.07	0.05	0.08	8	
0.04	0.01	0.02	0.01	0.04	0.06	0.05	0.04	8	
0.08	0.04	0.03	0.02	0.04	0.03	0.05	0.18	8	
0.1	0.02	0.08	0.05	0.02	0.09	0.11	0.14	8	
0.05	0.01	0.01	0.08	0.09	0.13	0.27	0.08	8	
0.14	0.04	0.05	0.05	0.16	0.11	0	0.03	8	
0.09	0.05	0.02	0.07	0.11	0.09	0.14	0.14	8	
0.06	0	0.02	0.01	0.03	0.06	0.1	0.02	8	
0.12	0.02	0.05	0.06	0.04	0.08	0.1	0.02	8	
0.09	0.04	0.07	0.08	0.18	0.15	0.13	0.09	8	
0.06	0.01	0.04	0.04	0.02	0.1	0.04	0.65	8	
0.01	0.03	0.03	0.05	0.06	0.12	0.14	0.92	8	
0.09	0	0.02	0.04	0.04	0.07	0.02	0.26	8	
0.03	0.01	0.06	0.04	0.02	0.03	0	0.04	8	
0.01	0.03	0.08	0.02	0.11	0.04	0.02	0.4	8	
0.02	0.01	0.03	0.04	0	0.04	0.06	0.05	8	
0.07	0	0.03	0.01	0.04	0.04	0.03	0.12	8	
0.2	0.03	0.1	0.1	0.07	0.13	0.13	0.11	8	
0.23	0.06	0.09	0.13	0.05	0.06	0.04	0.03	8	
0.14	0.04	0.08	0.1	0.1	0.15	0.05	0.09	8	
0.07	0.01	0.05	0.04	0.01	0.04	0.04	0.06	8	
0.03	0.09	0.21	0.32	0.11	0.14	0.03	0.11	8	
0.04	0.18	0.07	0.11	0.08	0.05	0.08	0.05	8	
0.03	0	0.02	0	0.01	0.03	0	0.06	8	
0.01	0.69	0.01	0.03	0.64	0.02	0.23	0.16	8	
0.44	1.11	2.34	2.21	1.27	0.51	0.3	0.1	9	
0.42	1.38	2.27	2.1	1.42	0.38	0.17	0.21	9	
1.94	0.47	3.45	2.72	0.17	1.41	0.06	0.15	9	
0.5	0.31	1.1	2.34	0.4	1.44	0.17	0.16	9	
0.91	1.24	2.62	2.81	1.99	0.46	0.23	0.31	9	
0.15	1.02	0.52	0.39	1.01	0.13	0.25	0.17	9	
0.08	1.34	0.11	0.67	1.07	0.19	0.18	0.02	9	
0.06	0.08	0.38	0.25	0.15	0.13	0.13	0.06	9	
0.21	0.14	0.17	0.46	0.29	0.12	0	0	9	
0.8	0.41	0.99	1	0.44	0.48	0.11	0.03	9	
0.17	1.27	0.38	0.46	0.37	1.23	2.75	0.05	9	
0.44	0.16	0.21	0.78	0.25	3.07	0.53	0.19	9	
0.56	0.75	1.19	1.05	0.42	1.72	2.16	0.07	9	
0.47	0.08	1.38	1.49	0.24	1.45	0.13	0.1	9	
0.57	0.39	0.51	0.66	0.38	0.28	0.26	0.18	9	
0.81	0.12	1.66	1.17	0.09	1.57	0.31	0.12	9	
0.23	0.09	0.98	1.35	0.13	0.54	0.04	0.06	9	
0.94	0.44	1.74	1.19	0.71	1.07	0.02	0.05	9	
0.36	0.91	0.38	0.33	1.08	0.45	2.64	0.01	10	
0.14	2.06	1.31	0.29	0.36	0.26	3.08	0.03	10	
0.21	1.87	0.16	0.1	1.38	0.2	2.75	0.13	10	
0.02	1.48	0.05	0.13	0.86	0.01	2.57	0.01	10	
0.11	2.28	0.05	0.07	1.23	0.23	3.07	0.11	10	

0.38	1.3	0.03	0.04	0.65	0.34	2.96	1.02	10	
0.08	1.95	0.07	0.05	1.8	0.1	2.73	0.14	10	
0.14	1.71	0.01	0.01	1.52	0.05	2.91	0	10	
0.09	1.3	0.13	0.04	1.57	0.04	3.05	0	10	
0.15	1.86	0.02	0	1.35	0.06	3.16	0.01	10	
0.02	2.55	0.08	0	1.77	0.12	3.24	0.07	10	
0	0.75	0	0	0.38	0.01	2.55	0.01	10	
0	1.23	0.02	0.01	1.68	0	2.91	0.01	10	
0.02	1.13	0	0	1.07	0.06	2.57	0.01	10	
0	0.56	1.07	0.29	1.53	0.2	2.69	0	10	
0.5	0.63	0.86	0.08	0.15	0.33	2.39	0.03	10	
0.02	0.85	0.02	0.1	1.08	0.06	2.8	0.01	10	
0.02	0.33	0.07	0.03	0.8	0.22	2.72	0.03	10	
0.35	0.94	0.05	0.06	0.98	0.25	2.58	0	10	
0.04	1.87	0.08	0.03	2.02	0.11	3.24	0.08	10	
0.01	0.33	0.04	0.08	1.46	0.04	3.03	0	10	
0.06	1.65	0.28	0.39	1.93	0.09	3.42	0.06	10	
0	0.46	0	0.03	1.34	0.06	3.3	0.08	10	
0.05	0.54	0.07	0.05	0.49	0.07	2.85	0.09	10	
0.16	0.66	0.02	0.05	0.7	0.82	2.88	0.04	10	
0	0.47	0.03	0	0.8	0.01	2.55	0.04	10	
0.05	0.06	0.05	0.06	0.49	0.23	4.79	0.02	10	
0.03	0.46	0.06	0.02	0.43	0.17	4.19	0.01	10	
0.2	0.49	0.15	0.11	0.65	0.58	5.46	0.21	10	
0.1	0.09	0.09	0.34	0.22	0.1	2.54	0.13	10	
0.12	1.03	0.14	0.22	0.37	0.19	2.78	0.02	10	
0	0.27	0	0.06	0.33	0.02	2.54	0	10	
0	0.11	0	0.01	0.18	0.03	2.59	0	10	
0.04	0.18	0.09	0.06	0.13	0.1	2.27	0.03	10	
0	0.19	0	0	0.24	0.01	2.22	0	10	
0.01	0.21	0.01	0.01	0.32	0.04	2.8	0.02	10	
0.01	0.06	0	0	0	0.03	2.47	0	10	
0.02	0.03	0.05	0	0.08	0	2.3	0	10	
0.02	0.19	0	0.01	0.05	0.03	2.31	0.28	10	
0.01	0.21	0.02	0	0.25	0	2.47	0.01	10	
0.06	0.39	0.02	0.04	0.55	0.01	2.59	0.02	10	
0.06	0.57	0.02	0	0.04	0.01	2.99	0	10	
0.03	0.67	0.04	0.01	0.31	0.05	2.88	0.12	10	
2.65	1.34	2.71	2.73	1.9	3	3.64	0.11	11	
3.08	2.02	1.4	1.82	0.51	2.7	3.21	0.02	11	
2.11	2.5	1.87	1.56	2.62	1.63	3.83	1.11	11	
0.28	0.3	1.52	0.28	2.71	3.18	3.57	0.26	11	
1.93	0.78	0.18	0.2	0.42	0.24	3.07	0.11	11	
0.17	1.5	0.15	0.42	1.84	0.3	3.02	0.01	11	
1.24	2.03	0.29	1.01	1.72	0.84	4.83	0.26	11	
1.93	0.65	1.19	1.86	0.94	0.84	3.66	0.02	11	
1.06	1.78	1.46	1.21	1.54	0.67	3.18	0.16	11	
3.3	1.77	0.69	0.48	1.08	0.91	2.11	0.57	11	
0.91	1.18	0.9	0.58	1.16	0.9	3.07	0.26	11	
0.69	1.2	0.73	0.38	0.84	0.49	2.87	0.1	11	
0.2	0.17	1.75	0.83	0.28	1.6	2.85	0.04	11	
1.11	0.31	1.01	0.7	0.11	1.45	2.94	0.04	11	

0.49	0.75	0.62	0.68	1.03	0.41	3.14	0.01	11	
0.62	1.44	0.54	0.58	1.68	1.24	3.19	0.05	11	
0.61	1.82	0.24	0.3	1.53	1.19	3.22	0.18	11	
0.82	1.48	1.31	1.21	2.04	1.87	3.27	0.53	11	
0.23	1.98	0.07	0.02	2.12	0.08	3.16	0.19	11	
0.04	2.32	0.16	0.09	2.2	0.12	3.04	0.1	11	
0.55	1.54	0.47	0.53	2.41	0.88	3.63	0.42	11	
1.07	0.37	2.7	2.73	0.56	2.37	0.45	0.16	12	
0.6	0.14	3.31	2.98	0.93	1.43	0.21	0.23	12	
0.68	0.59	1.64	3.87	2.26	1.17	3.66	0.73	12	
1.34	2.21	0.72	0.84	2.3	0.73	2.77	0.27	12	
1.62	1.73	1.43	2.62	2.64	0.4	1.24	0.27	12	
1.15	0.29	1.41	1.09	2.65	1.57	0.86	0.12	12	
0.97	1.39	2.27	0.86	1.19	0.55	0.36	0.22	12	
1.01	0.98	0.65	0.97	0.37	1.07	0.05	0.06	12	
2.23	0.83	1.11	0.86	0.73	1.14	0.38	0.35	12	
0.85	0.09	0.29	1.07	0.71	0.48	0.05	0.08	12	
2.86	0.05	0.26	0.37	0.11	0.43	0.14	0.05	12	
1.4	0.36	0.86	0.75	0.39	0.38	0	0.03	12	
2.37	0.03	1.23	1.65	0.09	0.53	0.15	0.1	12	
2.66	0.1	2.01	1.06	0.09	0.56	0.2	0.13	12	
2.1	0.12	0.35	0.3	0.13	0.48	0.11	1.21	12	
1.1	0.5	0.32	0.92	0.5	0.77	0.16	0.02	12	
0.76	0.1	0.34	0.81	0.43	0.55	0.35	0.24	12	
0.22	0.16	0.04	0.02	0.19	0.23	0	0.01	12	
0.31	0.07	0.1	0.07	0.01	0.28	0.25	0.02	12	
1.61	0.87	0.05	0.05	0.14	0.33	0.06	0.01	12	
0.18	0.35	0.14	0.59	0.84	0.27	0.21	0.02	12	
1.08	0.12	0.07	0.12	0.06	0.35	0.17	0.06	12	
0.19	0.19	0.13	0.49	0.33	0.11	2.86	2.72	13	
0.09	0.37	0.56	1.28	0.31	0.6	1.17	3.14	13	
0.13	0.1	0.23	1.1	0.22	0.15	0.29	3.75	13	
0.18	0.08	0.24	0.74	0.27	0.22	0.28	2.89	13	
0.51	0.66	0.44	0.95	0.41	0.19	0.15	3.82	13	
0.74	0.48	0.97	0.99	0.88	1.02	0.91	4.42	13	
0.03	0.01	0.07	0.08	0.09	0.06	0	2.82	13	
0.04	0.01	0.08	0.06	0	0.06	0.11	2.93	13	
0.03	0.06	0.09	0.04	0.07	0.06	0.03	3.05	13	
0.03	0.03	0.05	0.02	0.02	0.09	0.12	2.85	13	
0.03	0.02	0.08	0.03	0.09	0.04	0	3.42	13	
0.12	0.07	0.08	0.17	0.13	0.18	0.06	3.37	13	
0.2	0.15	0.16	0.2	0.15	0.21	0.16	3.6	13	
0.05	0.06	0.16	0.11	0.16	0.14	0.06	3.72	13	
0.13	0.17	0.22	0.23	0.16	0.18	0.44	3.24	13	
0.26	0.08	0.09	0.18	0.17	0.31	0.12	3.19	13	
0.12	0.06	0.11	0.11	0.07	0.2	0.16	3.16	13	
0.19	0.15	0.22	0.14	0.26	0.25	0.21	3.72	13	
0.31	0.12	0.33	0.25	0.38	0.33	0.44	3.53	13	
0.13	0.08	0.14	0.1	0.18	0.17	0.23	2.58	13	
0.02	0.03	0.05	0.03	0.06	0.05	0.04	2.85	14	
0.08	0.01	0.02	0.07	0.06	0.07	0	3.04	14	
0.08	0.17	0.08	0.07	0.13	0.11	0.04	2.66	14	

0.18	0.15	0.09	0.2	0.03	0.07	0.05	3.02	14	
0.3	0.1	0.06	0.06	0.02	0.09	0.35	2.84	14	
0.03	0.04	0.04	0.11	0.03	0.05	0.11	3.18	14	
0.11	0.02	0.1	0.09	0.07	0.06	0	3.35	14	
0.03	0.04	0.12	0.03	0.07	0.09	0.03	3.19	14	
0.08	0.01	0.13	0.03	0.07	0.1	0.04	2.7	14	
0.06	0.06	0.13	0.07	0.11	0.09	0	2.04	14	
0.07	0.1	0.22	0.39	0.07	0.18	0.03	2.69	14	
0.11	0.09	0.08	0.11	0.08	0.07	0.08	2.87	14	
0.16	0.11	0.95	0.44	0.13	0.72	0.2	3.61	14	
0.22	0.28	0.21	0.18	0.31	0.27	0.07	2.17	14	
0.09	0.58	0.1	0.88	0.19	0.08	0.03	2.67	14	
0.11	0.88	0.1	0.05	0.69	0.11	0.7	2.78	14	
0.31	0.41	0.37	0.22	0.26	0.27	1.6	2.99	14	
0.06	0.02	0.08	0.01	0.1	0.06	0.08	2.09	14	
0.5	0.53	0.41	0.37	0.6	0.39	0.52	2.75	14	
0.01	0.13	0.12	0.37	0.38	0.08	0.1	2.54	14	
0.02	0.04	0.02	0.03	0.02	0.02	0.02	2.73	14	
0.01	0.02	0.02	0.02	0.01	0.01	0	2.57	14	
0.02	0.05	0.04	0.03	0.02	0.04	0.04	3.16	14	
0.01	0.01	0.03	0.04	0.01	0.05	0	2.89	14	
0.04	0.03	0.01	0.02	0.01	0.06	0	2.58	14	
0.09	0.09	0.1	0.09	0.16	0.13	0.07	2.57	14	
0.07	0.06	0.09	0.06	0.15	0.11	0.11	2.62	14	
0.56	0.02	2.52	0.38	0.09	2.95	0.02	0.05	15	
1.4	0	2.5	1.89	0.05	2.71	0.1	0.11	15	
0.08	0.03	1.87	2.19	0.11	2.55	0.09	0.01	15	
0.39	0.38	1.26	0.46	0.09	2.67	0.14	0.06	15	
0.01	0.05	2.13	0.12	0.07	2.54	0.03	0.06	15	
1.76	0.01	1.99	0.24	0.1	2.77	0.16	0.07	15	
0.22	0.02	2.38	0.43	0.03	0.77	0	0.04	15	
0.91	0.06	2.63	0.49	0.06	1.62	0.05	0.04	15	
0.04	0.13	0.11	0.01	0.04	2.51	1.62	0.01	15	
0.35	0.12	1.55	0.45	0.2	3.33	0.46	0.12	15	
0.29	0.19	1.46	0.26	0.65	3.54	1.08	0.44	15	
0.42	1.92	1.05	0.92	1.09	0.36	0.86	0.12	16	
0.24	0.59	0.13	0.29	1.39	0.39	0.6	0.13	16	
0.91	0.14	1.07	1.36	0.26	0.92	1.02	0.07	16	
0.17	0.21	0.93	1.24	0.11	0.13	0.07	0.02	16	
0.2	0.1	0.37	1.13	0.1	0.13	0.04	0.01	16	
0.19	0.03	0.41	2.59	0.12	0.4	0.02	0.21	16	
0.15	0.07	1.07	2.27	0.14	0.39	0.06	0.09	16	
0.06	0.04	0.12	0.16	0.1	1	0.1	0.03	16	
0.19	0.07	0.16	0.18	0.08	0.17	0	0.12	16	
0.13	0	0.02	0.01	0	0.04	0	0.01	16	
0	0.03	0.01	0	0.08	0.22	0.16	0	16	
0	0	0	0.3	0.04	0.02	0	0	16	
0	0.01	0.05	0.88	0.07	0.04	0	0.08	16	
0.55	0.35	0.52	0.41	0.08	0.18	0	0	16	
0.07	0.24	0.14	0.85	0.28	0.13	0.1	0.1	16	
0.09	0.67	0.14	0.64	0.16	0.06	0	0.03	16	
0.27	0.66	0.11	0.76	0.22	0.1	0.09	0.03	16	

0.11	0.1	0.09	0.08	0.03	1.38	0.19	0.04	16	
0.11	0.03	0.03	0.04	0.28	0.11	0	0.52	17	
0.12	0.52	0.19	0.19	0.66	0.15	0.64	0.12	17	
0.03	0.05	0.03	0.02	0.06	0.05	0.03	1.96	17	
0.72	0	0.4	0.19	0.02	0.38	0	0.63	17	
0.02	0.6	0.05	0.06	0.26	0.32	1.98	0.01	17	
0.29	0.19	0.15	0.18	0.41	0.4	1.98	0.19	17	
0.08	0.07	0.13	0.1	0.09	0.09	0.07	0.09	17	
0.04	0.41	0.22	0.1	0.25	0.37	0.28	0.03	17	
0.22	0.34	0.31	0.42	0.38	0.1	0.23	0.13	17	
0.31	0.12	0.16	0.05	0.12	0.06	0.16	0.05	17	
0.05	0.03	0.06	0.05	0.04	0.03	0	0.02	17	
0.22	0.12	0.24	0.09	0.04	0.23	0	0.03	17	
0.03	0.08	0.07	0.04	0.13	0.05	0.06	0.24	17	
0.09	0.23	0.24	0.12	0.32	0.13	0.04	0.03	17	
0.05	0.01	0.28	0.4	0.1	1.28	0.27	0.11	17	
0.19	0.04	0.09	0.03	0.31	0.1	0.28	0	17	
0.09	0.03	0.22	0.57	0.06	0.19	0	0.05	17	
0.71	0.45	0.26	0.13	0.32	0.1	0.18	0.1	17	
0.05	0	0.13	0.06	0.05	0.12	0.04	0.05	17	
0.47	0.01	0.24	0.14	0.07	0.16	0.08	0.02	17	
0.04	0.09	0.11	0.03	0.1	0.14	0.15	0.06	17	
0.05	0.03	0	0	0.07	0.03	0.02	0.07	17	
0.14	0.04	0.15	0.08	0.11	0.12	0.17	0.01	17	
0.09	0.04	0.1	0.02	0.16	0.12	0.17	0.07	17	
0.06	0.07	0.23	0.19	0.08	0.91	0.06	0.18	17	
0.09	0.04	0.2	0.3	0.1	0.4	0.19	0.09	17	
0.06	0.03	0.16	0.11	0.08	0.1	0.19	0.21	17	
0.03	0.05	0.19	0.6	0.02	0.07	0	0	17	
0.59	0.07	0.06	0.21	0.03	0.08	0.1	0.09	17	
0.01	0.02	0.02	0.03	0.04	0.05	0	0.06	17	
0.05	0.04	0.06	0.09	0.09	0.11	0.18	0.07	17	
0.02	0	0.02	0.04	0.06	0.03	0.08	0.01	17	
0.03	0.02	0.01	0.04	0.03	0.01	0.02	0.12	17	
0.02	0.01	0.01	0	0.01	0.02	0	0.01	17	
0.02	0.03	0.06	0.02	0.02	0.02	0	0.02	17	
0	0	0.01	0	0	0	0.03	0.01	17	
0.02	0.01	0.04	0.05	0.06	0.06	0.02	0.03	17	
0.02	0.1	0.13	0.17	0.14	0.09	0.06	0.07	17	
0.02	0.03	0.02	0.05	0.05	0.04	0.02	0.09	17	
0.04	0.15	0.08	0.03	0.13	0.08	0.03	0.08	17	
0.21	3.32	0.18	0.16	3.44	0.48	4.03	0.13	18	
0.07	3.16	0.1	0.19	3.2	0.28	4.23	0.17	18	
0.47	2.49	0.39	0.48	2.25	0.21	3.98	0.03	18	
0.86	3.33	0.11	0.46	3.17	0.23	3.92	0.13	18	
0.19	3.03	0.28	1.3	2.61	0.04	3.53	0.77	18	
0.06	1.43	0.73	1.94	1.29	0.12	4.21	0.48	18	
0	1.9	0.01	0	1.78	0.04	2.54	0	18	
0.03	2.27	0.11	0.06	2.4	0.03	3.33	0	18	
0.17	2.42	0.05	0.03	2.54	0.02	2.65	0.04	18	
0.03	2.27	0.01	0	2.41	0.02	1.43	0.03	18	
0.11	2.15	0.25	0.17	2.49	0.05	1.92	0.08	18	

0.02	2.45	0.04	0.04	2.88	0.1	2.9	0.02	18	
0.03	2.02	0.03	0	2.75	0.02	2.79	0.07	18	
0.07	2.67	0.05	0.03	3.08	0.2	3.28	0.06	18	
0.45	1.72	0.55	0.6	0.79	0.25	2.13	2.96	19	
0.29	1.02	0.11	0.13	0.81	0.33	3.58	3	19	
1.5	1.51	1.53	1.17	1.34	1.04	2.42	3.57	19	
0.37	0.56	0.41	0.34	0.28	0.61	1.24	3.34	19	
0.38	0.2	0.5	0.56	0.16	0.69	0.57	3.37	19	
0.87	1.14	0.99	0.92	1.34	0.41	0.49	2.86	19	
0.92	2.29	1.13	0.89	1.73	1.91	3.76	3.04	19	
0.77	2.92	1.11	0.65	3.16	1.81	3.64	3.31	19	
0.3	0.25	1.09	0.82	0.76	1.71	1.48	0.36	20	
0.54	0.43	2.1	0.71	0.44	1.28	0.16	0.26	20	
0.32	0.38	0.3	0.67	0.28	0.21	0.32	1.42	20	
0.83	0.12	0.69	0.56	0.33	0.58	0.36	1.83	20	
0.5	0.18	0.39	0.39	0.38	0.64	0.61	0.69	20	
0.53	0.19	0.53	0.42	0.43	0.69	1.08	0.8	20	
0.44	0.21	0.39	0.4	0.57	0.67	0.79	0.42	20	
0.58	0.28	0.47	0.43	0.54	0.75	0.85	0.63	20	
0.56	0.25	0.48	0.49	0.52	0.76	0.9	0.61	20	
0.81	0.37	0.69	0.8	0.94	1.23	1.05	1.12	20	
1.12	0.26	0.89	1.63	0.35	0.97	0.77	1.24	20	
1.21	0.99	0.82	1.14	1.05	0.92	0.93	0.3	20	
1.93	1.85	1.69	2.6	2.1	1.64	1.45	1.27	20	

Supplementary table 3

**Individual dataset**	raw names (GEO)	organism	platform	number of cells	Meta-dataset
Mouse incisors SS2	SS2_16_015, SS2_16_016, SS2_16_017, SS2_16_254,[illegible]	mouse	Smart-seq2	2889	Joint mouse
Mouse incisors 10x	mouse_incisor_pulp_S18	mouse	10x Chromium	4236	Joint mouse, Clodrosome/Healthy incisor
Mouse molars 1 10x	mouse_molar_pulp_S19	mouse	10x Chromium	384	Joint mouse
Mouse molars 2 10x	Mouse_molar_pulp_in_MeOH_11_9_9	mouse	10x Chromium	1460	Joint mouse
Mouse molar SS2	SS2_17_394, SS2_17_395	mouse	Smart-seq2	195	Joint mouse
Clodrosome-treated incisor 10x	Clodrosome	mouse	10x Chromium	2628	Clodrosome/Healthy incisor
Apical papilla 1	human_germinectomy	human	10x Chromium	1315	Joint human
Apical papilla 2	human_germ_molar_apical_papilla_female_15yo	human	10x Chromium	12522	Joint human
Adult molar 1	human_molar_healthy_1	human	10x Chromium	6057	Joint human
Adult molar 2	human_molar_healthy_2	human	10x Chromium	4876	Joint human
Adult molar 3	human_molar_pulp	human	10x Chromium	3386	Joint human
Adult molar 4	MF183_Dent	human	10x Chromium	309	Joint human
Adult molar 5	No5_24_yo_healthy_retained	human	10x Chromium	13208	Joint human

**Meta-dataset**	number of cells
Joint human	41673
Joint mouse	9164
Clodrosome/Healthy incisor	6864

Supplementary table 4

LIST OF NEWLY VALIDATED GENES IN TEETH

Gene	Type of a dataset	Validation method	Suggested function/position
**Igfbp5**	Mouse dental epithelium	In situ hybridization	Outer Enamel epithelium
**Piezo2**	Mouse dental epithelium	Immunohistochemistry	Subgroup of ameloblasts with mechanosensory potential
**Ryr2**	Mouse dental epithelium	Immunohistochemistry	Subgroup of ameloblasts with mechanosensory potential
**Egr1**	Mouse dental epithelium	Immunohistochemistry	Outer Enamel epithelium progenitors
**Fos**	Mouse dental epithelium	Lineage tracing (3 days, 10 days)	Outer Enamel epithelium progenitors
**Gjb3**	Mouse dental epithelium	In situ hybridization	Stellate reticulum
**Cldn10**	Mouse dental epithelium	Immunohistochemistry	Outer Enamel epithelium, Stellate reticulum, Stratum intermedium
**Acta2**	Mouse dental epithelium	Immunohistochemistry	Interphase between Stellate reticulum and Enamel epithelium
**Acta2**	Mouse dental epithelium	Lineage tracing (3 days, 2 weeks, 1 month, 2 months	Dental epithelial stem cells and traced progeny
**Thbd**	Mouse dental epithelium	Immunohistochemistry	Cuboidal layer of stratum intermedium
**Calb1**	Mouse dental epithelium	Immunohistochemistry	Secretory and Maturation ameloblasts
**Smoc2**	Mouse dental mesenchyme	Immunohistochemistry	Apical pulp
**Smoc2**	Human adult and growing	Immunohistochemistry	Apical papilla around the Hertwig epithelial root sheath
**Igfb5**	Mouse dental mesenchyme	In situ hybridization	Distal pulp
**Syt6**	Mouse dental mesenchyme	In situ hybridization	Distal pulp
**Tac1**	Mouse dental mesenchyme	In situ hybridization	Dental pulp around lingual cervical loop
**Sfrp2**	Mouse dental mesenchyme	In situ hybridization	Apical pulp and dental follicle
**Sfrp2**	Human adult and growing	Immunohistochemistry	Apical papilla around the Hertwig epithelial root sheath
**Sox9**	Mouse dental mesenchyme	Immunohistochemistry	Dental pulp (not in dental follicle)
**Dlx5**	Mouse dental dataset	Immunohistochemistry	Dental pulp (not in dental follicle)
**Foxd1**	Mouse dental mesenchyme	In situ hybridization	Dental pulp around labial cervical loop
**Foxd1**	Mouse dental mesenchyme	Lineage tracing (5 days, 1 month, 3 months)	Dental mesenchymal stem cells and traced progeny
**Notum**	Mouse dental mesenchyme	Immunohistochemistry	Odontoblasts
**Smpd3**	Mouse dental mesenchyme	In situ hybridization	Preodontoblasts
**Dkk1**	Mouse dental mesenchyme	In situ hybridization	Early odontoblasts
**Wnt6**	Mouse dental mesenchyme	In situ hybridization	Early and more differentiated odontoblasts
**Wisp1**	Mouse dental mesenchyme	In situ hybridization	Differentiated odontoblast
**Nupr1**	Mouse dental mesenchyme	In situ hybridization	Late odontoblasts
**Sall1**	Mouse dental mesenchyme	Immunohistochemistry	Preodontoblasts and odontoblasts
**Postn**	Human adult and growing	Immunohistochemistry	Periodontal ligament; Cell rich and cell-free zone of human dental pulp
**S100a13**	Human adult and growing	Immunohistochemistry	Odontoblasts
**Mki67**	Human adult and growing	Immunohistochemistry	Apical papilla around the Hertwig epithelial root sheath
**Aif1**	Mouse dental immune cells	Immunohistochemistry	Macrophages - Present everywhere in dental pulp and in cervical loops
**Aif1**	Mouse dental immune cells	Immunohistochemistry	Macrophages - Present in labial cervical loop and in attached mesenchyme
**Aif1**	Mouse dental immune cells	Immunohistochemistry	Macrophages - Present in labial cervical loop and direct contact to epithelial progenitors
**Aif1**	Mouse dental immune cells	Immunohistochemistry	Macrophages - P3 mandibles, Present everywhere in dental pulp and in cervical loops
**Aif1**	Human adult and growing	Immunohistochemistry	Macrophages in human dental pulp in various positions
**Lyve1**	Mouse dental immune cells	Immunohistochemistry	Subgroup of Macrophages - Present in the middle part of the pulp, not penetrating the odontoblast layer
**Lyve1**	Mouse dental immune cells	Immunohistochemistry	Subgroup of Macrophages - P3 mandibles, Present in the middle part of the pulp, not penetrating the odontoblast la
**Lyve1**	Human adult and growing	Immunohistochemistry	Subgroup of Macrophages in human dental pulp (scarse)
**Dpp4**	Mouse dental immune cells	Immunohistochemistry	NK cells - Sparse at apical part of the pulp and odontoblast layer
**Csf1**	Mouse dental dataset	In situ hybridization	Around and in odontoblasts layer; inside labial cervical loop
**Csf1**	Human adult and growing	Immunohistochemistry	Odontoblasts
**Csf1r**	Mouse dental dataset	In situ hybridization	Macrophages - Present everywhere in dental pulp and in cervical loops

Supplementary table 5

DISTAL INCISOR GENES									
gene symb	exp_10x_a	exp_10x_d	tscore_10x	p_10x	exp_ss2_a	exp_ss2_d	tscore_ss2	p_ss2	type
FN1	0.000165	0.000859	-11.9082	4.84E-29	0.000239	0.001399	-12.3258	2.81E-27	dist
IRS1	7.77E-05	0.000542	-10.1744	4.11E-22	0.000141	0.000454	-8.60201	4.77E-16	dist
FMOD	2.28E-05	0.001269	-17.1693	6.93E-50	0.000179	0.006563	-21.0242	1.90E-54	dist
CFH	1.83E-05	0.000309	-8.87314	1.94E-17	3.75E-05	0.000909	-16.7687	2.89E-41	dist
PROSER2	6.85E-06	0.000206	-7.46224	5.22E-13	1.04E-05	0.000131	-12.0743	2.15E-26	dist
ST8SIA6	4.57E-06	0.0003	-8.53661	3.13E-16	1.64E-05	0.000449	-10.9056	1.98E-22	dist
FBN1	9.14E-05	0.000362	-6.67576	5.67E-11	0.000146	0.000643	-9.79247	1.56E-19	dist
TSHZ2	1.83E-05	0.000188	-6.7569	4.45E-11	1.46E-05	7.48E-05	-7.10223	1.18E-11	dist
HEY1	1.60E-05	0.000427	-9.1542	2.66E-18	3.97E-05	0.000661	-14.7415	8.56E-35	dist
ATP1A1	0.000382	0.00274	-17.9519	7.90E-55	0.000444	0.002393	-15.9131	6.88E-39	dist
PDLIM5	1.14E-05	0.000315	-9.27799	9.95E-19	2.43E-05	0.000348	-13.0587	2.35E-29	dist
TNC	2.28E-05	0.002746	-26.741	2.88E-90	7.93E-05	0.002934	-20.0094	1.54E-51	dist
COL9A2	0.000288	0.000983	-9.96659	1.37E-21	0.000108	0.000428	-9.08031	3.32E-17	dist
SDC3	0.000107	0.000965	-13.9828	3.41E-37	7.92E-05	0.000645	-13.8489	1.70E-32	dist
STMN1	9.14E-05	0.000336	-6.65257	7.09E-11	2.54E-06	1.50E-05	-8.77831	7.12E-17	dist
ALPL	8.00E-05	0.000456	-8.75343	3.07E-17	0.000145	0.002026	-12.2092	1.64E-26	dist
MEGF6	0.000121	0.00048	-7.57174	1.71E-13	7.41E-05	0.000223	-8.39242	1.70E-15	dist
FGFR3	4.34E-05	0.000324	-7.58805	1.68E-13	0.000107	0.000678	-14.9724	1.97E-36	dist
KIT	1.37E-05	0.000333	-8.94582	1.22E-17	2.25E-06	0.000187	-10.6907	1.15E-21	dist
PLXND1	8.68E-05	0.000724	-10.986	3.94E-25	3.59E-05	0.000156	-9.53051	1.28E-18	dist
LDHB	0.000208	0.001071	-13.0022	2.59E-33	0.000431	0.001694	-15.9709	4.61E-41	dist
IFITM1	0.000183	0.001292	-13.8662	7.56E-37	0.000259	0.000915	-8.94847	1.26E-16	dist
AKAP12	9.14E-06	0.000203	-7.32139	1.29E-12	2.58E-05	0.00045	-15.42	2.43E-37	dist
GJA1	5.94E-05	0.00033	-7.24236	1.71E-12	0.000141	0.001239	-13.0471	1.17E-29	dist
NUDT4	0.000219	0.001648	-17.5714	1.39E-53	0.000536	0.004278	-21.6724	2.06E-58	dist
DCN	1.83E-05	0.000274	-7.81266	4.64E-14	0.00048	0.001995	-11.4918	5.25E-25	dist
LUM	0.000206	0.002873	-21.1887	2.34E-69	0.001357	0.010072	-15.5197	5.57E-38	dist
SLC20A2	5.03E-05	0.001207	-16.1813	8.00E-46	0.000151	0.002836	-14.0056	1.70E-32	dist
FGFR1	0.000338	0.001027	-10.885	2.01E-25	0.000671	0.002339	-16.7032	6.70E-43	dist
TGM1	2.28E-06	0.000174	-6.65398	9.73E-11	8.25E-06	0.00023	-9.47064	4.87E-18	dist
SCARA3	3.20E-05	0.000415	-10.2591	2.95E-22	0.00016	0.001778	-17.9475	2.00E-45	dist
CLU	1.14E-05	0.000253	-7.17155	3.49E-12	4.50E-05	0.000897	-10.2647	1.86E-20	dist
COL12A1	9.37E-05	0.000391	-7.00617	6.77E-12	0.000151	0.000524	-6.92044	3.69E-11	dist
PRSS35	2.97E-05	0.000259	-6.62845	8.65E-11	5.68E-05	0.000665	-14.0168	1.05E-32	dist
ATP1B3	0.000233	0.001157	-12.0541	6.54E-30	0.000466	0.002514	-18.2362	4.87E-48	dist
STC2	2.28E-05	0.00043	-10.1034	1.22E-21	6.50E-05	0.000423	-10.3355	5.62E-21	dist
PRKCA	5.25E-05	0.000321	-7.17786	2.40E-12	3.32E-05	0.00023	-9.21147	1.41E-17	dist
TGFBI	0.000135	0.001466	-16.302	9.91E-48	0.001049	0.003443	-12.6746	3.38E-29	dist
LGMN	5.48E-05	0.000597	-11.2135	7.18E-26	5.78E-05	0.000728	-15.7631	2.40E-38	dist
TNFAIP2	1.14E-05	0.000233	-7.83333	4.10E-14	6.85E-06	0.000221	-8.68827	7.17E-16	dist
SEMA5A	3.43E-05	0.000318	-8.48759	2.87E-16	2.32E-05	0.000378	-11.9159	8.88E-26	dist
ENPP2	5.25E-05	0.001007	-14.03	5.48E-37	0.000458	0.003966	-16.5378	8.92E-42	dist
KCNJ15	0	0.000212	-8.44602	6.19E-16	1.64E-05	0.000621	-13.9928	3.29E-32	dist
PPIC	3.66E-05	0.000356	-8.95903	9.77E-18	0.000126	0.000671	-11.3492	3.11E-24	dist
FBN2	6.40E-05	0.000527	-9.8732	4.43E-21	3.44E-05	0.000231	-9.12	3.00E-17	dist
CHKA	0.000295	0.001339	-13.5515	4.31E-36	0.000288	0.00131	-14.037	7.00E-34	dist

APICAL INCISOR GENES									
gene symbol	exp_10x_api	exp_10x_dis	tscore_10x	p_10x	exp_ss2_api	exp_ss2_dis	tscore_ss2	p_ss2	type
SERPINE2	0.0004661	6.18E-05	9.1652287	1.50E-18	0.0003921	4.81E-05	12.341949	2.30E-30	apical
GREM2	0.0003907	8.83E-06	10.1265	1.68E-21	0.0001256	5.96E-07	9.5350612	2.08E-19	apical
NR4A2	0.0004638	0.0001089	7.4281454	4.75E-13	0.0002757	1.45E-05	10.28254	3.92E-22	apical
DLX1	0.0003838	6.48E-05	9.1120926	2.16E-18	0.0003369	2.12E-05	20.640455	3.47E-65	apical
FRZB	0.0002879	5.30E-05	6.8582136	1.92E-11	0.0008276	8.54E-05	11.410921	2.87E-27	apical
THBS1	0.000738	5.59E-05	10.855891	2.25E-24	0.0005101	8.31E-05	6.857965	1.85E-11	apical
CPXM1	0.0007037	8.24E-05	11.811948	3.97E-28	0.0013213	0.0001469	15.948127	1.55E-44	apical
THBD	0.0002947	2.35E-05	7.8499616	3.33E-14	0.0002447	1.45E-05	11.312127	5.15E-26	apical
PFN2	0.0003427	7.65E-05	6.9831305	8.89E-12	0.0009436	0.0002243	11.708634	1.75E-28	apical
SFRP2	0.0053509	0.0001943	15.122128	8.51E-41	0.0062957	2.78E-05	12.67488	5.57E-31	apical
DDIT4L	0.0001599	2.94E-06	6.7489198	5.46E-11	0.0007092	1.24E-05	16.498069	1.12E-46	apical
JUN	0.0010213	0.0003149	10.117238	3.24E-22	0.0004289	8.45E-05	9.1026216	2.40E-18	apical
COL8A2	0.0001874	1.18E-05	6.7488266	5.00E-11	0.0004631	1.35E-05	16.964262	1.28E-48	apical
BHLHE40	0.0002422	1.18E-05	7.8273893	4.72E-14	9.41E-05	7.94E-06	7.8782824	2.78E-14	apical
CCND2	0.0012109	0.0001678	14.227337	1.88E-38	0.0010691	0.0001125	23.207654	2.84E-78	apical
BHLHE41	0.0002033	1.18E-05	7.0153466	1.01E-11	0.0005823	8.40E-06	18.568494	9.21E-55	apical
PEG3	0.0006077	0.0001913	6.9116672	1.12E-11	0.0004516	0.0001111	12.897307	1.79E-33	apical
FOSB	0.0024241	0.0004386	16.183789	2.22E-48	0.0003338	2.00E-05	10.152219	1.42E-21	apical
ZFP36	0.00061	0.0001001	8.279208	1.20E-15	0.0010266	8.24E-05	12.822696	1.44E-31	apical
NDN	0.0002216	3.53E-05	6.6260317	8.76E-11	0.0009415	0.0001758	16.091483	1.58E-46	apical
LSP1	0.0003838	4.71E-05	8.7405627	3.87E-17	0.0004343	5.62E-05	11.438822	1.70E-26	apical
CCND1	0.0004204	4.12E-05	9.8416581	1.18E-20	0.0004207	8.67E-06	13.271164	5.91E-35	apical
CPE	0.0003107	3.83E-05	7.6660171	1.13E-13	0.0005136	0.0001361	7.7040312	6.68E-14	apical
JUND	0.0011972	0.0003149	12.136213	2.69E-30	6.07E-05	1.26E-05	11.329617	1.93E-26	apical
KLF2	0.0005186	7.36E-05	9.0385769	4.38E-18	0.0001358	9.56E-06	9.7356148	3.97E-20	apical
IER2	0.0012795	0.0002208	12.878355	1.18E-32	0.0006617	4.39E-05	15.490247	1.07E-42	apical
JUNB	0.0013503	0.0002414	13.998963	2.00E-37	0.0009323	7.48E-05	19.186931	2.68E-58	apical
SHISA2	0.0006649	4.42E-05	10.351376	2.05E-22	0.001258	0.0001089	16.564133	5.17E-48	apical
GFRA2	0.0003564	7.65E-05	7.3085761	1.20E-12	0.0002029	5.65E-05	8.3286006	9.47E-16	apical
NCAM1	0.000722	6.77E-05	10.036244	1.79E-21	0.0005843	9.58E-05	12.47374	1.24E-30	apical
IGFBP3	0.0075946	0.0001678	23.734624	5.84E-77	0.0094658	0.0003802	18.967796	2.30E-57	apical
MFAP4	0.0008476	7.36E-05	13.901032	1.94E-36	0.0024209	9.99E-05	26.796352	1.16E-91	apical
WFIKKN2	0.0003724	1.18E-05	9.2808853	1.19E-18	0.0006186	8.78E-06	21.391648	1.80E-66	apical
IGFBP4	0.0055611	0.0008006	23.390432	1.16E-79	0.0065192	0.0005122	24.620958	4.49E-81	apical
FOXF2	0.000313	5.30E-05	7.4617712	4.00E-13	0.0002998	7.04E-05	10.523532	1.19E-23	apical
PTCH1	0.0010396	0.0001737	12.624485	1.46E-31	9.97E-05	1.17E-05	9.7572037	1.11E-20	apical
RHOB	0.000393	0.000106	7.4599332	3.65E-13	0.0001861	4.27E-05	11.470794	4.87E-27	apical
SOSTDC1	0.0005552	3.83E-05	10.086468	1.61E-21	0.0009956	3.51E-05	15.011824	2.76E-40	apical
PAX9	0.0002308	1.77E-05	7.7176721	7.96E-14	0.0001402	6.34E-06	13.707383	4.88E-35	apical
FOS	0.0042131	0.0011391	17.529014	6.46E-55	0.0066455	0.0004773	19.049313	6.78E-58	apical
BCL11B	0.0002513	2.35E-05	7.7834109	5.78E-14	7.30E-05	1.87E-06	8.9964905	1.07E-17	apical
DLK1	0.0009116	1.77E-05	11.731373	2.47E-27	0.0002549	5.25E-06	14.469384	4.87E-38	apical
MEG3	0.0028354	0.0005033	17.784478	6.95E-55	0.0002497	4.19E-05	12.70904	1.27E-32	apical
IGFBP6	0.0002742	2.06E-05	8.8345915	2.78E-17	0.0002977	5.61E-06	21.064151	2.77E-65	apical
COL8A1	0.0003016	8.83E-06	9.3720755	6.48E-19	0.0006657	5.62E-07	16.639993	1.29E-46	apical
SMOC2	0.0052161	0.0001854	22.900938	4.91E-74	0.0120399	2.30E-05	31.230866	1.69E-105	apical
DUSP1	0.0008728	0.0001118	11.155794	9.96E-26	0.0007486	5.16E-05	10.35781	2.40E-22	apical
RUNX2	0.0004958	9.12E-05	9.0294006	4.30E-18	0.0003804	8.44E-05	8.4746204	2.42E-16	apical
VIT	0.0003953	8.83E-05	7.0980443	4.31E-12	0.0002648	2.52E-05	10.306742	3.55E-22	apical
EGR1	0.0031964	0.0006564	17.679043	1.84E-54	0.0031051	0.0001127	19.98989	7.67E-62	apical
