# Supplementary material for: Dental cell type atlas reveals stem and differentiated cell types in mouse and human teeth
Source: Nat Commun. 2020 Sep 23;11:4816. doi: 10.1038/s41467-020-18512-7 (PMC7511944; doi:10.1038/s41467-020-18512-7)
Supplement: Supplementary file 2 — Reporting Summary [file 41467_2020_18512_MOESM2_ESM.pdf]

nature research

Corresponding author(s): Igor Adameyko, Peter V. Kharchenko

# Reporting Summary

Nature Research wishes to improve the reproducibility of the work that we publish. This form provides structure for consistency and transparency in reporting. For further information on Nature Research policies, see Authors & Referees and the Editorial Policy Checklist.

## Statistical parameters

When statistical analyses are reported, confirm that the following items are present in the relevant location (e.g. figure legend, table legend, main text, or Methods section).

| n/a | Confirmed | |
|---|---|---|
| x | | The exact sample size (*n*) for each experimental group/condition, given as a discrete number and unit of measurement |
| x | | An indication of whether measurements were taken from distinct samples or whether the same sample was measured repeatedly |
| | x | The statistical test(s) used AND whether they are one- or two-sided<br>*Only common tests should be described solely by name; describe more complex techniques in the Methods section.* |
| | x | A description of all covariates tested |
| | x | A description of any assumptions or corrections, such as tests of normality and adjustment for multiple comparisons |
| | x | A full description of the statistics including central tendency (e.g. means) or other basic estimates (e.g. regression coefficient) AND variation (e.g. standard deviation) or associated estimates of uncertainty (e.g. confidence intervals) |
| | x | For null hypothesis testing, the test statistic (e.g. *F*, *t*, *r*) with confidence intervals, effect sizes, degrees of freedom and *P* value noted<br>*Give P values as exact values whenever suitable.* |
| x | | For Bayesian analysis, information on the choice of priors and Markov chain Monte Carlo settings |
| | x | For hierarchical and complex designs, identification of the appropriate level for tests and full reporting of outcomes |
| | x | Estimates of effect sizes (e.g. Cohen's *d*, Pearson's *r*), indicating how they were calculated |
| | x | Clearly defined error bars<br>*State explicitly what error bars represent (e.g. SD, SE, CI)* |

*Our web collection on statistics for biologists may be useful.*

## Software and code

Policy information about availability of computer code

| | |
|---|---|
| Data collection | Data were collected according to smart-seq2 and 10x Chromium system. |
| Data analysis | STAR aligner v2.5; R programming language; PAGODA v1.99.4 and PAGODA2 v0.1.1 R packages; Rtsne v0.14 R package; hclust(), cutree() and lm() functions from stats v3.4.1 R package; lm() R function; mgcv v1.8-18 R package; pcaMethods v1.60.0 R package; ica v1.0-1 R package; RNA velocity v0.6 R package; crestree R package; CONOS v1.3.0 R package; scVI v0.6.5 Python package; Cell Ranger v2.2.0 and v3.0.2 10x Chromium software; BD FACSDiva 8.0.1; Imaris 8.3-9.5; FlowJo |

For manuscripts utilizing custom algorithms or software that are central to the research but not yet described in published literature, software must be made available to editors/reviewers upon request. We strongly encourage code deposition in a community repository (e.g. GitHub). See the Nature Research guidelines for submitting code & software for further information.

## Data

Policy information about availability of data

All manuscripts must include a data availability statement. This statement should provide the following information, where applicable:

- Accession codes, unique identifiers, or web links for publicly available datasets
- A list of figures that have associated raw data
- A description of any restrictions on data availability

All single-cell RNA-seq datasets have been deposited in the GEO under accession code GSE146123. Processed data, code, supplementary materials, and interactive views of datasets can be accessed on the authors' website:
http://pklab.med.harvard.edu/ruslan/dental.atlas.html

# Field-specific reporting

Please select the best fit for your research. If you are not sure, read the appropriate sections before making your selection.

- [x] Life sciences
- [ ] Behavioural & social sciences
- [ ] Ecological, evolutionary & environmental sciences

For a reference copy of the document with all sections, see nature.com/authors/policies/ReportingSummary-flat.pdf

# Life sciences study design

All studies must disclose on these points even when the disclosure is negative.

| | |
|---|---|
| Sample size | Total amount of sequenced cells was 53465 including both mouse and human tissue. Sample size was sufficient to elucidate not only major populations, but we were able to find also very small subpopulations of different origin. Data were consistent when compared to different samples analyzed in this study. |
| Data exclusions | Cells which didn't pass the quality check were excluded from the analysis in an unbiased way. CellRanger- 10x Chromium software was used to perform alignment to GRCh38 human genome or mm10 mouse genome assemblies, filtering, barcode counting and UMI counting. For Apical papilla 1, Adult molar 3, Adult molar 4, incisor (10x), mouse molar 1 (10x) datasets pre-processing was performed using CellRanger-2.2.0 following by filtering of cells having less than 500 UMIs. For other datasets datasets pre-processing was performed using CellRanger 3.0.2 following by default CellRanger 3.0.2 filtering of cells. Additionally, a protocol of library preparation used by the facility included spike-in of Jurkat and 32D cells of human and mouse species. Spike-in cells were not used for data processing or analysis and were excluded as Hbb+ clusters; they are also easily detectable as having low complexity and forming a separate outlier transcriptional cluster." |
| Replication | All the sequenced single cells were merged together into one complex ubiased analysis. Reproducibility of experimental findings was fully proved when compared to different sequencing methods. |
| Randomization | Cells were isolated from mouse incisors/molars (adult animals aged between 2-4 months to ensure reproducibility of the results) and human growing/non-growing/caries molar. No specific selection during isolation was applied. For analysis of adult healthy mouse incisor 78 mandibular incisors were used out of 39 animals in total. For analysis of mouse molar pulps 48 first molars out of 12 adult animals were used in total. For adult human tooth analyses 7 wisdom molars out of 7 healthy randomly selected males and females of age 18-31 were used and 6 apical papillae out of 3 patients/teeth were used. |
| Blinding | All the bioinformatic parts of single cell clustering were made in fully unbiased manner. Outcomes were then analyzed by biologists. Dentists randomly selected molars from healthy males and females of age 18-31. All the data were subsequently merged together and no significant differences between samples out of different patients was observed, blinding was not relevant for this study. |

# Reporting for specific materials, systems and methods

### Materials & experimental systems

| n/a | Involved in the study |
|---|---|
| x | Unique biological materials |
| | x Antibodies |
| x | Eukaryotic cell lines |
| x | Palaeontology |
| | x Animals and other organisms |
| | x Human research participants |

### Methods

| n/a | Involved in the study |
|---|---|
| x | ChIP-seq |
| | x Flow cytometry |
| x | MRI-based neuroimaging |

# Antibodies

Antibodies used

ACTA2 (Protein Tech, 23081-1-AP; 1:500), AIF1 (Novus, NB100-1028; 1:500), CALB1 (Swant; CB-38a; 1:500), COL4 (AbD Serotec, 2150-1470; 1:500), CDH1 (Novus, Af748; 1:500), CSF1 (NSJ, R31901; 1:200), CLDN10 (Sigma Aldrich, HPA042348; 1:200), DLX5 (LSbio, LS-C352119; 1:200), DPP4 (Novus, AF954; 1:200), EGR1 (Cell Signalling, 4154; 1:200), F4/80 (Abcam, ab6640; 1:200)LYVE1 (Novus, AF2125; 1:200), MKI67 (Zytomed, RBK027-05, 1:200), NOTUM (Sigma Aldrich, HPA023041; 1:200), PIEZO2 (Sigma Aldrich, HPA040616; 1:200), POSTN (Novus, NBP1-30042; 1:200), RYR2 (ThermoFisher, PA5-36121; 1:200), S100A13 (DAKO; IS504; 1:500), SALL1 (Abcam, ab31526; 1:200), SMOC2 (MyBioSource, MBS2527784; 1:200), SOX9 (Sigma Aldrich, HPA001758; 1:200), SOX10 (Santa cruz, sc-365692; 1:200), THBD (RnD systems, MAB3894; 1:200)

Validation

ACTA2 Supplier webpage – proved to be working on immunohistochemical staining of paraffin-embedded human heart. Proved mouse reactivity. https://www.ptglab.com/products/ACTA2-Antibody-23081-1-AP.htm

AIF1 Supplier webpage – proved to be working on immunohistochemistry-Paraffin sample of mouse brain. https://www.novusbio.com/products/aif-1-iba1-antibody_nb100-1028

CALB1 Proved to be working on Rat brain tissue section (postnatal day 21) IHC - https://www.biocompare.com/Product-Reviews/331113-Excellent-for-immunostaining/

COL4 Proved to be working in (Lange et al., 2012)

CDH1 Supplier webpage – E-Cadherin was detected in immersion fixed mouse intestinal organoids. https://www.novusbio.com/products/e-cadherin-antibody_af748

CSF1 Supplier webpage – proved to be working on IHC of FFPE mouse spleen. https://www.nsjbio.com/tds/csf-1-antibody-r31901

CLDN10 Supplier webpage – proved to be working on immunohistochemistry analysis in human kidney and lymph node tissues.

DLX5 Supplier webpage – proved to be working in immunohistochemical staining in human lung cancer formalin fixed paraffin embedded tissue section. Confirmed mouse reactivity. https://www.lsbio.com/antibodies/anti-dlx5-antibody-internal-icc-if-immunofluorescence-ihc-ip-wb-western-ls-c352118/363239

DPP4 Supplier webpage – proved to be working in IHC of fixed frozen sections of mouse thymus. https://www.novusbio.com/products/dppiv-cd26-antibody_af954

EGR1 Supplier webpage – proved to be working in immunofluorescent analysis of dissociated PC12 cells. https://www.cellsignal.com/products/primary-antibodies/egr1-44d5-rabbit-mab/4154

F4/80 Supplier webpage – proved to be working in IHC-Fr: Mouse spleen and intestine tissue

LYVE1 Supplier webpage – proved to be working in IHC analysis of frozen sections of mouse liver. https://www.novusbio.com/prducts/lyve-1-antibody_af2125

MKI67 Supplier webpage – proved to be working in IHC, proved to be working in Breast carcinoma, tonsil, and spleen

NOTUM Supplier webpage – proved to be working on Immunohistochemical staining of human kidney https://www.sigmaaldrich.com/catalog/product/sigma/hpa023041?lang=de&region=AT

PIEZO2 Proved to be working in (Khatibi Shahidi et al., 2015)

POSTN Supplier webpage – proved to be working on Immunohistochemical staining of rabbit periosteum

RYR2 Supplier webpage – proved to be working in paraffin-embedded imunohistochemical analysis of human colorectal carcinoma. Proved mouse reactivity. http://www.thermofisher.com/order/genome-database/details/antibody/PA5-36121.html?CID=AFLAP-PA5-36121

S100A13 Supplier webpage - proved to be working http://www.visabl.com/detail/s-100/polyclonal-dako

SALL1 Supplier webpage – proved to be working in SALL1 staining of metanephric blastema cells, murine kidney tissue by Immunohistochemistry (Formalin/PFA-fixed paraffin-embedded sections). http://www.abcam.com/sall1-antibody-ab31526.html#description_images_2

SMOC2 Supplier webpage – proved to be working for immunohistochemistry of paraffin-embedded Human cervical cancer. https://www.mybiosource.com/prods/Antibody/Polyclonal/SMOC2/datasheet.php?products_id=2527784

SOX9 Supplier webpage – proved to be working for immunohistochemical staining of human glioma. https://www.sigmaaldrich.com/catalog/product/sigma/hpa001758?lang=de&region=AT

SOX10 Proved to be working in (Kaukua et al., 2014)

THBD Supplier webpage – proved to be working on mouse endothelioma cell line (ICC/IF) https://www.novusbio.com/products/thrombomodulin-bdca-3-antibody-461714_mab3894

# Animals and other organisms

Policy information about studies involving animals; ARRIVE guidelines recommended for reporting animal research

Laboratory animals

FosCreERT2/R26ZsGreen – Both males and females. Age between 2-4 months.
DSPP-cerualean/DMP1-Cherry – Both males and females. Age between 2-4 months.
Acta2CreERT2/R26tdTomato – Both males and females. Age between 2-4 months.
Sox2-GFP – Both males and females. Age between 2-4 months.
Foxd1CreERT2/Ai9 – Both males and females. Age between 2-5 months.
C57BL/6J – Both males and females. Age between 2-4 months.

All animal experiments were approved by the "Ethik-Kommission der MedUni Wien zur Beratung und Begutachtung von Forschungsprojekten am Tier" in Austria as well as Ethical Committee on Animal Experiments (Stockholm North Committee) in Sweden and performed according to the Austrian, UK, Swedish and international regulations. All mice were kept under SPF

conditions in 12/12 light/dark cycle, 18-23°C and 40-60% humidity. Experiments with human samples were performed with the approval of the Committees for Ethics of the Medical Faculty, Masaryk University Brno & St. Anne´s Faculty Hospital (No. 13/2013) and Ethik-Kommission der Medizinischen Universität Wien (No. 018/03/2018, 631/2007).

Wild animals

Study did not involve wild animals.

Field-collected samples

Study did not involve samples collected from the field.

# Human research participants

Policy information about studies involving human research participants

Population characteristics

According to BRISQ reporting guidelines:

Biospecimen type: Wisdom teeth from randomly selected healthy males and females of age 18-31.

Disease status of patients: 1) Healthy fully developed molar, 2) Healthy growing molar, 3) Molar with caries

Vital State of patients: Living patients

Clinical diagnosis of patients: Healthy teeth had to be extracted from orthodontical reasons and molar with caries because severe inflammation.

Pathology diagnosis: Healthy teeth had to be extracted from orthodontical reasons and molar with caries because severe inflammation.

Collection mechanism: Teeth extraction.
Type of stabilization: Single cells were isolated and fix in 90% methanol.

Type of long-term preservation: Suspension in 90% methanol was preserved at -80 °C until analysis.

Constitution of preservative: 90% methanol

Storage temperature: -80 °C

Storage duration: 1 day - 2 weeks

Recruitment

Teeth from all patients were extracted based on clinically-relevant reasons.

Healthy patients with no other relevant diagnosis were selected for adult and growing molars isolations.

Patients with only selection criteria: healthy males and females of age 18-31, were randomly selected by dentists and provided to researchers for the analyses. Self-selection bias is not applicable.

# Flow Cytometry

## Plots

Confirm that:

- [x] The axis labels state the marker and fluorochrome used (e.g. CD4-FITC).
- [x] The axis scales are clearly visible. Include numbers along axes only for bottom left plot of group (a 'group' is an analysis of identical markers).
- [x] All plots are contour plots with outliers or pseudocolor plots.
- [x] A numerical value for number of cells or percentage (with statistics) is provided.

## Methodology

Sample preparation

FACS technique was used for single cell sorting of living cells for the single cell transcriptomics.

Wildtype C56Bl6, PLPCreERT2/R26YFP and Sox2-GFP mice were used for cell isolation from mandibular incisors for single cell transcriptomics experiments. Age of all mice used for single cell experiments was between 2 and 4 months. Mice were sacrificed by isoflurane overdose. Mandibles were carefully dissected and under stereomicroscope and surrounding soft tissue was removed. Using scalpel and scissors mandibular bone was gradually removed and to obtain separated incisor. Particularly careful handling was performed in the soft area around the most proximal part of incisor where cervical loops are. Dental pulps with surrounding dental epithelium including cervical loops were isolated, cut into small pieces, transferred to 15 mL falcon tube with 2,5 mL Collagenase P (3 U/mL; Sigma Aldrich, COLLA-RO ROCHE) dissolved in HBSS (Sigma Aldrich, H6648) and incubated for 20 minutes at 37 °C shaking (120 rpm). During enzymatic digestion, tissue pieces were homogenized 2 times using 1 ml pipet. After incubation the suspension was finally homogenized using pipet and 10 mL of 2 % FBS (ThermoFisher Scientific, 10500064) in

HBSS were slowly added. The suspension was centrifuged in 4 °C precooled centrifuge for 10 minutes at 300 g. After centrifugation, supernatant was removed, the pellet was resuspended in 1 mL 2 % FBS in HBSS, suspension was filtered using Tubes with Cell Strainer Snap Cap (Corning, 352235) and FACS was performed. All the work (except of enzymatic digestions at 37 °C) was performed on ice.

| | |
|---|---|
| Instrument | BD FACSAria III Cell Sorter |
| Software | BD FACSDiva 8.0.1 |
| Cell population abundance | Quality of cells was ultimately determined by single cell transcriptomics itself. |
| Gating strategy | Three gating aspects were selected: a) SSC-A/FSC-A, b) FSC-A/FSC/W, c) SSC-A/SSC/W and strict gates were applied to remove debris, dead cells and doublets. When genetically traced organisms were used the fourth gate were applied during FACS sorting. Negative control using wildtype organism was applied to make a correct gating. |

☒ Tick this box to confirm that a figure exemplifying the gating strategy is provided in the Supplementary Information.
