# Supplementary material for: Dental cell type atlas reveals stem and differentiated cell types in mouse and human teeth
Source: Nat Commun. 2020 Sep 23;11:4816. doi: 10.1038/s41467-020-18512-7 (PMC7511944; doi:10.1038/s41467-020-18512-7)
Supplement: Supplementary file 3 — Description of Additional Supplementary Files [file 41467_2020_18512_MOESM3_ESM.pdf]

Description of Additional Supplementary Files

File Name: Supplementary Data 1

Description: Source data table with key gene identifiers used for unsupervised clustering and identification of all major clusters in general dataset.
